# New insight for pharmacogenomics studies from the transcriptional analysis of two large-scale cancer cell line panels

**DOI:** 10.1038/s41598-017-14770-6

**Published:** 2017-11-09

**Authors:** Benjamin Sadacca, Anne-Sophie Hamy, Cécile Laurent, Pierre Gestraud, Hélène Bonsang-Kitzis, Alice Pinheiro, Judith Abecassis, Pierre Neuvial, Fabien Reyal

**Affiliations:** 1 Residual Tumor & Response to Treatment Laboratory (RT2Lab), PSL Research University, Translational Research Department, F-75248 Paris, France; 2 U932 Immunity and Cancer; INSERM; Institut Curie, Paris, France; 3 Laboratoire de Mathématiques et Modélisation d’Evry, Université d’Évry Val d’Essonne, UMR CNRS 8071, ENSIIE, USC INRA, Evry Val d’Essonne, France; 4 Mines Paristech, PSL-Research University, CBIO-Centre for Computational Biology, Mines ParisTech, Fontainebleau, F-77300 France; 5 Institut Curie, PSL Research University, Mines Paris Tech, Bioinformatics and Computational Systems Biology of Cancer, INSERM U900, F-75005 Paris, France; 6 Department of Surgery, Institut Curie, Paris, F-75248 France; 7 Institut de Mathématiques de Toulouse; UMR5219 Université de Toulouse; CNRS UPS IMT, F-31062 Toulouse Cedex 9, France

## Abstract

One of the most challenging problems in the development of new anticancer drugs is the very high attrition rate. The so-called “drug repositioning process” propose to find new therapeutic indications to already approved drugs. For this, new analytic methods are required to optimize the information present in large-scale pharmacogenomics datasets. We analyzed data from the Genomics of Drug Sensitivity in Cancer and Cancer Cell Line Encyclopedia studies. We focused on common cell lines (n = 471), considering the molecular information, and the drug sensitivity for common drugs screened (n = 15). We propose a novel classification based on transcriptomic profiles of cell lines, according to a biological network-driven gene selection process. Our robust molecular classification displays greater homogeneity of drug sensitivity than cancer cell line grouped based on tissue of origin. We then identified significant associations between cell line cluster and drug response robustly found between both datasets. We further demonstrate the relevance of our method using two additional external datasets and distinct sensitivity metrics. Some associations were still found robust, despite cell lines and drug responses’ variations. This study defines a robust molecular classification of cancer cell lines that could be used to find new therapeutic indications to known compounds.

## Introduction

One of the most challenging problems in the development of new anticancer drugs is the very high attrition rate. Less than 5% of the drugs entering phase I trials eventually obtain marketing authorization^1^. Clinical trials are the only real way to assess drug efficacy and toxicity, but this approach is inadequate for testing the hundreds of drugs currently being developed^2^. Scientists need to test hundreds of drugs on numerous tumor models therefore frequently make use of tumor-derived cell lines^3–5^. Such studies aim to identify genomic biomarkers for predicting the responses of individual patients to the drug and, ultimately, for identifying the best drug for each patient.

In 2012, the first large-scale pharmacogenomics studies provided an unprecedented wealth to the scientific community. The Broad Institute-Cancer Cell Line Encyclopedia (CCLE) provided a collection of 1,036 human cancer cell lines from 36 tumor types, tested for 24 anticancer drugs. The Genomics of Drug Sensitivity in Cancer (GDSC) assessed the sensitivity of 727 cell lines, from 29 tissue types, to 138 drugs. Both datasets contain genome-wide gene expression and sequencing data for a subset of genes. These studies have provided unprecedented amounts of information about molecular profiles and drug sensitivity and have validated several known genetic biomarkers, such as the BRAF-V600E mutation sensitizing melanomas to vemurafenib^6^ or ERBB2 amplification/overexpression conferring sensitivity to lapatinib^7^.

Previous studies assessed drug sensitivity by pooling all the cell lines or by controlling for tissue source. However, with improvements in our knowledge about tumors, it has become clear that genomic, epigenomic, transcriptional, and proteomic analyses of a given cancer can reveal subtypes differing in pathway activity, progression or treatment response^8,9^. Conversely, the recent success of basket studies^10,11^ have demonstrated that treatment choices can be based on abnormalities shared by tumors originating from different tissue types.

We present here a comprehensive reanalysis of these two recently published large-scale pharmacogenomics resources. We propose an alternative approach in which cell lines are grouped by transcriptomic profile, based on a biological network-driven gene selection process. This molecular classification of cancer cell lines appeared robust across CCLE and GDSC. We further demonstrated the relevance of this novel classification through the drug response We validate our approach by robustly found in CCLE and GDSC as in two external dataset the significant associations between cell line clusters and drug responses.

## Results

### A biologically driven approach identifies four robust gene modules

**Figure 1:**
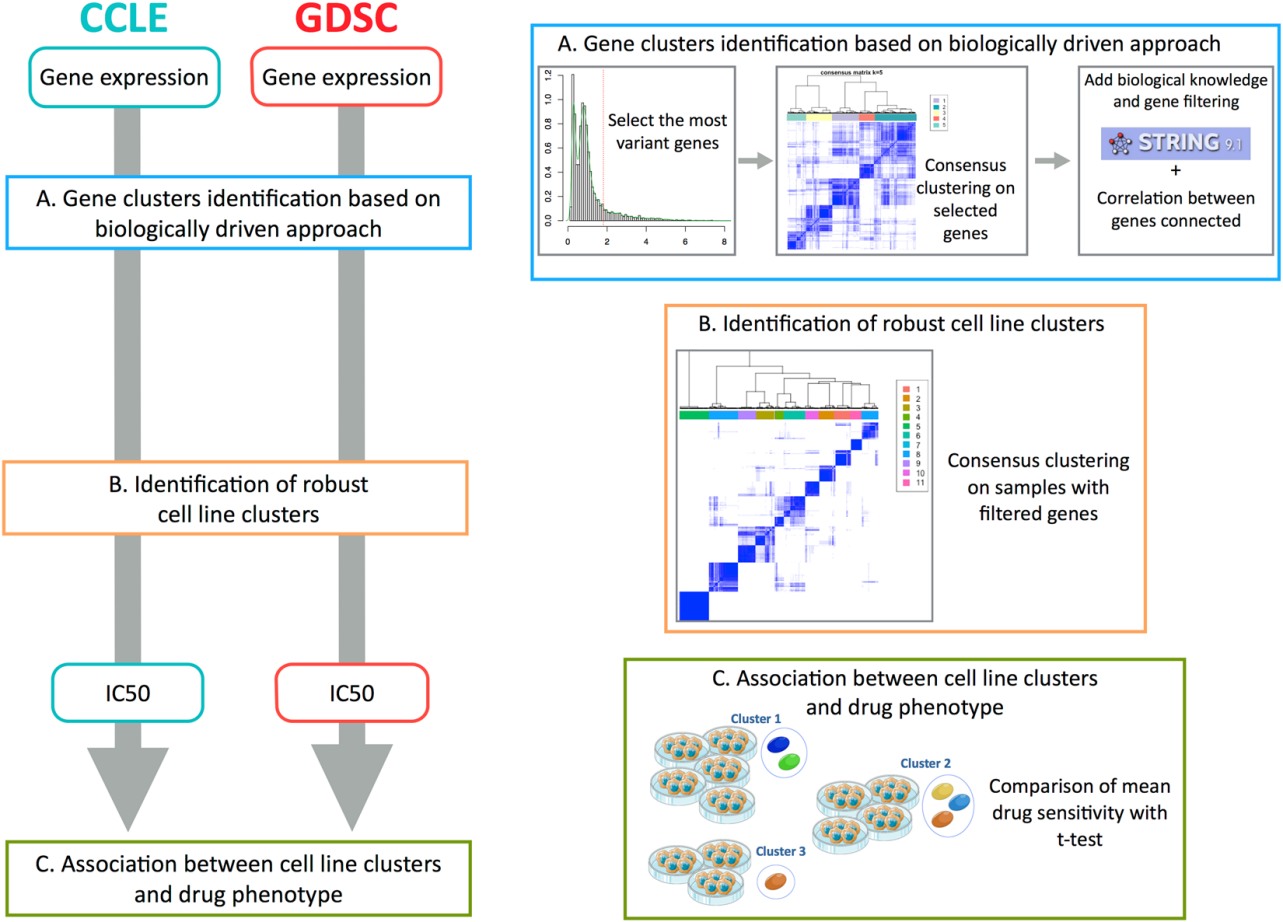
Flow chart of the analysis. We apply the same pipeline of analysis independently to CCLE and GDSC. (**a**) Biologically driven gene selection was performed to build robust clusters of genes. (**b**) Robust clusters of cell lines were then built using the selected genes. (**c**) Cell lines clusters have been associated to distinct drug response.

Gene expression profiles were recovered for 471 cell lines, from 24 different tissues, tested in both CCLE and GDSC. Data were curated and annotated with the pipeline of Haibe-Kains *et al*.^12^. We developed a three-step biological network-driven process based on transcriptomic data for identifying robust clusters of genes. This process was applied in parallel for each dataset. We first selected the most variant genes from the set of 12,153 genes common to GDSC and CCLE, by the inflexion point method. We then performed hierarchical consensus clustering^13^ to identify robust gene modules. Finally, we used String© database software^14^ to analyze our gene selection. The goal was to decrease the heterogeneity of each gene cluster. We retained the genes from our initial selection that had (1) high String© database gene connection indices (greater than 0.7), and (2) similar patterns of expression to other genes within the same biological network (correlation coefficient of at least 0.5) (Fig. 1 step A). This selection process identified four stable clusters in GDSC (*n* = 183 genes) and five in CCLE (*n* = 210 genes), including a subset of 170 genes common to the two datasets. Distinct functional gene ontologies were associated to each gene modules based on a gene ontology analysis: (Supplementary Fig. 1) Gene Cluster – Extracellular Matrix (GC-ECM; n_ccle = 48, n_GDSC = 36), Gene Cluster - Migration (GC-Migration; n_ccle = 56, n_GDSC = 75), Gene Cluster - Immunity-Interferon (GC-Immunity; n_ccle = 22, n_GDSC = 14) and Gene Cluster - Epithelial Phenotype (GC-Epithelial; n_ccle = 63, n_GDSC = 58). A set of 21 genes enriched in development processes (GC-Development) was found exclusively in the CCLE dataset.

### Biologically driven gene selection identifies eleven reproducible cell line clusters

**Figure 2:**
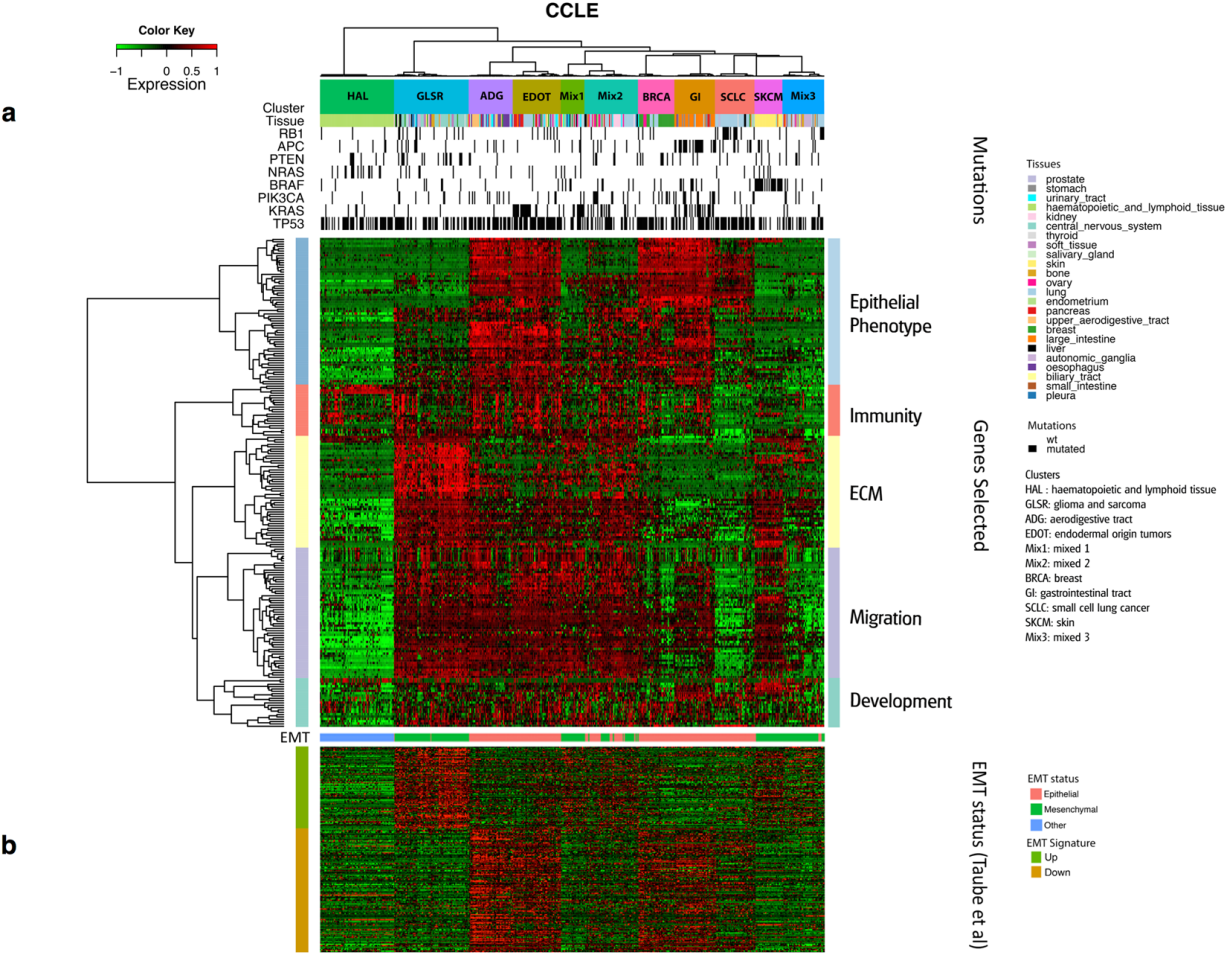
Cell line clustering with CCLE data. (**a**) Heatmap clustering with 471 cell lines (in columns) and 210 selected genes (in rows) for the CCLE data. (**b**) EMT status of the cell lines.

We performed a consensus clustering with the previously selected genes, for each dataset separately, to identify global differences in gene expression between cancer cell lines (Fig. 1 step B). We obtained eleven stable clusters of cell lines in CCLE and GDSC (Fig. 2a and Supplementary Fig. 2a).

**Figure 3:**
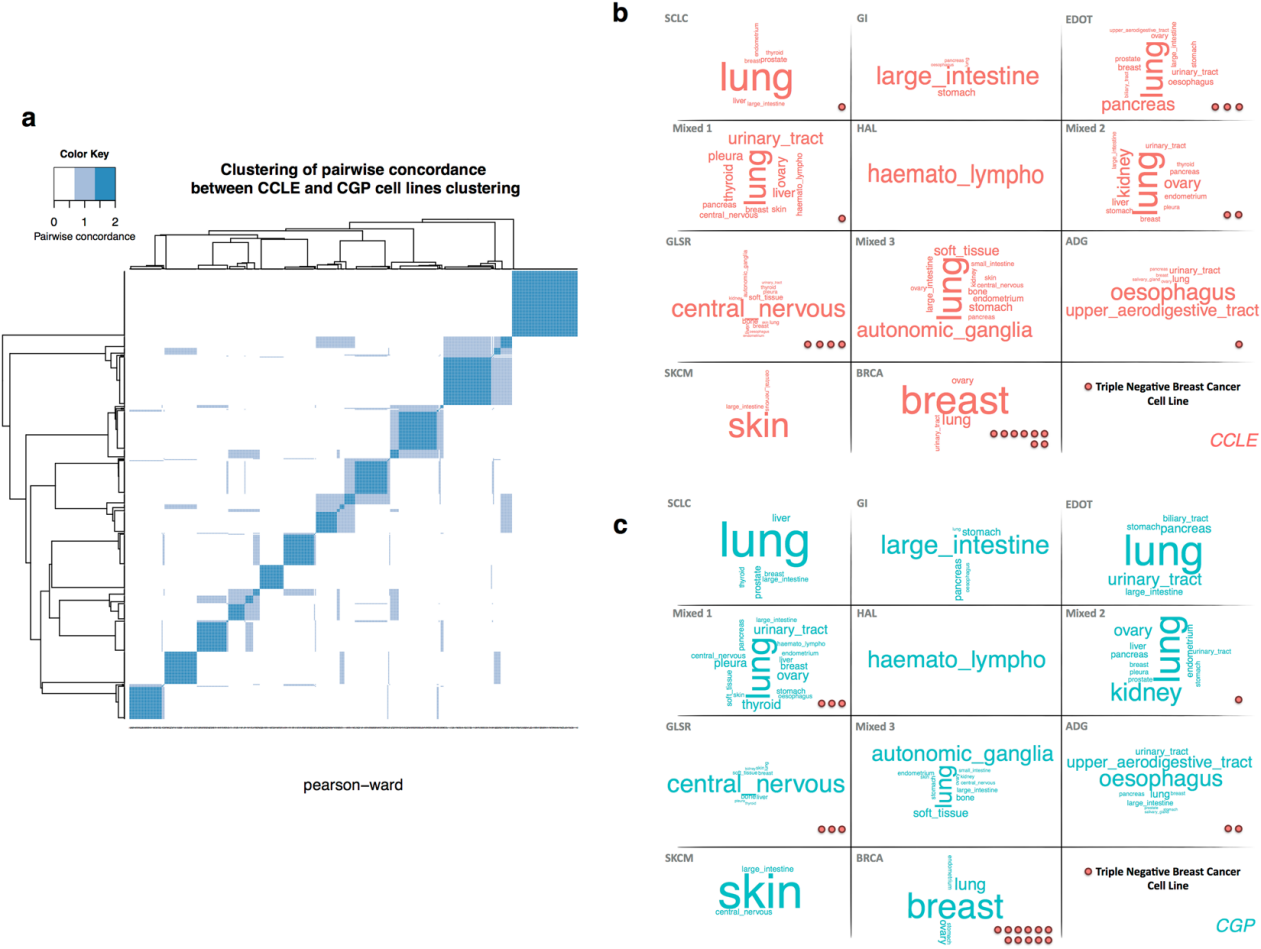
Clustering similarity. (**a**) Color-coded heatmap for similarity between CCLE and GDSC clustering; Tag Cloud represents the tissue composition of cell lines cluster, in CCLE (**b**) and GDSC (**c**). The importance of each tissue is indicated by font size. The TNBC cell lines belonging to each cluster are indicated by red dots.

Previous studies reported strong correlations between the expression profiles of identical cell lines^12^. We therefore investigated the closeness of the cell line clusters obtained. We defined the similarity between any two cell lines as the number of datasets in which they clustered together (0 = none, 1 = CCLE or GDSC, 2 = CCLE and GDSC). We assessed the consistency between the clustering patterns obtained with CCLE and GDSC data, using a heatmap clustering of the similarity matrix as a visualization tool. The heatmap shows the number of times that two samples are clustered together across datasets (Fig. 3a). Groups of cell lines that frequently cluster with each other are shown in darker shades of blue. The heatmap revealed a well defined 11-block, corresponding to the 11 clusters previously identified. A high degree of consistency between the 11 clusters was observed, with 90% accuracy. As the cell line clusters were highly similar, we use the term “cluster” to denote the same group of cell lines from CCLE and GDSC, unless the dataset is specified.

### Tissue-of-origin or transcriptomic features dominate cell line clusters

Our eleven clusters can be organized in three major patterns: (i) four clusters of cell lines were derived mostly from tumors from the same tissue of origin. These cell line clusters were named after the organ or cancer subtypes from which most of the cell lines were derived: hematopoietic and lymphoid tissues (HAL), small cell lung cancer (SCLC), skin (SKCM) and breast (BRCA) clusters; (ii) four clusters of cell lines were derived from tissues from the same organ system or had a common embryonic origin: gastrointestinal tract (GI), aerodigestive tract (ADG), glioma and sarcoma (GLSR) and endodermal origin tumors (EDOT) clusters; (iii) three clusters contained cell lines from different tissues of origin. These clusters were named Mixed 1, Mixed 2 and Mixed 3 (Fig. 3b and c. Details provided in Supplementary data 1 and 2).

#### Clusters of cell lines with common presumptive tissues of origin

Four cell line clusters appeared very homogeneous in terms of tissue lineage: HAL, SCLC, SKCM and BRCA. These lineages accounted for 84%, on average, of the cells of their respective clusters. The HAL cluster grouped together all the cell lines originating from hematopoietic and lymphoid tissues. This clear clustering pattern can be accounted for by the hematopoietic phenotype of this type of tumor. The SKCM cluster was the second most homogeneous cell line cluster in terms of tissue type (92% of the cell lines in this group originated from melanomas). Breast cancer is a heterogeneous disease with a growing number of recognized biological subtypes, including ER+ Her2−, Her2+ and triple-negative breast cancer (TNBC), which is the most aggressive subtype. BRCA cluster contained all the breast cancer cell lines defined as ER+ Her2− (7/7) and Her2+ (7/7). However, only about half the cell lines defined as triple-negative belonged to this cluster (11/20 in GDSC, 8/20 in CCLE). The remaining triple-negative breast cancer cell lines were found in six different clusters of cell lines (SCLC, EDOT, Mixed 1, Mixed 2, GLSR and ADG) (Fig. 3b,c). SCLC cluster contained 28% of the lung cancer cell lines and 45% of the small-cell lung carcinoma cell lines. We performed a Gene Set Enrichment Analysis^15^ (GSEA) based on our previously defined gene modules to characterized the transcriptomic profile of cell line clusters (Supplementary Fig. 3). The immunity gene module was strongly expressed in the cell lines of the HAL cluster. Leukemia affects both the bone marrow and lymphocytes, potentially accounting for the detection of immunity gene expression in cell lines derived from a tumor system with no stromal environment. In the SKCM cell line cluster, the epithelial phenotype gene module was downregulated. Furthermore, the activation of the ECM and migration gene modules in this cluster is suggestive of aggressive cancer. In the BRCA and SCLC cell line clusters, the epithelial gene module was expressed, whereas the migration and ECM gene modules were not.

#### Clusters of cell lines from tissues of the same organ system or common embryonic origin

Some clusters could not be defined on the basis of origin from a single tissue type. However, with a more systemic vision, a consistent organization was obtained for four clusters: GI, ADG, GLSR and EDOT. Cell lines derived from tumors of the digestive system belonged to two clusters. The ADG cell line cluster consisted mostly of tumors from the esophagus, upper aerodigestive tract, salivary and also urinary glands, whereas the GI cluster grouped together tumors derived from large intestine, stomach and pancreas cancers. About 70% of the cell lines of the GLSR cluster were derived from tumors of the central nervous system, bone, autonomic ganglia and soft tissue. Finally, the EDOT cell line cluster grouped together cell lines derived from tumors of different tissues (e.g. lung, pancreas, urinary tract) arising from the same germ layer (endoderm). The relevance of the EDOT cluster is supported by studies suggesting that oncogenesis may be initiated by the activation of a common pathway in an endodermal progenitor^16^.

The ADG, GI and EDOT clusters all displayed strong expression of the genes of the epithelial phenotype module and weak expression of the ECM gene module. According to GSEA, the migration gene module was less strongly expressed in GI cells. For the EDOT cluster, inconsistencies between the CCLE and GDSC datasets were observed concerning the activation or inhibition of migration gene expression at the transcriptomic level only. The GLSR cluster displayed low levels of expression for the epithelial gene module, and high levels of expression for the ECM and migration modules.

#### Clusters of cell lines from tumors with heterogeneous tissues of origin

Three clusters displayed no particular prevalence of cell lines corresponding to any particular tissue or organ system. They contained cell lines from tumors of 11 to 16 different tissues. We named these clusters Mixed 1, Mixed 2 and Mixed 3. All three of these clusters displayed low levels of epithelial phenotype genes, suggesting that the cell lines they contained were probably mesenchymal. These clusters also displayed an upregulation of ECM genes. Mixed 1 and 2 displayed an upregulation of migration gene expression. These results suggest that some of the cell lines may have been metastatic in origin or subject to drift, from the characteristics of the tissue of origin to a less differentiated state. In this case, transcriptomic profile is more relevant than tissue of origin.

### EMT discriminates between cell line clusters

The identification of an epithelial phenotype gene module led us to investigate the epithelial-mesenchymal status of each cell line. A previous study^17^ showed that epithelial/mesenchymal transition (EMT)-associated differences in gene expression were a major determinant of the stratification of cancer cell lines based on transcriptomic profiles. Indeed, we found a significant overlap between our gene selections and a published EMT-derived gene signature consisting of 249 genes^18^ (*P* < 0.0001, two-tailed Fisher’s exact test). We superimposed epithelial/mesenchymal cell line classifications over our gene expression clusters and found a strong association (Fig. 2b and Supplementary Fig. 3b). According to the EMT signature, five cell line clusters (SCLC, GI, EDOT, ADG and BRCA) contained mostly epithelial cell lines, whereas the Mixed 1, Mixed 3, GLSR and SKCM cell line clusters contained mostly mesenchymal cell lines. The Mixed 2 cell line cluster appeared to contain mostly mesenchymal cell lines in GDSC but almost half the cell lines assigned to this cluster in CCLE were epithelial. The HAL cell lines were not concerned by this stratification. Finally, the epithelial/mesenchymal classification was consistent with that obtained with the epithelial phenotype gene module.

### Cell line clusters are enriched in somatic mutations

We investigated a common set of 64 genes for the presence of mutations in CCLE and GDSC datasets. However, many inconsistencies between both datasets led us to focus on a set of eight genes (TP53, KRAS, NRAS, APC, PIK3CA, BRAF, PTEN and RB1) for which at least 5% of identical cell lines display mutations in both datasets (Supplementary Information). The mutational profile of cell line clusters was then described based on these genes. Mutation profiles clearly distinguished four clusters (Fig. 2a). The SCLC cluster was enriched in RB1 mutations. The GI cluster was rich in APC and KRAS mutations; NRAS mutations were overrepresented in the HAL cluster and the SKCM cluster was enriched in BRAF mutations. Finally, KRAS mutations were particularly abundant in the EDOT clusters. No significant enrichment in mutations was observed for the GLSR, ADG, BRCA and Mixed 3 cell line clusters (Supplementary Tables 1 and 2). These clusters have fewer mean mutation rates than the other clusters (GDSC: 13% vs. 19%, *t*-test *p*-value = 0.01; CCLE: 17% vs. 22%, *t*-test *p*-value = 0.08).

### Transcriptomic clustering is more consistent than clustering on the basis of tissue of origin in terms of drug responses

The large-scale drug screening programs of the Broad and Sanger Institutes have provided to the scientific community an unprecedented wealth of publicly available data. Molecular data have been systematically collected for each cell line, but far less information is available for drug screening (Supplementary Information). Moreover, in many cases (25% in CCLE and 45% in GDSC) it was not possible to extract the IC_50_ from the dose-response curve. In order to overcome these issues, both study also report the AUC (area under the dose response curve) that can always be calculated.

We evaluated whether our clustering was more discriminant than the tissue of origin of the cell lines, in terms of drug response. We calculated a pseudo *F*-statistic separately for IC_50_ and AUC values for each of the 15 drugs common to CCLE and GDSC. This measurement should capture consistency between the clustering and screening data. It is calculated as the ratio of between-group variance in drug response to the corresponding within-group variance^19^. High pseudo *F* values indicate well-separated, compact clusters. We then compared the pseudo *F* values calculated with our clustering method with those obtained for ‘tissue partitioning’ for a given drug (i.e. each tissue being to correspond to a cluster of cell lines).

**Figure 4:**
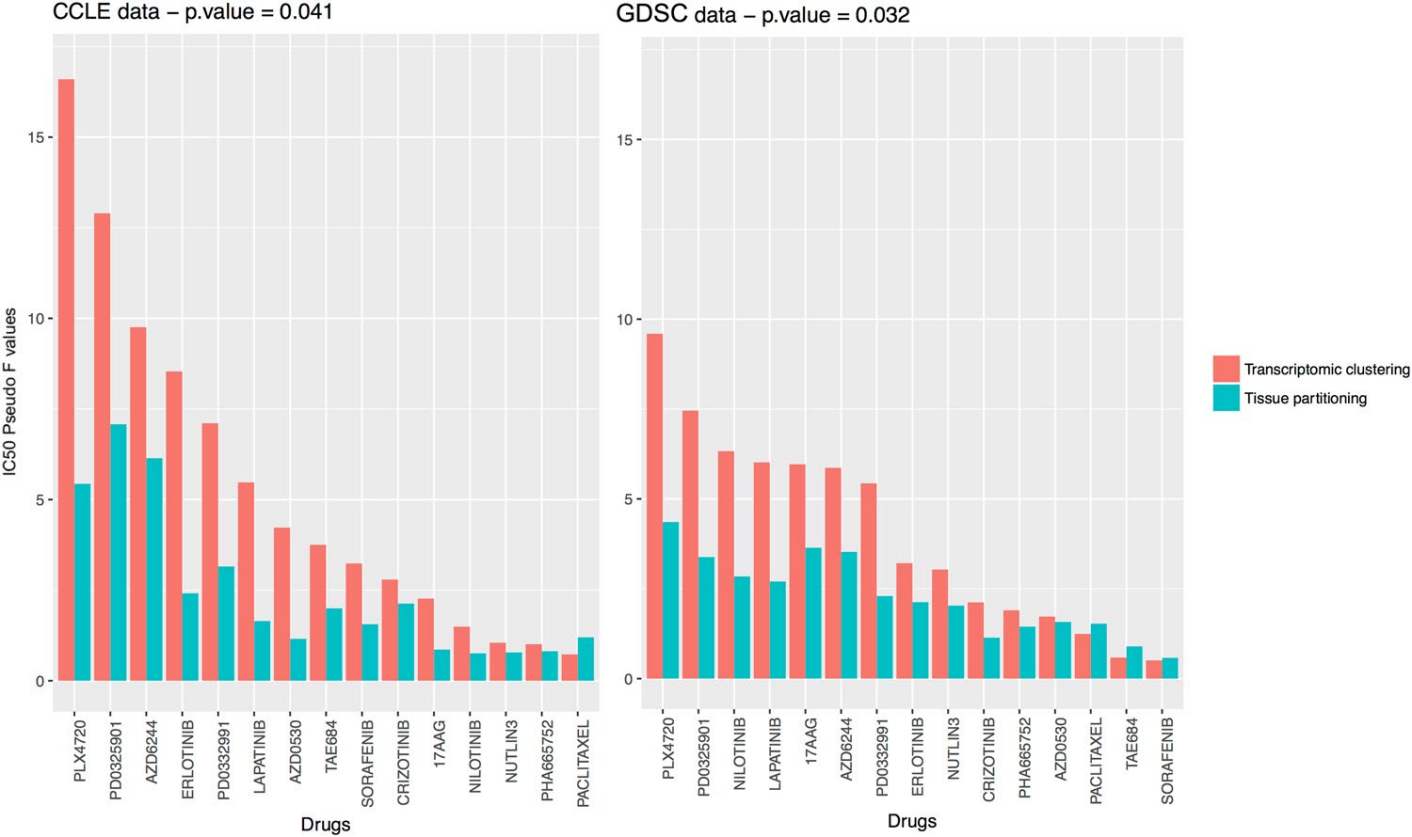
Pseudo *F* value for the 15 drugs common to CCLE and GDSC. The pseudo *F* index have been computed from the IC_50_ values for each drug. The pseudo *F* statistic is the ratio of between-cluster variance to within-cluster variance. Large values of pseudo *F* indicate well-separated, tight clusters. Drugs are listed in descending order of pseudo *F* values for clustering.

Twelve of the fifteen drugs had a higher ratio in CCLE and GDSC for our clustering than for clustering based on tissue of origin with the IC50 (Fig. 4) and ten out of fifteen with the AUC (Supplementary Fig. 4). This trend was confirmed by a *t*-test comparing the pseudo *F* values for our clustering with those for ‘tissue partitioning’ (IC50: CCLE t.test *p*-value = 0.041, GDSC t.test *p*-value = 0.032, AUC: CCLE t.test *p*-value = 0.011, GDSC t.test *p*-value = 0.043). PLX4720 (Raf kinase B inhibitor) and PD0325901 (MEK1 and MEK2 inhibitors) were drugs with the largest pseudo *F* values in both dataset. Paclitaxel was the only molecule in the panel with a higher pseudo *F* value for tissue partitioning in CCLE and GDSC. As the drug sensitivity results were not used to determine the clustering of the cell lines, these findings provide independent evidence for a major role of mRNA levels in drug sensitivity.

### Robust identification of drug response across datasets

**Figure 5:**
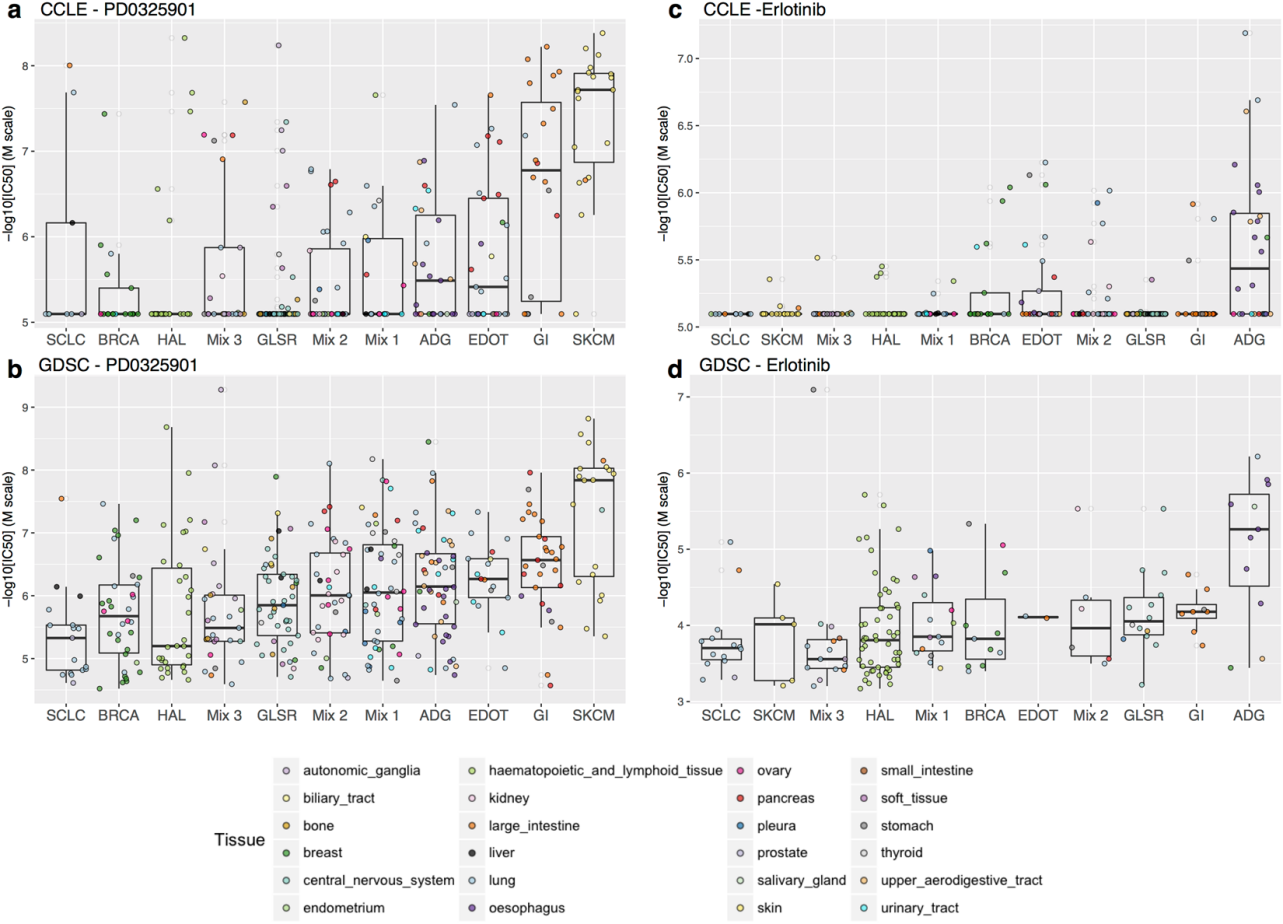
Distribution of IC_50_ values for each in CCLE and GDSC. Ordered according to mean IC_50_ for the cluster. From resistant (left) to sensitive (right).

Subgroups of patients or cell lines defined on the basis of transcriptomic data have been shown to be associated with differences in drug sensitivity^8,9^. We sought to identify associations between clusters of cell lines and “sensitive” or “resistant” drug phenotypes, for the 15 drugs tested in both CCLE and GDSC. For each dataset and each drug separately, we investigated whether the mean IC_50_ of a given cell line cluster differed significantly from those for the other cell line clusters (see Fig. 1 step C and Materials and Methods). Six molecules were found to be significantly associated with six different clusters in both CCLE and GDSC (Table 1 and Supplementary Table 3, Supplementary Fig. 5). The SKCM and GI cell line clusters were both significantly more sensitive than the other cell lines to PD0325901 (MEK 1 and MEK 2 inhibitors) (Fig. 5a). The association of melanoma and PLX4720 (Raf kinase B inhibitor) is already well established and was confirmed by our analysis. Moreover, an inhibitor of MEK 1 and MEK 2, AZD6244, displayed significantly higher levels of activity in cell lines from the SKCM cell line cluster. Both EGFR inhibitors, erlotinib (Fig. 5b) and lapatinib, appeared to be significantly more effective against ADG cell lines than against other cell lines. Hematopoietic and lymphoid tissue cells were sensitive to the CDK4/6 inhibitor PD033991. By contrast, SLCL cell lines appeared to be resistant to lapatinib (EGFR and HER2 inhibitor) and the CDK4/6 inhibitor PD033991 was found inefficient to kill GI cell lines. Finally, AZD6244 (inhibitor of MEK1 and MEK2) appeared ineffective to treat BRCA cell. In addition to variation between drug sensitivity and cell lines, previous studies report variations across the different metrics used to report the drug efficacy^12,20^. We then performed similar analysis using AUC. More than half of the associations between cell lines clusters and drug sensitivity were found still significant with AUC (Table 1 and Supplementary Information).

**Table 1:** Significant associations found between CCLE, GDSC, GSK and GCSI.

CCLE vs GDSC			CCLE vs GDSC			CCLE vs GSK		
IC50			AUC			IC50		
Drug	Cluster	Response	Drug	Cluster	Response	Drug	Cluster	Response
**Erlotinib**	**ADG**	**Sensitive**	**Erlotinib**	**ADG**	**Sensitive**	**Lapatinib**	**Mixed 1**	**Resistant**
**AZD6244**	**SKCM**	**Sensitive**	**AZD6244**	**SKCM**	**Sensitive**	Lapatinib	SKCM	Resistant
**AZD6244**	**BRCA**	**Resistant**	**AZD6244**	**BRCA**	**Resistant**			
Lapatinib	SCLC	Resistant	Lapatinib	HAL	Resistant			
Lapatinib	ADG	Sensitive	Crizotinib	SKCM	Resistant			
PD0332991	GI	Resistant	AZD0530	SKCM	Resistant			
PD0332991	HAL	Sensitive	PLX4720	SKCM	Sensitive			
PLX4720	SKCM	Sensitive						
PD0325901	GI	Sensitive						
**PD0325901**	**SKCM**	**Sensitive**						
**CCLE vs gCSI**			**GDSC vs gCSI**					
**IC50**			**IC50**					
**Drug**	**Cluster**	**Response**	**Drug**	**Cluster**	**Response**			
**Erlotinib**	**ADG**	**Sensitive**	**PD0325901**	**SKCM**	Sensitive*			
**Erlotinib**	**Mixed 1**	**Resistant**						
**Erlotinib**	**GLSR**	**Resistant**						
**Erlotinib**	**SKCM**	**Resistant**						
**Lapatinib**	**Mixed 1**	**Resistant**						
Lapatinib	ADG	Sensitive						
PD0325901	BRCA	Resistant						
**PD0325901**	**SKCM**	**Sensitive**						
**CCLE vs gCSI**			**GDSC vs gCSI**					
**Mean Viability**			**Mean Viability**					
**Drug**	**Cluster**	**Response**	**Drug**	**Cluster**	**Response**			
**Erlotinib**	**ADG**	**Sensitive**	**PD0325901**	**SKCM**	**Sensitive**			
**Erlotinib**	**Mixed 1**	**Resistant**						
**Erlotinib**	**GLSR**	**Resistant**						
**Erlotinib**	**SKCM**	**Resistant**						
Erlotinib	HAL	Resistant						
Erlotinib	SCLC	Resistant						
PD0325901	BRCA	Resistant						
**PD0325901**	**SKCM**	**Sensitive**						

In bold associations found significant in at least three datasets. The association between PD0325901 and SKCM had an adjusted p-values of 0.058 (marked with*).

We further evaluated the relevance of our clustering regarding the drug sensitivity using two external public datasets. We first study the 118 cell lines tested in common between the CCLE and the GlaxoSmithKline cell line collection (GSK)^21^ on lapatinib and paclitaxel (GDSC was excluded due to small sample size, see Supplementary Information). We found that lapatinib was significantly inactive to kill cells from clusters SKCM and Mixed 1 in both CCLE and GSK (Table 1). Since the set of common cell lines and drugs was small between CCLE, GDSC and GSK (Supplementary Table 4), we consider the Genentech Cell Line Screening Initiative (gCSI)^22^. A panel of 244 unique cell lines and 5 drugs overlap between CCLE, GDSC and gCSI. Instead of AUC, the gCSI reported the mean viability statistic to measure drug efficacy in addition to the IC_50_. Eight associations between cell lines clusters and drug sensitivity were found significant using the IC_50_ and nine with the mean viability statistic. Among them, the sensitivity of ADG to erlotinib and lapatinib as well as the efficacy of PD0325901 to kill cells from SKCM cluster were common to CCLE, GDSC and gCSI (Table 1 and Supplementary Information). Our results suggest that our cell line clustering is able to find significant associations with drugs efficacy robustly in four different dataset, despite the large variations across pharmacological data and drug response measures.

### Distinct drug profiles were associated with the various cell line clusters

We applied the same procedure to all the drugs tested in the CCLE (24 molecules) and GDSC (129 molecules) studies. For each dataset and each of the 153 drugs separately, we determined whether the mean IC_50_ of a given cell line cluster was significantly different those of the other cell line clusters (Supplementary Tables 5 and 6). Overall, the most striking result was the very small number of drugs associated with a sensitive profile (88 associations, including 71 unique drugs) compared to drugs associated with a resistant profile (163 associations, including 92 unique drugs) (Supplementary Information). It was particularly interesting to observed that Mixed 2 and Mixed 3 clusters were each sensitive to only one drug: respectively midostaurin and vorinostat. Both drugs are targeted agents (PI3K/mTOR inhibitor and HDAC inhibitor). These clusters are made of several cells from different tissue of origin. However, we were able to identify targeted therapies active to kill those cells. These results provide further evidences that our clustering can identify relevant groups of cell sharing unknown features associated to targeted drugs.

Overall, these results suggest that cancer cell lines can be classified, on the basis of their transcriptomic profile, into 11 clusters that may or may not be specific to the tissue of origin. We demonstrated that transcriptomic clustering was more consistent than clustering on the basis of tissue of origin in terms of drug response whatever the drug sensitivity metric considered. We were also able to find several significant associations between clusters of cell lines and “sensitive” or “resistant” drug phenotypes. Many of these associations were robustly found across four different datasets with three different drug response metrics. As the drug sensitivity results were not used to determine the clustering of the cell lines, these findings provide independent evidence about the relevance of this new classification. Furthermore, we show that when we are trying to associate a group of genes from a consistent biological pathway with a group of cell lines, rather than a single gene with a single drug, robust associations can be established across several pharmacologic datasets.

## Discussion

Despite the progress in the development of *in vivo* models, cancer cell lines remain a key tool in cancer research. Patients are usually treated with combination therapy. However, it is important to better understand the mechanisms involved with monotherapies before moving forward to study combination therapies. Here, we introduce a new cell line classification constructed from 471 cell lines derived from tumors from 24 different tissues. A biological network analysis for the most variant genes identified 11 clusters of cell lines. These clusters appeared robust in two large-scale cell line panels. This biologically driven gene selection process, which is probably less sensitive to sample fluctuations than other methods, made it possible to capture strong biological signals that might be concealed by the noise present in microarray data. Several studies have reported that the incorporation of network information improves the stability of gene selection and the biological interpretability of biomarker signatures for a given prediction accuracy^23–25^.

In this new classification, a clear distinction was established between non-epithelial cancer cell lines (GLSR, SKCM, Mixed 3) and epithelial cell lines (EDOT, BRCA, GI). This suggests that EMT-associated differences in gene expression are major determinants of the gene expression–based stratification of cancer cell lines. This new molecular clustering system classified more than 65% of the cell lines differently from the currently used tissue-of-origin cell line classification system. Only four clusters consisted mostly of cell lines originating from a single tissue. Furthermore, three clusters include cells with expression profiles stronger than that of the original tissue (Mixed clusters). Thus, 25% of the cells lines displayed no link to any tissue of origin or related organ system.

One of the most interesting cases was the triple-negative breast cancer (TNBC). We focused on this subtype, as it is the only subtype of breast cancer without any targeted therapy associated. TNBC were found to be highly heterogeneous, falling into six different clusters. This divergence shows the relevance of studying cell lines from various tumor types. Drug response was dependent on cluster membership, with the EDOT cluster sensitive to chemotherapy, whereas the BRCA cluster was resistant. The widely dispersed TNBC cell lines were mostly mesenchymal, whereas the cell lines of the BRCA cluster were exclusively epithelial. TNBC is increasingly emerging as a heterogeneous disease^26,27^, with tumors differing in histological features, gene expression profiles, clinical behavior, overall prognosis^28^ and sensitivity to systemic treatment^9,29,30^. These findings provide strong evidence to suggest that TNBC heterogeneity is reflected at the cell line level. Our results suggest also that particular attention should be paid to the selection of cell lines for studies of particular types or subtypes of cancer.

By analyzing several large-scale public data sets, we demonstrated that drug efficacy is significantly associated to transcriptomic profile. A comparative analysis recently showed that the gene-expression profiles of the 471 cell lines shared by CCLE and GDSC were highly concordant whereas the reported cell-line drug sensitivities for the 15 drugs tested in both studies were highly inconsistent^12^. The authors put forward several hypotheses to explain these discrepancies, including differences in experimental protocols, the viability assay and procedures for summarizing dose response and non-observed IC_50_ (the half maximal inhibitory concentration). Despite discrepancies between the drug sensitivity data retrieved from different databases, we were able to find some robust combinations. Well-known drug associations were found, such as the sensitivity of SKCM lines to vemurafenib. We also found that cancers with BRAF mutations, such as melanoma^31^ and cancers with KRAS/BRAF mutations, such as colorectal cancer^32^, were more sensitive to MEK inhibitors. Furthermore, CDK4/6 inhibition-induced cell death has been noted in cell lines and xenografts derived from patients with T-cell leukemia^33^. SCLC cell lines have been shown to be resistant to lapatinib, but combination with a cytotoxic agent may yield promising results^34^.

The decline in the number of new treatments approved in recent years is a major challenge for the pharmaceutical industry. One of the reasons for this decline is the lack of systematic evaluation of therapeutic indications for a drug that is either in advanced development phase or has already obtained a marketing authorization. The so-called “drug repositioning process” proposed to find new therapeutic indications to already approved drugs with faster development times and reduced risks. Furthermore, it allows patients to have access to earlier therapeutic advances^35^. Several robust associations were found. Targeted drugs were found efficient to treat clusters of cell lines constituted of cell from different tissues. These drugs are known to be active in one or several tissues that constitute theses clusters. It would be of particular interest to test specifically these drugs on the other tissues represented in these cell lines clusters. For example, cluster ADG is mostly constituted of upper-aerodigestive, oesophagus and urinary tract cancer cell lines. ADG cluster was particularly sensitivity to the anti EGFR - erlotinib. If EGFR is a validated target for upper-aerodigestive cancer^36–38^ the therapeutic potential of erlotinib has already been highlighted for bladder cancer^39^ and showed promising results in phase II for oesophagus cancer^40,41^.

Different types of drugs have been used in the panels. Around 10% of the 153 drugs screened in CCLE and GDSC, and only 1 out of the 15 drugs in common to both studies, are cytotoxic agents. These drugs are expected to be broadly active among the cell line panel since they are not specific molecules. On the contrary, targeted agents are expected to be active only in a subset of cell lines, at least, those carrying the given target. Furthermore, the recent study published by Rees *et al*.^42^ demonstrated that target’s expression and drug sensitivity were correlated in only 31% of the cases. Grouping cell lines on the basis of their transcriptomic profiles makes it possible to identify subsets of cells with common off-target features. It is then more relevant to compare the drug sensitivity between cell lines of these groups rather than examined the correlation of response of each cell line to a particular drug reported by one dataset with the response of the same cell line to the same drug reported by another dataset. These results suggest that when robust clusters of cell lines based on biologically network-driven approach are considered, consistency between drug responses can be achieved.

In conclusion, our cell line classification provides novel insight for pharmacogenomics studies. As cell lines remain the most widely used models for the preclinical evaluation of candidate cancer drugs, further investigation should be made to use this classification in the development of cancer treatments with the aim of reducing the attrition rate.

## Materials and Methods

### Pharmacogenomics data

We collected data from the Broad and Sanger Institutes. The CCLE profiled 24 anticancer drugs on 1,036 cell lines. The GDSC screened 138 drugs on 727 cell lines. Both datasets contain genome-wide gene expression and massive parallel sequencing data. All data were recovered, curated and annotated with the pipeline developed by Haibe-Kains *et al*.^12^ (the GDSC was referred to the Cancer Genome Project [CGP] in Haibe-Kains *et al*.). We used this pipeline as described in the original article, but with a different method for the normalization of gene expression. Haibe-Kains *et al*. normalized gene expression data by frozen robust multiarray analysis, fRMA^43^. This method was designed to combine several datasets and overcome multiple batch issues. This strategy is relevant when trying to ensure assay reproducibility. Even though this approach would be unlikely to have a major effect on gene expression values, we chose to normalize the gene expression data separately with RMA^44^, to ensure that the two datasets were perfectly independent. Our analysis focused on 471 cell lines and 15 drugs for which we have transcriptomic and drug sensitivity data available in both the CCLE and GDSC studies. We also collected two large datasets to validate our classification. Data from the GlaxoSmithKline cell line collection were retrieved from Haibe-Kains *et al*.^12^. The Genentech Cell Line Screening Initiative data were available from compareDrugScreens R package published by Haverty *et al*.^22^.

### Gene expression data

Transcriptomic data were restricted to the 12,153 genes common to the two technologies used by GDSC and CCLE (Affymetrix GeneChipHG-U133A and HG-U133PLUS2, respectively). The Jetset method^45^ was used to select a unique probe set for given genes. The same probe set was used in both datasets for 83% of the genes.

### Drug sensitivity data

The micromolar concentration (*μM*) at which the drug inhibited 50% of maximal cell growth was used to assess drug sensitivity as well as the area under the dose response curve (AUC). We also consider the mean viability statistic when comparing with gCSI. These measurements were converted to a common scale (−*log*10(*M*) for IC50, [0,1] for AUC and 1 – mean viability for mean viability), such that high values would be correspond to cell lines sensitive to drugs.

### Gene selection by the inflexion point method

We selected the most variant genes, based on the inflexion point of the interquartile range (IQR) distribution for gene expression. This method is more data-driven than a fixed threshold for defining the proportion of genes displaying the highest level of variation. The full procedure is described below. For each gene, we: (1) calculated the IQR for all cell lines, (2) sorted the IQR values of the genes in ascending order, to generate an ordered distribution, (3) estimated the major inflection point of the IQR curve as the point on the curve furthest away from a line drawn between the start and end points of the distribution, and (4) retained genes with an IQR higher than the inflection point.

### Gene expression-based identification of cell line clusters

We developed a biological network-driven process based on transcriptomic data, to identify robust clusters of genes and cell lines. This process can be broken down into two parts: (A) identification of robust clusters of genes, used for (B) identification of robust clusters of cell lines.

(A) The gene selection process is a three-step procedure. (1) We selected the most variant genes from among the 12,153 genes common to GDSC and CCLE, by the inflexion point method. (2) We performed hierarchical consensus clustering (ConsensusClusterPlus R Package) to identify robust gene modules. The consensus-clustering step, based on Pearson distance and Ward linkage, identified robust clusters of genes. It involved hierarchical clustering by resampling (1,000 iterations) randomly selected genes. (3) We identified known biological networks, for each gene cluster separately, using String© database software version 9.1 (http://string-db.org/). We then applied a two-step selection process: (1) we selected strong biological networks by retaining only genes for which connection scores of at least 0.7 were obtained with String© database software, (2) within each biological network, we selected groups of genes for which expression levels were correlated, with a correlation coefficient of at least 0.5. We used the R package clusterProfiler^46^ for comparing and visualizing gene ontologies profiles among gene modules. (B) We applied a consensus-clustering with hierarchical clustering to the cell line gene expression profiles, using the selected genes to visualize the optimal number of stable cell line clusters.

### Characterization of cell line clusters at the transcriptomic and mutational levels

Gene set enrichment analysis (GSEA) was performed on genes modules built in step (A) of the biological network-driven process described above. We identified up-regulated or down-regulated gene modules, associated with each cell line cluster. An analysis was first performed to identify genes differentially expressed between a particular cluster and all the other cell lines, based on a linear model. For a given cluster *k*, cell lines were partitioned into two groups *j* = {*Cluster-k, non-Cluster-k*}. We then performed a differential analysis by comparing the mean gene expression of each group in a linear model (limma R package^47^). The analysis was performed separately for each dataset. The results were used to rank genes in order of significance and to search for overrepresented gene modules, by pre-ranked gene set enrichment analysis (GSEA).

Genes with significantly higher frequencies of mutation in a given cluster were identified by one-tailed Fisher’s exact tests. We compared the occurrence of any given mutation in each cell line clusters with that in all the remaining clusters combined.

### Identification of cell line clusters common to different studies

We studied the likeness between the clusterings for CCLE and GDSC, by clustering the cell lines with a similarity matrix (hierarchical clustering with Pearson’s metric and the Ward agglomerative method). The similarity matrix contains the number of times two cell lines are clustered together in each dataset (0 = never, 1 = only in one classification, 2 = in both classifications). This similarity matrix constitutes a natural visualization tool for assessing the consistency between two clustering patterns. In particular, if we associate a color gradient to the 0–2 range of real numbers, such that white corresponds to 0, and dark blue corresponds to 2, and if we assume that the matrix is arranged so that items belonging to the same cluster are adjacent to each other (with the same item order used to index both the rows and the columns of the matrix), a matrix corresponding to a perfect consensus will be displayed as a color-coded heatmap characterized by blue blocks along the diagonal, on a white background. The accuracy was calculated as the number of times two cell lines clustered together divided by the number of possible combinations

### EMT cell line classification

The “epithelial” or “mesenchymal” status of each cell line was defined with the signature identified by Taube *et al*.^18^. This epithelial-to-mesenchymal transition signature consists of 159 downregulated genes and 90 upregulated genes. We performed a hierarchical clustering of cell lines based on these 249 genes and labeled clusters of cell lines according to the overexpression of known epithelial marker genes, known mesenchymal marker genes or neither.

### Definition of breast cancer subtypes

Breast cancer subtypes were defined with a bimodal mixture of two Gaussian distributions for ESR1, PGR and ERBB2 gene expression. Triple-negative (TN) breast cancer cell lines were defined by an absence of estrogen and progesterone receptor expression and a lack of ERBB2 overexpression/amplification (*n* = 31). We subsequently defined breast cancer cell lines overexpressing ESR1 but with a lower level of ERBB2 expression as the ER + Her2- subtype (*n* = 7), with cell lines overexpressing the ERBB2 gene defined as the Her2 + subtype (*n* = 7).

### Impact of cell line clustering on drug sensitivity

We investigated the relevance of our clustering for drug sensitivity, by comparing the results obtained for this method with those for ‘tissue partitioning’ (i.e. each tissue of origin being considered to correspond to a cluster of cell lines). We calculated the pseudo *F* index computed from any drug sensitivity statistic (IC_50_, AUC, mean viability) for each drug. The pseudo *F* statistic is the ratio of between-cluster variance to within-cluster variance^19^. It is defined as *[Between-cluster variance*/*(N-K)]*/*[Within-cluster variance*/*(K-1)]*, where *N* is the number of observations (*N* = 471) and *K* is the number of clusters (*K* = 11 or *K* = 24). Large values of pseudo *F* indicate well-separated, tight clusters.

The sensitivity and resistant phenotypes of each cell line for a given drug were defined by comparing the drug sensitivity measure between cell lines from any given cluster and the cell lines in all remaining clusters combined. We focus on IC_50_ for clarity. For a given cluster *k*, cell lines were partitioned into two groups *j* = {*Cluster-k, non-Cluster-k*}. We then compared the mean IC_50_ values of the two groups in a *t* test. The sign of the statistical test was used to define the phenotype as sensitive (*t* > 0) or resistant (*t* < 0). We accounted for multiple testing, by calculating the FDR-adjusted *p*-value for each drug. An FDR-adjusted *p*-value < 0.05 was considered significant.

#### Supplementary data

A summary of each cell line cluster, with information regarding tissue composition, molecular profile and drug profile, and other supplementary data for this article can be accessed from the publisher’s website.

#### Consent for publication

All authors read and approved the final manuscript.

## Electronic supplementary material

Supplementary Information

## Data Availability

All data analyzed during this study were retrieved and curated based on the pipeline published by Haibe-Kains *et al*.^12^. Data are available from the CCLE website (http://www.broadinstitute) and GDSC website (http://www.cancerrxgene.org/downloads/).
