# Author Correction: New insight for pharmacogenomics studies from the transcriptional analysis of two large-scale cancer cell line panels

**DOI:** 10.1038/s41598-018-36812-3

**Published:** 2018-12-13

**Authors:** Benjamin Sadacca, Anne-Sophie Hamy, Cécile Laurent, Pierre Gestraud, Hélène Bonsang-Kitzis, Alice Pinheiro, Judith Abecassis, Pierre Neuvial, Fabien Reyal

**Affiliations:** 1 Residual Tumor & Response to Treatment Laboratory (RT2Lab), PSL Research University, Translational Research Department, F-75248 Paris, France; 2 U932 Immunity and Cancer; INSERM; Institut Curie, Paris, France; 3 Laboratoire de Mathématiques et Modélisation d’Evry, Université d’Évry Val d’Essonne, UMR CNRS 8071, ENSIIE, USC INRA, Evry, Val d’Essonne France; 4 Mines Paristech, PSL-Research University, CBIO-Centre for Computational Biology, Mines ParisTech, Fontainebleau, F-77300 France; 5 Institut Curie, PSL Research University, Mines Paris Tech, Bioinformatics and Computational Systems Biology of Cancer, INSERM U900, F-75005 Paris, France; 6 Department of Surgery, Institut Curie, Paris, F-75248 France; 7 Institut de Mathématiques de Toulouse; UMR5219 Université de Toulouse; CNRS UPS IMT, F-31062 Toulouse Cedex 9, France

Correction to: *Scientific Reports* 10.1038/s41598-017-14770-6, published online 09 November 2017

The original version of this Article contained an error in the spelling of the author Anne-Sophie Hamy, which was incorrectly given as Anne-Sophie Hamy-Petit.

In addition, the Supplementary Information file originally published with this Article contained an error in the title, where

“pharmacogenetics” now reads “pharmacogenomics”.

These errors have now been corrected in the HTML and PDF versions of the Article, and in the accompanying Supplementary Information file.

