# Supplementary material for: New insight for pharmacogenomics studies from the transcriptional analysis of two large-scale cancer cell line panels
Source: Sci Rep. 2017 Nov 9;7:15126. doi: 10.1038/s41598-017-14770-6 (PMC5680301; doi:10.1038/s41598-017-14770-6)
Supplement: Supplementary file 1 — Supplementary Information [file 41598_2017_14770_MOESM1_ESM.pdf]

New insight for pharmacogenomics studies from the transcriptional analysis of two large-scale cancer cell line panels

Benjamin Sadacca[1,2,3] , Anne-Sophie Hamy[1,2], Cécile. Laurent [1,2] , Pierre Gestraud[6], Hélène Bonsang-Kitzis[1,2,7], Alice Pinheiro[1,2], Judith Abecassis[1,2,5,6], Pierre Neuvial[3,8], Fabien Reyal*[1,2,7]

[1] Residual Tumor & Response to Treatment Laboratory (RT2Lab), PSL Research University, Translational Research Department, F-75248, Paris, France.

[2] U932 Immunity and Cancer; INSERM; Institut Curie; Paris, France.

[3] Laboratoire de Mathématiques et Modélisation d'Evry, Université d'Évry Val d'Essonne, UMR CNRS 8071, ENSIIE, USC INRA.

[4] Department of Tumor Biology, Institut Curie, Paris, F-75248, France.

[5] Mines Paristech, PSL-Research University, CBIO-Centre for Computational Biology, Mines ParisTech, Fontainebleau, F-77300 France.

[6] Institut Curie, PSL Research University, Mines Paris Tech, Bioinformatics and Computational Systems Biology of Cancer, INSERM U900, F-75005, Paris, France

[7] Department of Surgery, Institut Curie, Paris, F-75248, France.

[8] Institut de Mathématiques de Toulouse ; UMR5219 Université de Toulouse; CNRS UPS IMT, F-31062 Toulouse Cedex 9, France.

Supplementary Figures legends

Supplementary Fig. 1: Gene ontology analysis for gene clusters identified in CCLE and GDSC.

Supplementary Fig. 2: (A) Heatmap clustering with 471 cell lines (in columns) and selected genes (in rows), with GDSC data. (B) EMT status of the cell lines.

Supplementary Fig. 3: GSEA enrichment results. Green indicates gene modules significantly downregulated, whereas red indicates gene modules significantly upregulated. Results for CCLE are displayed in the left column; results for GDSC are displayed in the right column.

Supplementary Fig. 4: Pseudo *F* value for the 15 drugs common to CCLE and GDSC. The pseudo *F* index have been computed from the AUC values for each drug. The pseudo *F* statistic is the ratio of between-cluster variance to within-cluster variance. Large values of pseudo *F* indicate well-separated, tight clusters. Drugs are listed in descending order of pseudo *F* values for clustering

Supplementary Fig. 5: Distribution of IC_{50} values for each cluster in CCLE and GDSC. Ordered according to mean IC_{50} for the cluster. From resistant (left) to sensitive (right).

Supplementary Fig. 6: Barplot of the number of mutations in cell lines
The eight genes in bold were used for cluster characterization
(A) Proportion of cell lines mutated for each gene. Red in CCLE and blue in GDSC.
(B) Proportion of same cell lines mutated in CCLE and GDSC.
(C) Among the mutated cell lines in CCLE and GDSC, proportion of same cell lines mutated.
(D) Among the mutated cell lines in CCLE and GDSC, proportion of cell lines with exactly the same mutation.

Supplementary Fig. 7: Number of drugs tested for each cell line. Many drugs were not tested for a large set of cell lines (in red). Even when a test was performed, in many cases IC50 could not be extracted and have been estimated as explained in the main text (green). Values in blue are observed IC_{50} values.

Supplementary Fig. 8: AUC distribution for each cluster in CCLE and GDSC. Ordered according to the mean AUC. From resistant (left) to sensitive (right).

Supplementary Fig. 9: IC_{50} distribution for each cluster in CCLE and GSK. Ordered according to the mean IC_{50}. From resistant (left) to sensitive (right).

Supplementary Fig. 10: IC_{50} distribution for each cluster in GDSC and GSK. Ordered according to the mean IC_{50}. From resistant (left) to sensitive (right).

Supplementary Fig. 11: IC_{50} distribution for each cluster in CCLE and gCSI. Ordered according to the mean IC_{50}. From resistant (left) to sensitive (right).

Supplementary Fig. 12: IC_{50} distribution for each cluster in GDSC and gCSI. Ordered according to the mean IC_{50}. From resistant (left) to sensitive (right).

Supplementary Fig. 13: Mean viability distribution for each cluster in CCLE and gCSI. Ordered according to the average mean viability. From resistant (left) to sensitive (right).

Supplementary Fig. 14: Mean viability distribution for each cluster in GDSC and gCSI. Ordered according to the average mean viability. From resistant (left) to sensitive (right).

Supplementary Fig. 15: Drugs displaying significantly high (A) or low (B) levels of activity in a given pair of cell lines (i.e. in CCLE and GDSC) relative to the remaining cell lines. The 138 drugs from CCLE and GDSC were used. Colors correspond to the drug target family. (C) Distribution, by target family, of the 138 drugs.
(D) The drugs significant for each cell line cluster. Drugs and clusters are ordered by number of associations.

<u>Supplementary tables legends</u>

Supplementary Table 1: Mutations for each cluster in CCLE dataset. The proportion of cell lines mutated, with raw and adjusted Fisher *p*-values.

Supplementary Table 2: Mutations for each cluster in GDSC dataset. The proportion of cell lines mutated, with raw and adjusted Fisher *p*-values.

Supplementary Table 3: Significant associations between CCLE and GDSC found in both dataset with IC_{50}.

Supplementary Table 4: Number of cell lines with sensitivity measure in each dataset. The numbers were computed based on the cell lines common to each pair of dataset.

Supplementary Table 5: List of drugs significantly associated with a sensitivity phenotype for each cluster.

Supplementary Table 6: List of drugs significantly associated with a resistant phenotype for each cluster.

Supplementary Table 7: Significant associations between CCLE and GDSC found in both dataset with AUC.

Supplementary Table 8: Significant associations between CCLE and GSK found in both dataset with IC_{50}.

Supplementary Table 9: Significant associations between CCLE and gCSI found in both dataset with IC_{50}.

Supplementary Table 10: Significant associations between CCLE and gCSI found in both dataset with mean viability.

Supplementary Information

Discrepancies in mutational data between CCLE and GDSC

We investigated a set of 64 genes for the presence of mutations in both the CCLE and GDSC datasets. We investigated the consistency of mutation patterns. Thirteen genes were reported to have no mutations in GDSC, in any of the 471 cell lines studied (AKT2, CCND1, CCND2, CCND3, CDK4, CDK6, EP300, FGFR2, MDM2, MLLT3, MYCL1, MYCN and SMARCB1). We focused on the remaining 51 genes. The most frequently mutated gene was TP53, with a frequency over 60%, mutation frequencies remaining below 20% for the other genes (Supplementary Fig. 6A). A previous study reported high levels of agreement concerning the reported presence of mutations in identical cell lines[1]. However, for cell lines displaying mutations of at least one of these genes, the mean proportion of cell lines with mutations of the same genes was about 20% (Supplementary Fig. 6C) and the mean proportion of cell lines displaying identical mutations within the genes was 44% (Supplementary Fig. 6D). Only for eight genes (TP53, KRAS, NRAS, APC, PIK3CA, BRAF, PTEN and RB1) did at least 5% of identical cell lines display mutations in both datasets (Supplementary Fig. 6B). Furthermore, the proportion of identical cell lines mutated was 64%, and the mutations observed were identical in 84% of cases. By contrast, 12% of identical cell lines had mutations for the remaining genes, with 36% of the mutations observed identical (Supplementary Fig. 6C-D).

Drug screening data

The large-scale drug screening programs of the Broad and Sanger Institutes have provided to the scientific community an unprecedented wealth of publicly available data. Molecular data have been systematically collected for each cell line, but far less information is available for

drug screening. Considering the 471 cell lines common to CCLE and GDSC, only 47% of the cell lines from CCLE and 90% of those from GDSC have been tested for at least one drug (Supplementary Fig. 7). In CCLE and GDSC, 28% and 22% of the cell lines, respectively, were tested for all compounds. Moreover, in many cases (25% in CCLE and 45% in GDSC) it was not possible to extract the IC_{50} from the dose-response curve. In these cases, the IC_{50} was set to the maximum concentration used for screening in the CCLE study, whereas a mathematical extrapolation was applied in the GDSC study. In order to overcome these issues, both study also report the AUC (area under the dose response curve) that can always be calculated.

Comparison between CCLE and GDSC based on AUC

To evaluate the relevance of our method to the variation of drug sensitivity metric, we looked at the area under the dose response curve (AUC). We evaluate the robustness of our cell line classification with the 15 drugs shared between CCLE and GDSC.

We first evaluated whether our clustering was more discriminant than the tissue of origin of the cell lines, in terms of drug response. We calculated a pseudo F-statistic using the AUC values for each of the 15 drugs common to CCLE and GDSC. Ten out of fifteen drugs had a higher ratio in CCLE and GDSC for our clustering than for clustering based on tissue source (Supplementary Fig. 4). This trend was confirmed by a t-test comparing the pseudo F values for our clustering with those for 'tissue partitioning' (CCLE t.test p-value = 0.011, GDSC t.test p-value=0.043).

We then performed the same analyses as those performed with the IC_{50}, to identify association between cell line clusters and drug response but based on the AUC. Four out of

the seven cell lines clusters-drugs associations were robustly found using the IC_{50} and the AUC (Supplementary Table 7 and Fig. 8): ADG cluster being sensitive to erlotinib, SKCM being sensitive to the Raf kinase B inhibitor PLX4720 and the MEK inhibitor AZD6244 whereas the latter is not active in BRCA cell line cluster. In addition, SKCM cluster appears resistant to crizotinib (ALK inhibitor) and AZD0530 (Src and Abl inhibitor) that is concordant with the wild-type mutational status of SCKM cells for ALK, Abl1/2 and Src[2] . Lapatinib appears inefficient in HAL cluster that is consistent as no EGFR or ERBB2 mutation have been reported in HAL cell lines in both CCLE and GDSC.

Many cell lines never achieved 50% inhibition for many drugs. In this case, data have been either truncated by the maximum dose tested (as in CCLE), either they have been extrapolated (as in GDSC) (Supplementary Figure 5). On the contrary the AUC can always be calculated. These variabilities can explain the variation of results. However more than half of the cell lines clusters-drug associations were found using both IC50 and AUC.

<u>Comparison between CCLE, GDSC and GSK</u>

We explored the relevance of our classification with two outside datasets. For this, we kept our robust clustering of cell lines define with the CCLE and GDSC. For each dataset and each drug separately, we investigated whether the mean drug sensitivity measure available of a given cell line cluster differed significantly from those for the other cell line clusters (see Materials and Methods). We then investigate if significant cell line clusters-drug associations can be found in these different datasets.

First we consider the third dataset used by Haibe-Kains et al[1], the GlaxoSmithKline cell line collection (GSK)[3]. This panel is composed of 319 cell lines that have been screened on 19 drugs. Only lapatinib and paclitaxel have been tested by GSK among the 15 drugs shared between CCLE and GDSC. A set of 194 cell lines was common to the three studies. However

the actual number of cell lines for which we have a sensitivity measure for lapatinib and paclitaxel is smaller especially in GDSC. Supplementary Table 4 shows the number of cell lines with a sensitivity measure for each drug in each dataset among the 194 cell lines shared between CCLE, GDSC and GSK. Only 70 cell lines have a sensitivity measure for paclitaxel and lapatinib in GDSC. Moreover, the number of cluster with less than 3 cell lines goes up to 4 for paclitaxel and 5 for lapatinib. Given the too small number of cell lines available, we therefore compared only the results between CCLE and GSK. Comparisons were based on IC_{50}, the unique measure available in GSK. Two associations were found in both CCLE and GSK suggesting that lapatinib is inactive in SKCM and in Mixed 1 clusters (Supplementary Table 8, Supplementary Fig. 9 and 10). Prickett et al[4] found some evidence that lapatinib may be more active in melanoma cell lines with ErbB4 mutations than wild-type melanoma cells. However, no ErbB4 mutations have been called in melanoma cells from cluster SKCM[2].

Comparison between CCLE, GDSC and gCSI

Since the set of common cell lines and drugs was small between CCLE, GDSC and GSK (Supplementary Table 4), we repeated the analysis with the panel introduced by the Genentech Cell Line Screening Initiative (gCSI)[5]. Data were recovered from the *R* package *compareDrugScreens.* A panel of 244 unique cell lines and 5 drugs overlap between CCLE, GDSC and gCSI. Once again the overlap of cell lines between GDSC and gCSI is relatively small (Supplementary Table 4). Drug sensitivity measure was available for less than 3 cell lines in 4 clusters for erlotinib and lapatinib; in 3 cell line clusters for crizotinib and paclitaxel. For these reasons, we focused only on PD0325901 when comparing GDSC and gCSI. Two measures of sensitivity are available in *compareDrugScreens*. In addition to the classical IC_{50}, they introduced the mean viability statistic that is the arithmetic average of the fitted viabilities at each tested dose. This metric is closely related to the AUC. We used the

mean viability to evaluate the robustness of our cell line classification to the drug sensitivity metric variation.

Height associations were found significant in both CCLE and gCSI using the IC_{50} (Supplementary Table 9, Supplementary Fig. 11 and 12). Among them three were also found when comparing CCLE to GDSC: ADG cluster sensitive to erlotinib and lapatinib; PD0325901 actives in SKCM. In addition, the resistance of Mixed 1 cluster to lapatinib found when comparing CCLE to GSK appeared once again significant here. Additional associations were found specifically. Erlotinib were inactive to treat cells from Mixed 1, GLSR and SKCM. BCRA cluster was found resistant to PD0325901. Regarding the comparison with GDSC, a strong trend could be observed between SKCM and PD0325901 (gCSI Effect = 0.39, adjusted p-value =3.05E-10, GDSC Effect = 0.13, adjusted p-value =0.058).

Considering the mean viability statistic (Supplementary Table 10, Supplementary Fig. 13 and 14), 2 associations among those identified when comparing CCLE to GDSC were found again: ADG cluster sensitive to erlotinib and SKCM sensitive to PD0325901. In addition, 8 more clusters were found resistant to erlotinib: SCLC, Mixed 1, HAL, GLSR and SKCM. These findings were supported by several phase II studies reporting the inactivity of erlotinib in patients with gliomas[6,7] and melanomas[8] as well as the lack of EGFR mutation in small-cell lung cancers[9]. BRCA appeared resistant to the MEK inhibitor PD0325901. It has been shown that PD0325901 is more active in basal-like breast cancer lines than in luminal and Her2+ lines[10]. Indeed, only 3 out of the 10 breast cancer cell lines from BRCA cluster that have been tested for PD0325901 are TNBC. For GDSC, similar results than those found with the IC_{50} were found regarding the sensitivity of SKCM to PD0325901.

Distinct drug profiles were associated with the various cell line clusters

The GI cell line cluster was sensitive to drugs targeting the ERK signaling pathway (MEK1/2, Hsp90): 17AAG, AZD6244, PD0325901 (Supplementary Fig. 15). The targeting of this pathway was also found to be effective against the cell lines of the ADG cluster, but only for drugs targeting EGFR and ERBB2. Furthermore, these cell lines seems to be particularly sensitive to drugs acting on the cytoskeleton. SKCM cell lines were sensitive to drugs inhibiting the ERK signaling pathway, regardless of the protein from this pathway targeted (BRAF, MEK1/2, FLT3, JAK2, NTRK1, RET). Hematopoietic cells were sensitive to molecules targeting apoptosis, the cell cycle and the cytoskeleton, and to inhibitors of PARP, topoisomerase I and the ERK signaling pathway. AKT-INHIBITOR-VIII and MK-2206, two AKT1/1 inhibitors, were found efficient on BRCA cells. Indeed, the PI3K/mTOR pathway is commonly deregulated in breast cancer[11]. The 15 drugs to which cell lines of the GLSR were sensitive included seven PIK3/mTor inhibitors. Kinase inhibitors and chemotherapy agents targeting the mitotic spindle were also identified as potentially effective drugs against GLSR cells. Chemotherapy agents (gemcitabine, bleomycin, vinblastine) were the most active drugs for killing cells from the Mixed 1 cell line cluster. The other two heterogeneous clusters were each sensitive to only one drug: midostaurin-a PI3K/mTOR inhibitor for Mixed 2, and vorinostat, a HDAC inhibitor, for Mixed 3. Finally, the cell lines of the EDOT cluster were sensitive only to the chemotherapy agent doxorubicin.

Resistance profiles were identified for 163 associations between cell line clusters and drugs. More than 30% of these associations involved inhibitors of the ERK and PI3K/mTOR signaling pathways. All but one of the clusters appeared to be resistant to at least three drugs targeting this pathway. The exception was the Mixed 2 cluster, which was resistant only to the MEK1/2 inhibitor AZD6244). The cell lines of the various clusters were otherwise resistant to

a broad range of diverse drugs. The cell lines of the SCLC cluster were resistant to the largest number of molecules (22% of all the drugs tested), including, drugs targeting the apoptosis, NOTCH or Wnt signaling pathways. Drugs targeting the cytoskeleton, mitosis and replication appeared to be the least effective for killing the cell lines of the GI cluster. By contrast, the cell lines of the ADG cluster were found to be resistant to a drug targeting the cytoskeleton, a drug targeting the Wnt pathway and a drug targeting G-protein–coupled receptors. Topoisomerase I inhibitors, such as topotecan and camptothecin, were found ineffective to kill BRCA cells. Surprisingly, gemcitabine and methotrexate were also found to be inefficient against these cells, despite their widespread use in clinical practice. Mixed clusters presented only a small number of resistant associations.

References

1. Haibe-Kains, B. *et al.* Inconsistency in large pharmacogenomic studies. *Nature* **504,** 389–93 (2013).
2. Barretina, J., Caponigro, G. & Stransky, N. The Cancer Cell Line Encyclopedia enables predictive modelling of anticancer drug sensitivity. *Nature* **483,** 603–607 (2012).
3. Greshock, J. *et al.* Molecular target class is predictive of in vitro response profile. *Cancer Res.* **70,** 3677–3686 (2010).
4. Prickett, T. D. *et al.* Analysis of the tyrosine kinome in melanoma reveals recurrent mutations in ERBB4. *Nat. Genet.* **41,** 1127–1132 (2009).
5. Haverty, P. M. *et al.* Reproducible pharmacogenomic profiling of cancer cell line panels. *Nature* **533,** 333–337 (2016).
6. Van Den Bent, M. J. *et al.* Randomized phase II trial of erlotinib versus temozolomide or carmustine in recurrent glioblastoma: EORTC brain tumor group study 26034. *J. Clin. Oncol.* **27,** 1268–1274 (2009).
7. Raizer, J. J. *et al.* A phase II trial of erlotinib in patients with recurrent malignant gliomas and nonprogressive glioblastoma multiforme postradiation therapy. *Neuro. Oncol.* **12,** 95–103 (2010).
8. Wyman, K., Kelley, M., Puzanov, I. & Sanders, K. Phase II study of erlotinib given daily for patients with metastatic melanoma (MM). *J. Clin.* (2006).
9. Shigematsu, H. *et al.* Clinical and Biological Features Associated With Epidermal Growth Factor Receptor Gene Mutations in Lung Cancers. *JNCI J. Natl. Cancer Inst.* **97,** 339–346 (2005).
10. Hoeflich, K. P. *et al.* In vivo antitumor activity of MEK and phosphatidylinositol 3-kinase inhibitors in basal-like breast cancer models. *Clin Cancer Res* **15,** 4649–4664

(2009).

11. Tsimberidou, A.-M. *et al.* Personalized Medicine in a Phase I Clinical Trials Program: The MD Anderson Cancer Center Initiative. *Clin. Cancer Res.* **18,** 6373–6383 (2012).

Supplementary Fig.1

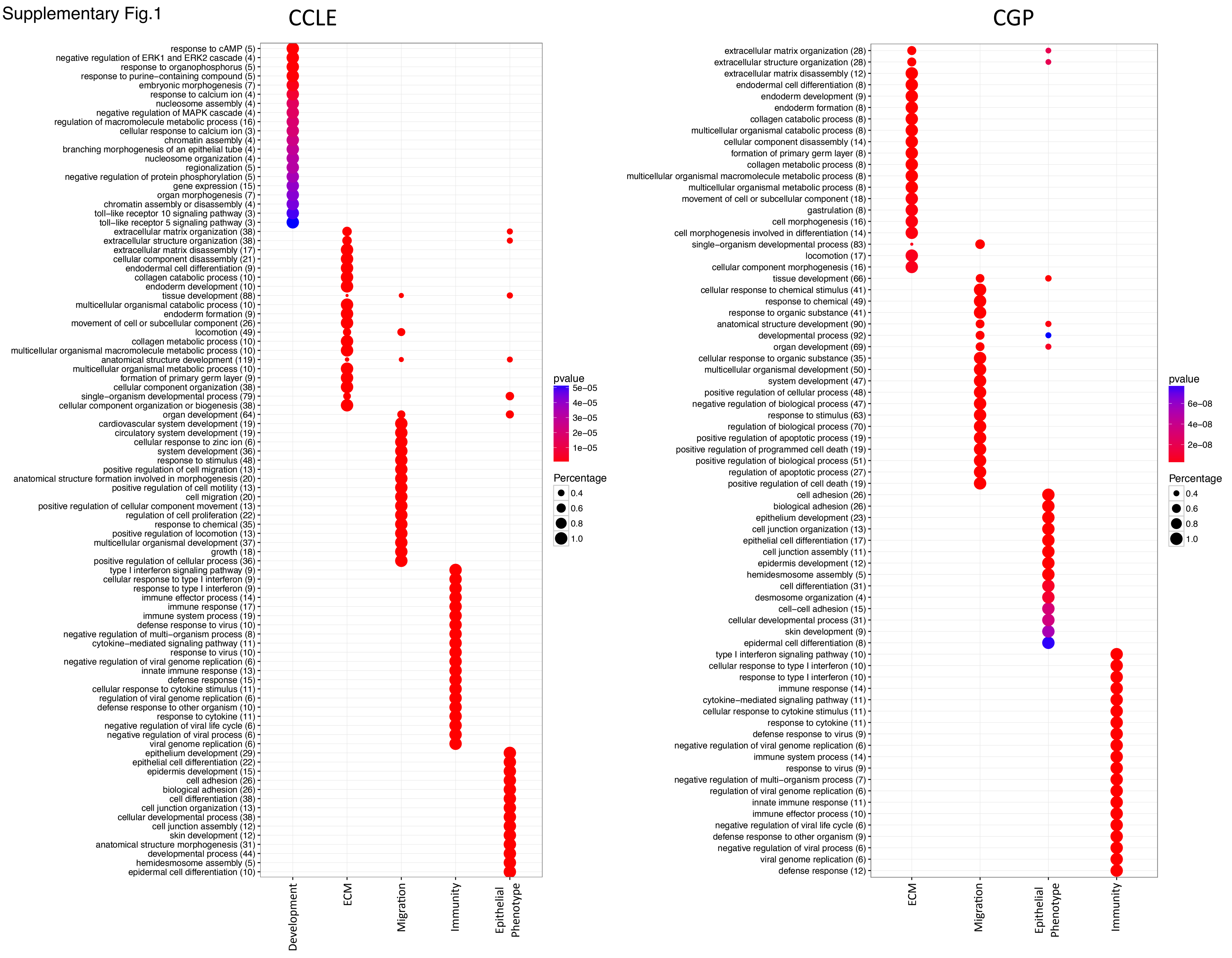

Supplementary Fig.2

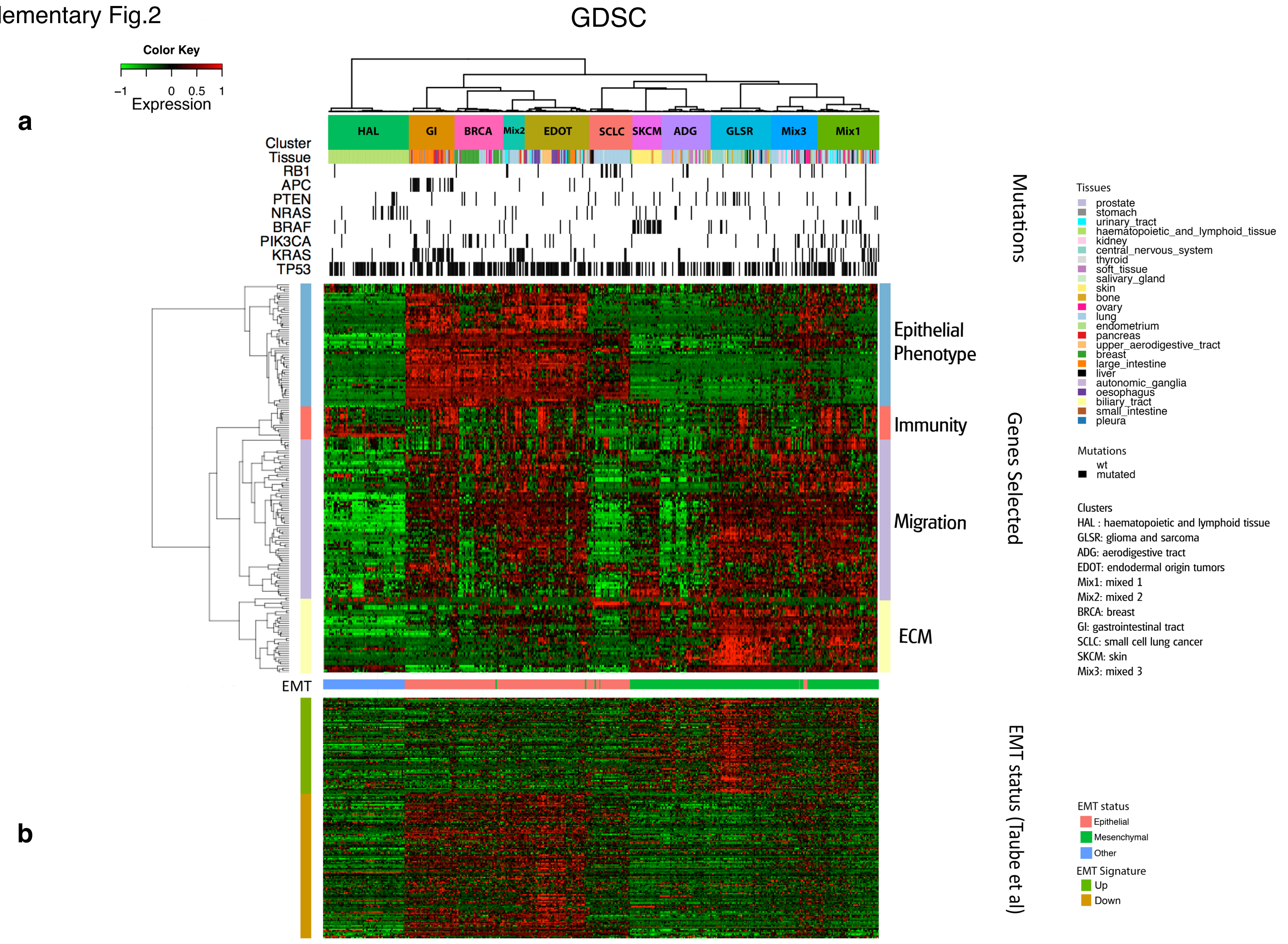

Supplementary Fig.3

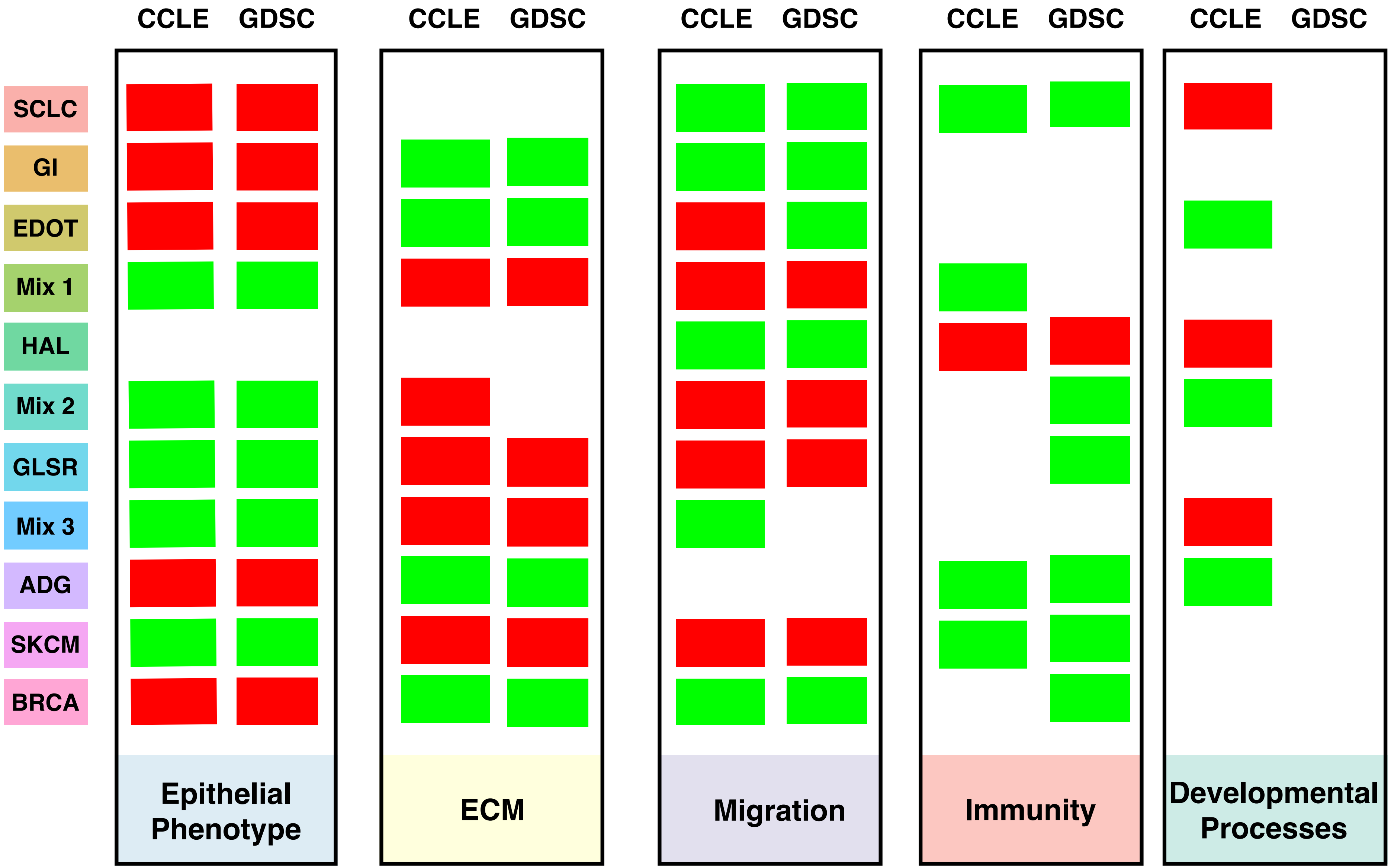

Supplementary Fig.4

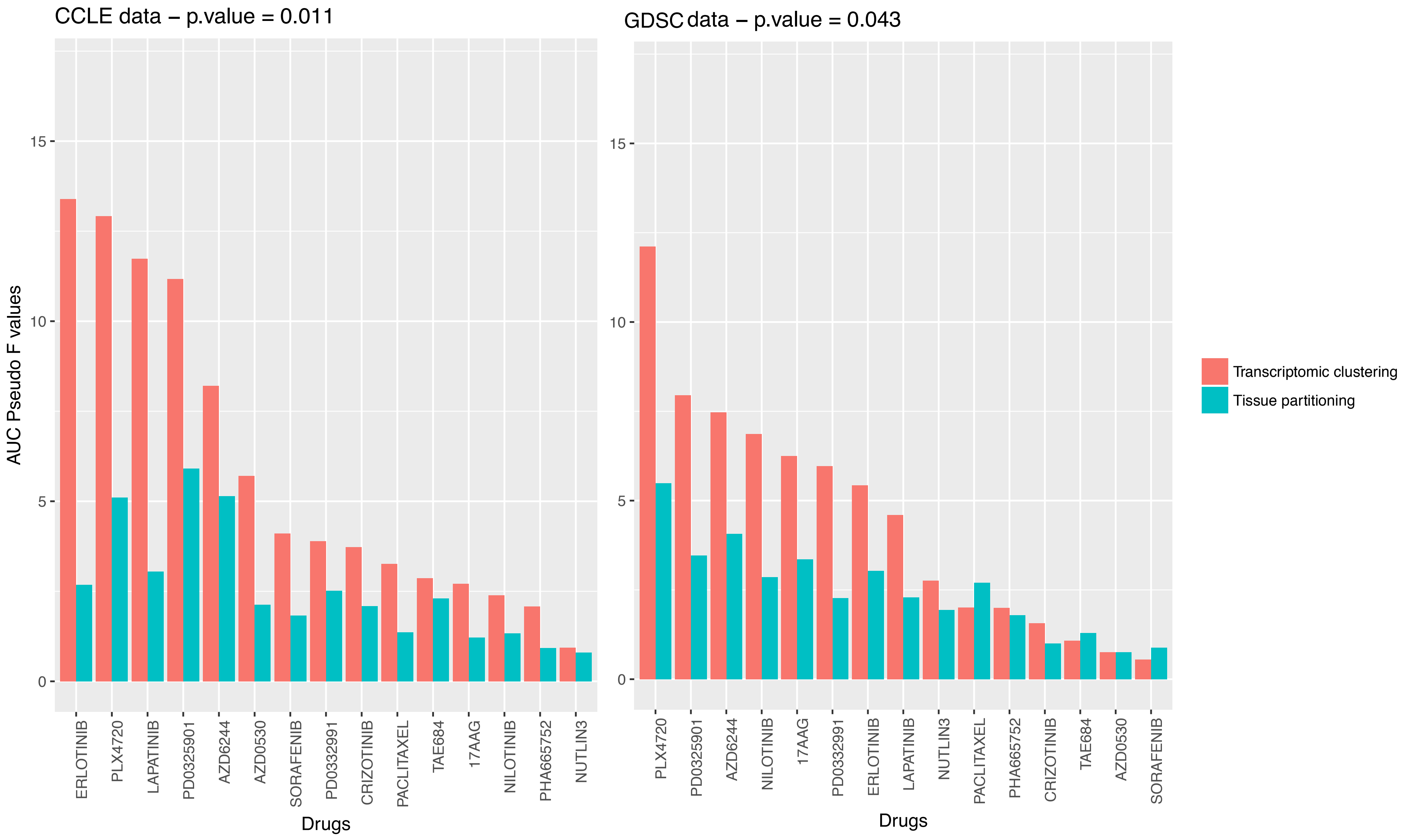

Supplementary Fig.5

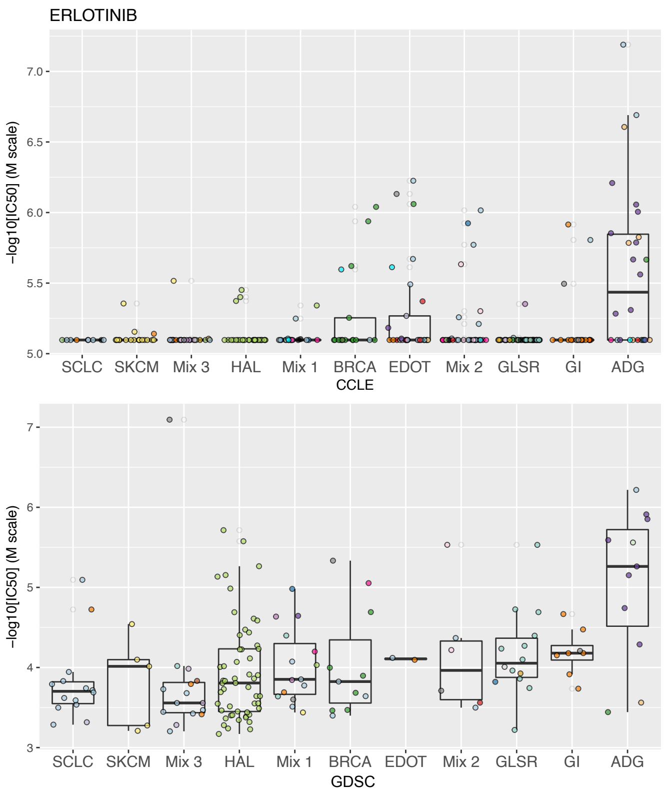

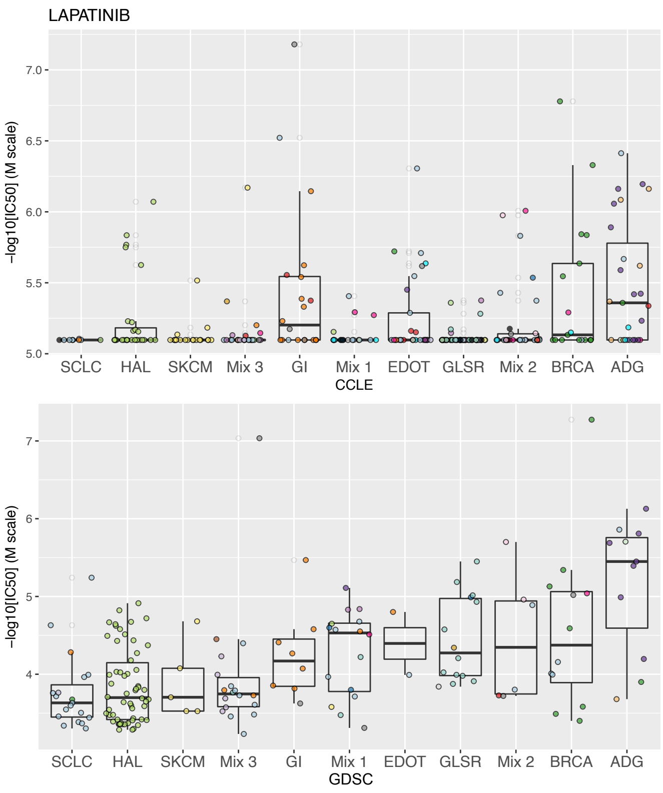

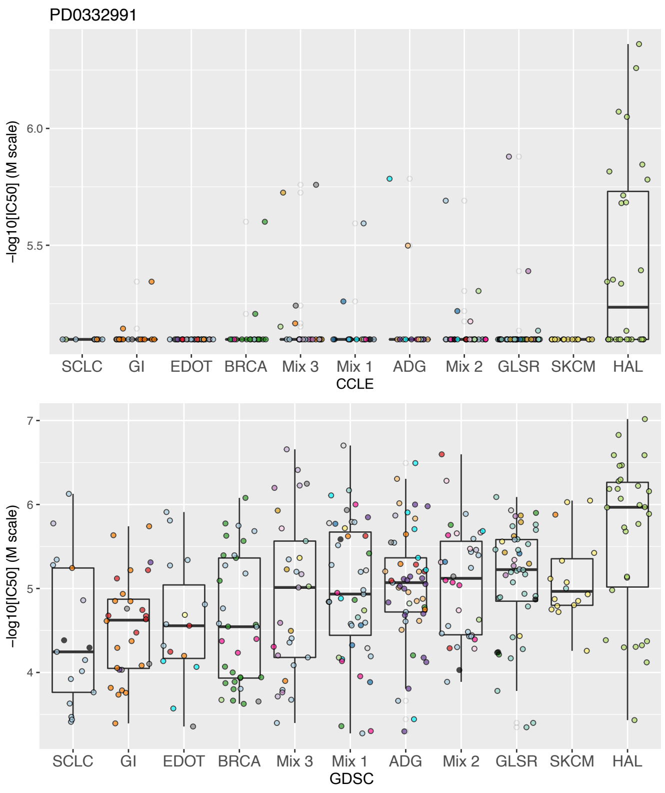

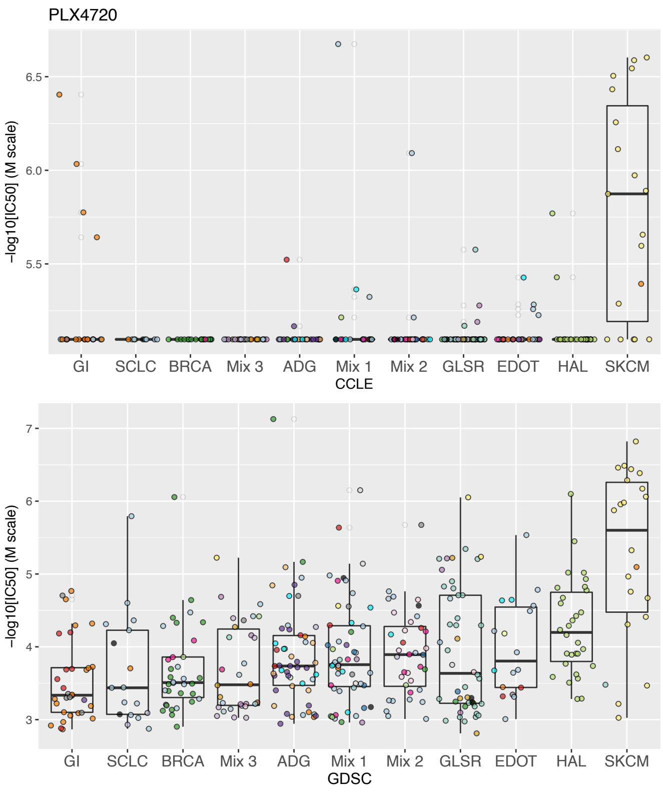

Supplementary Fig.5 (continue)

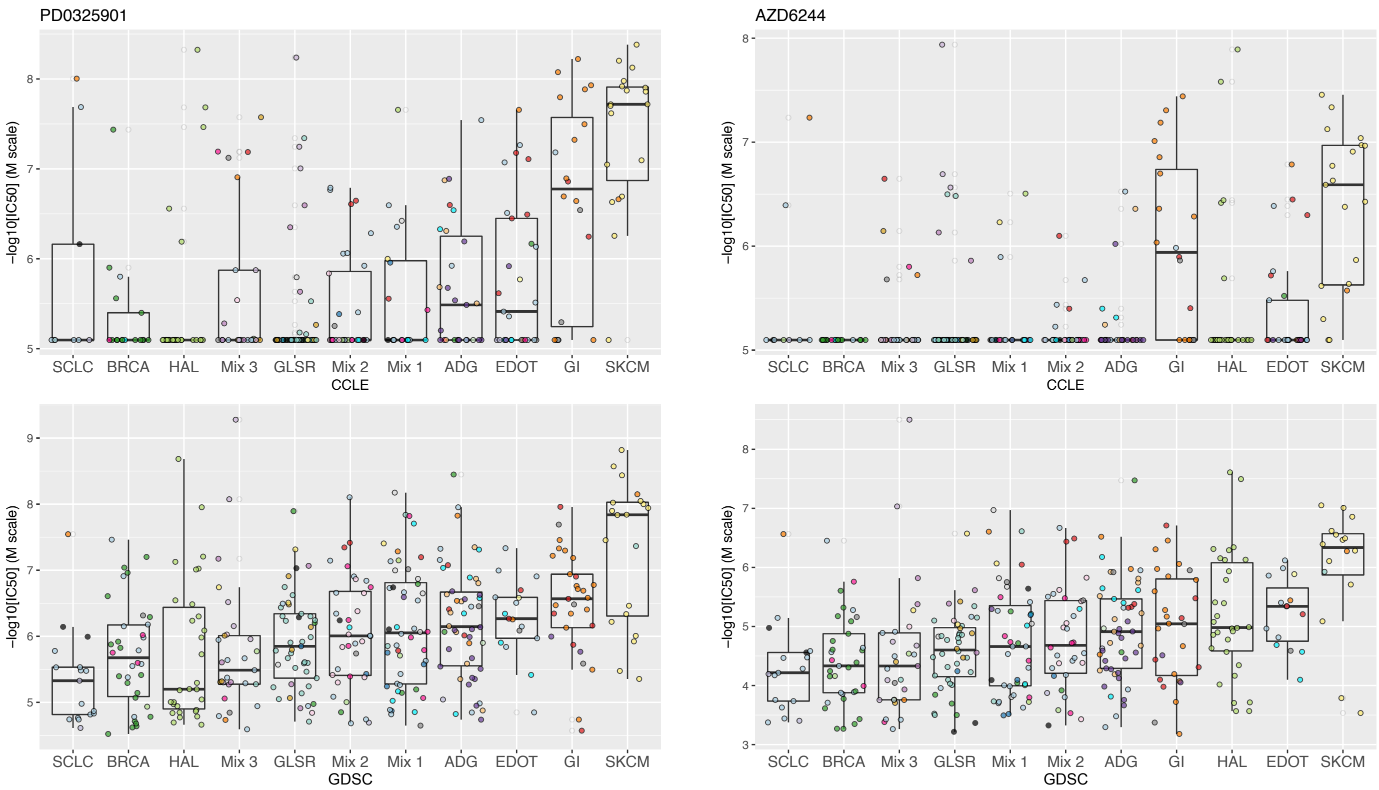

Supplementary Fig.6

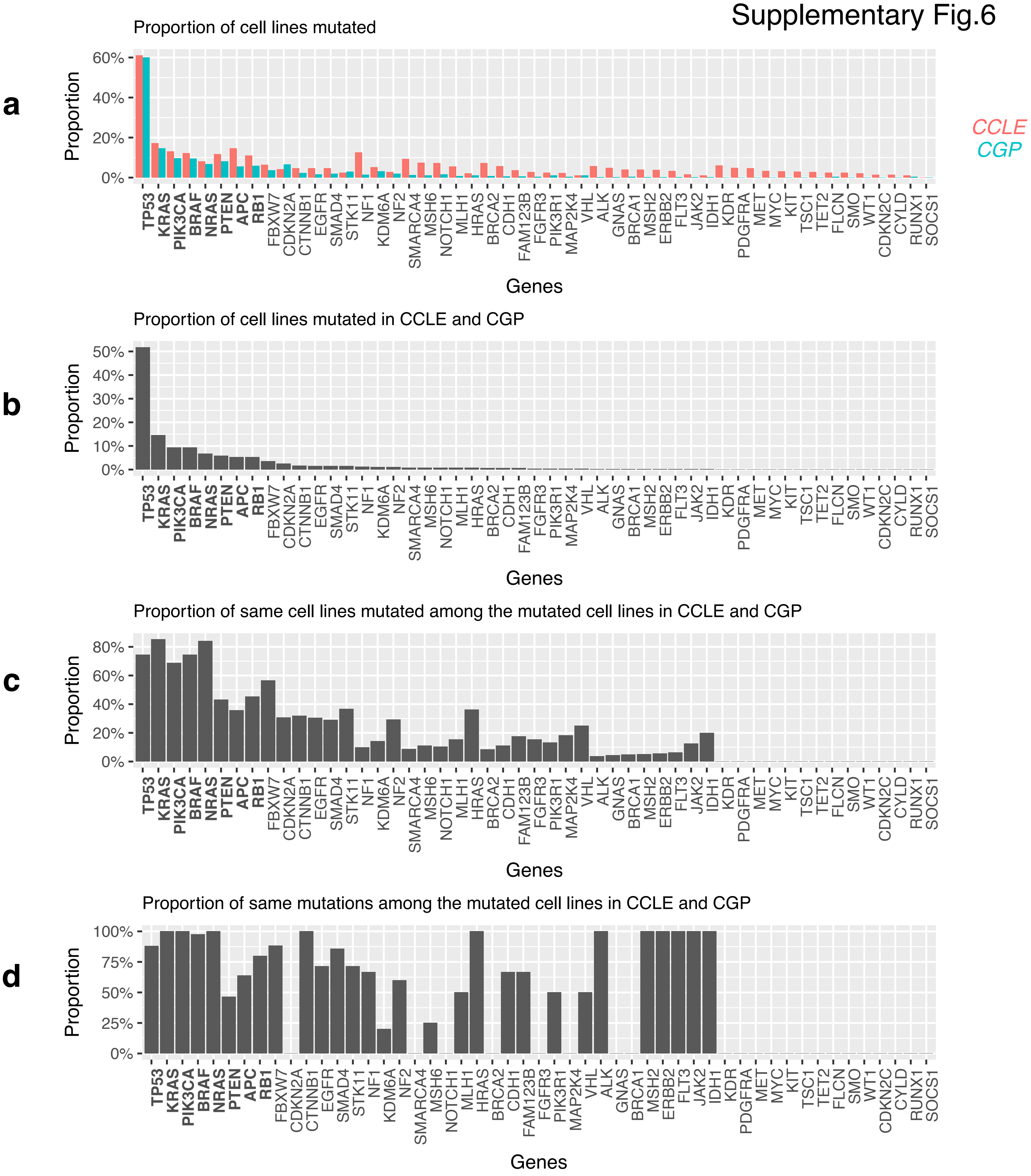

Supplementary Fig.7

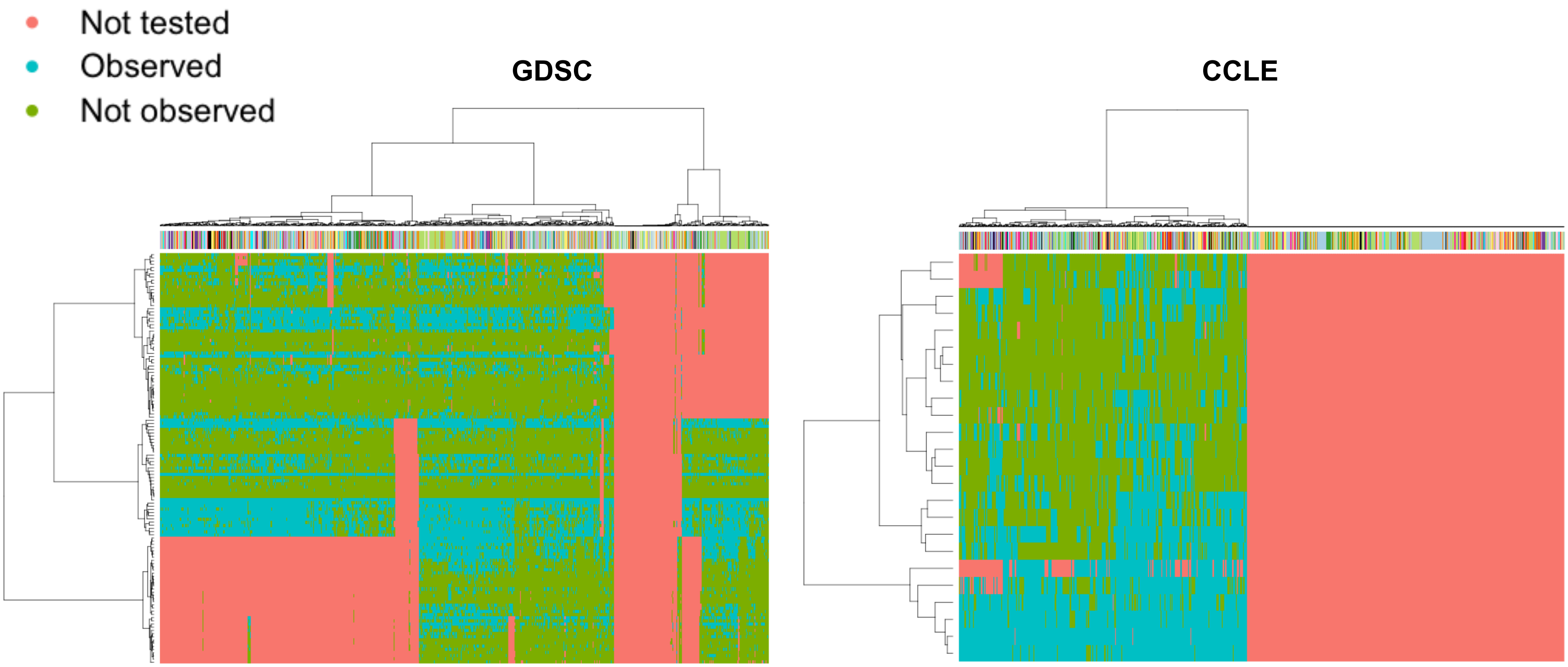

Supplementary Fig.8

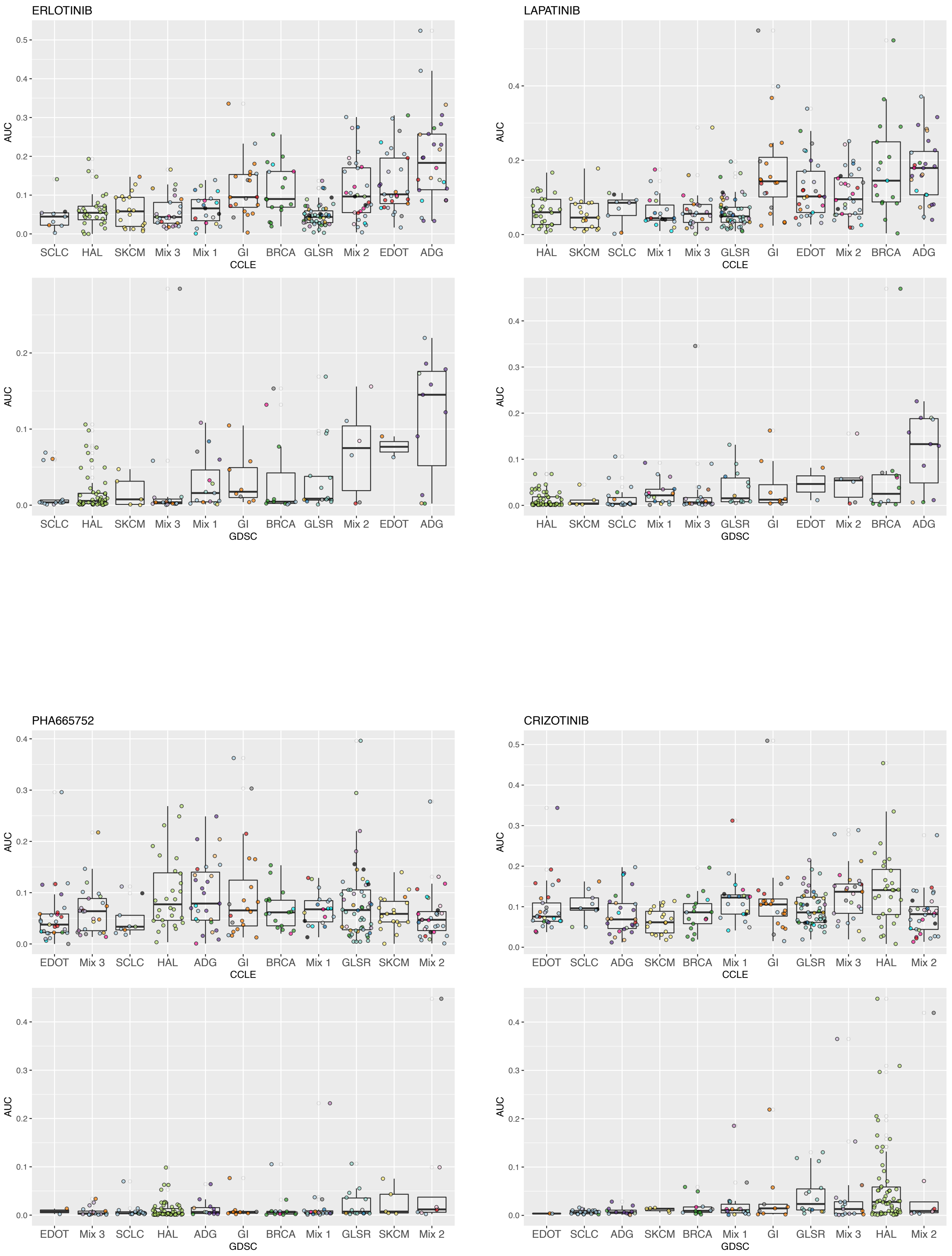

Supplementary Fig.8 (continue)

TAE684

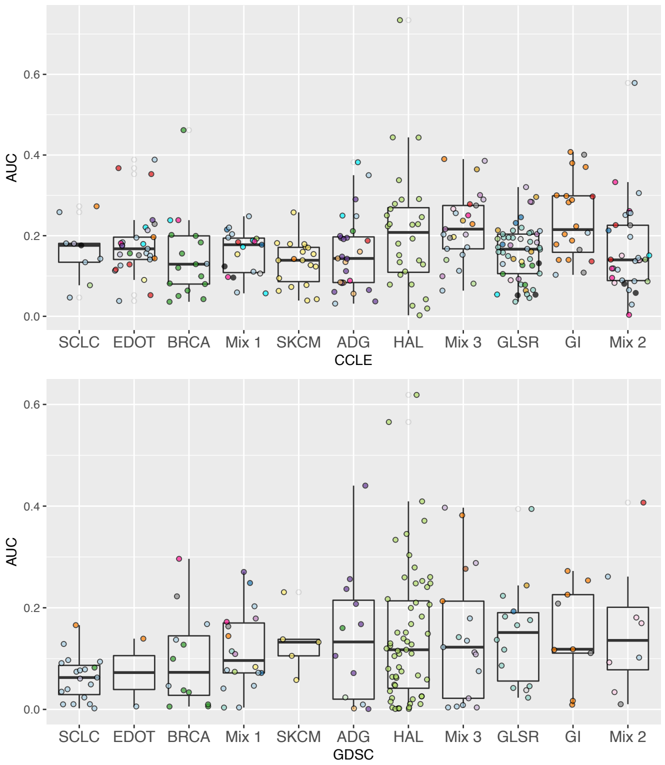

NILOTINIB

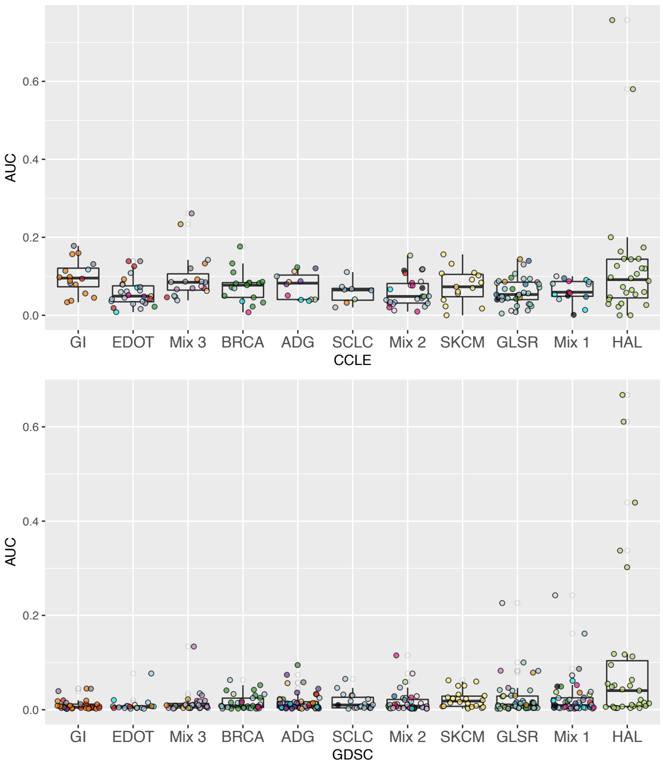

AZD0530

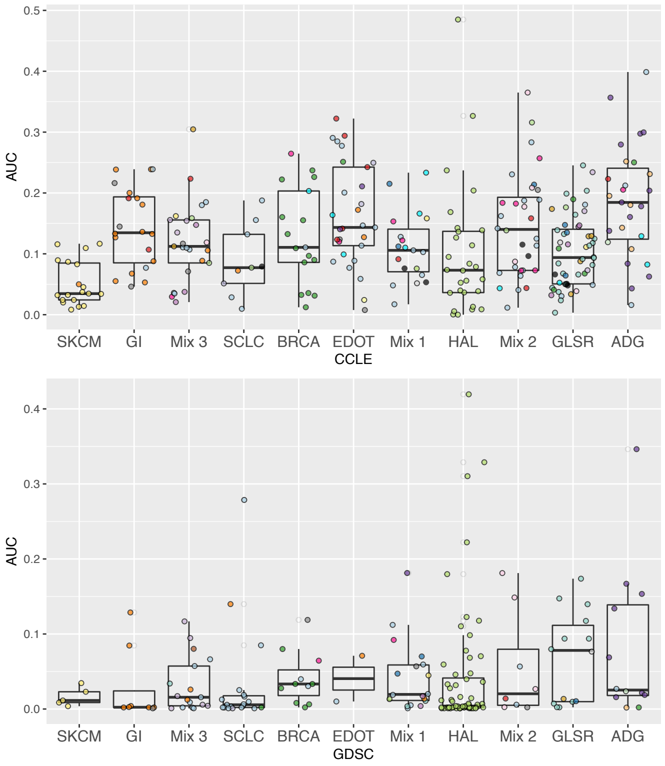

SORAFENIB

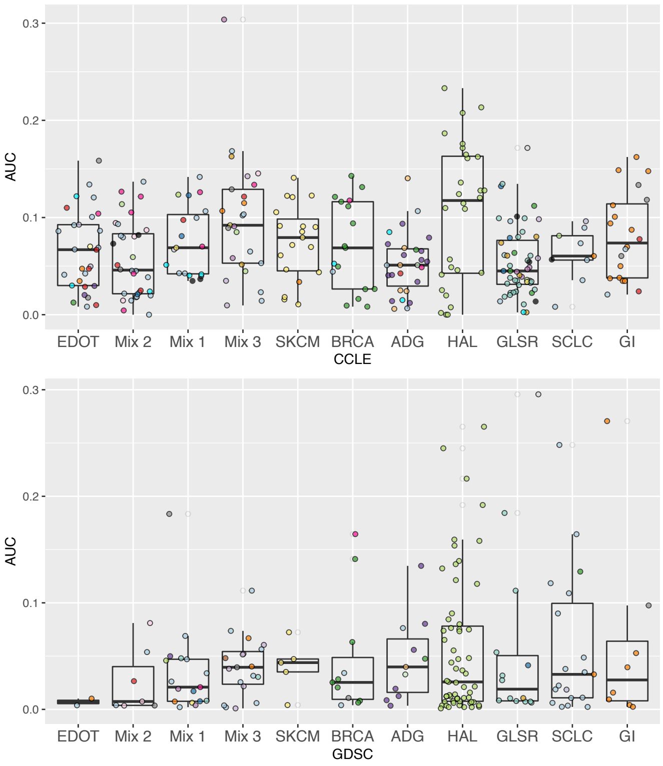

Supplementary Fig.8 (continue)

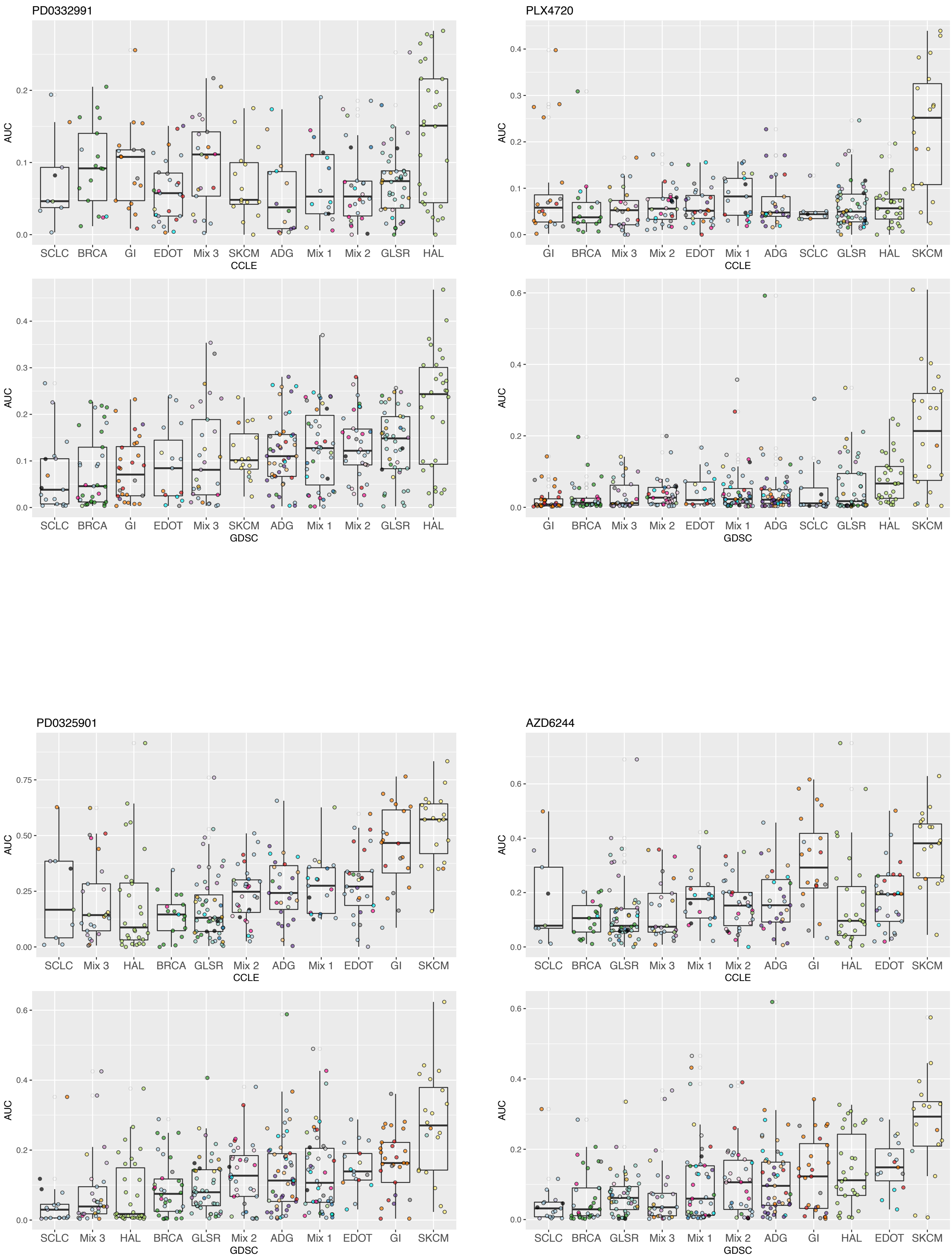

Supplementary Fig.8 (continue)

NUTLIN3

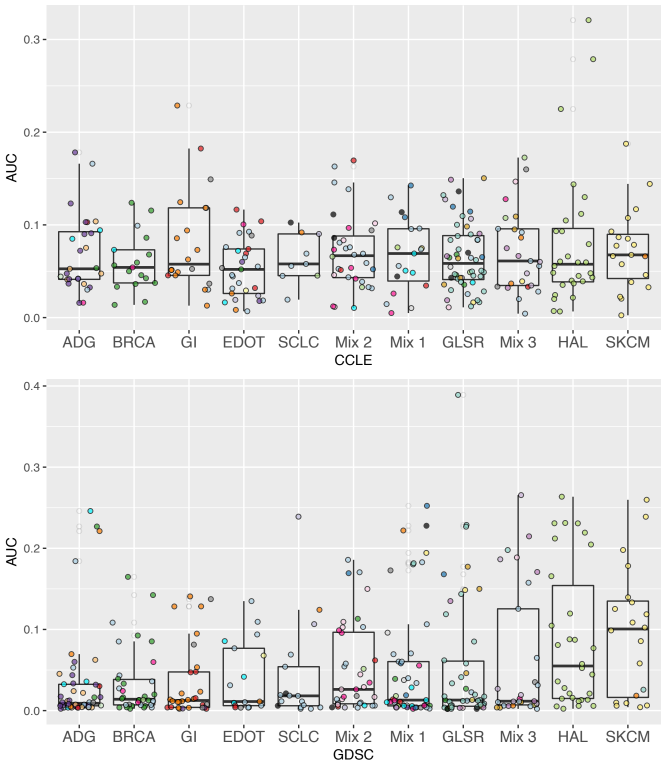

17AAG

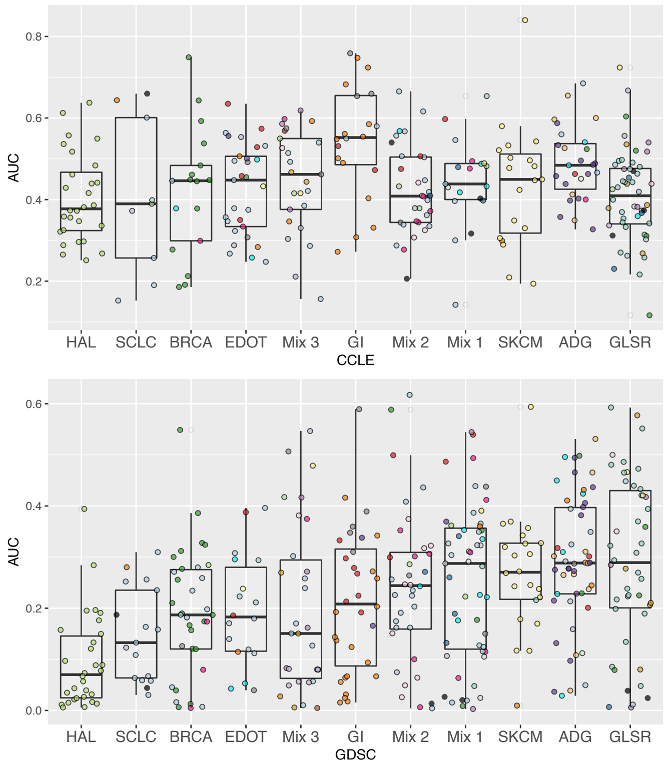

PACLITAXEL

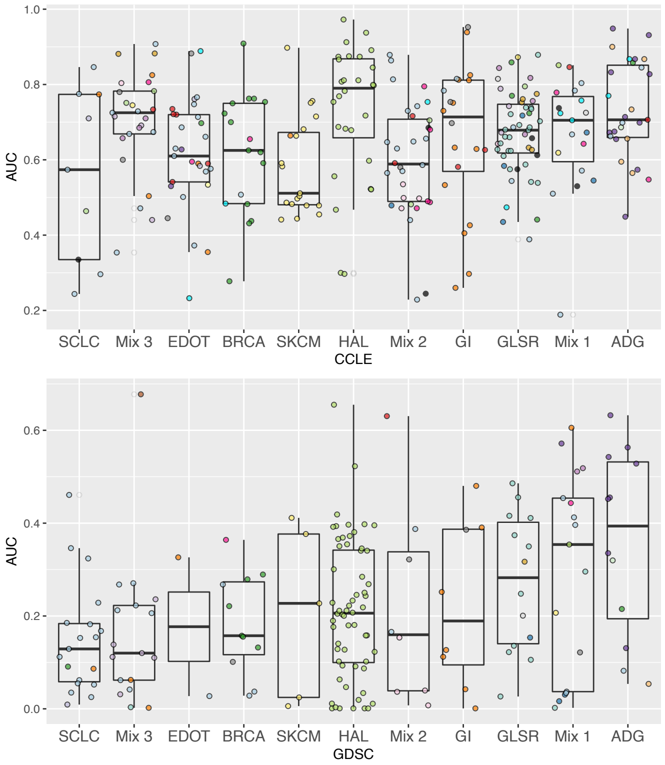

Supplementary Fig.9

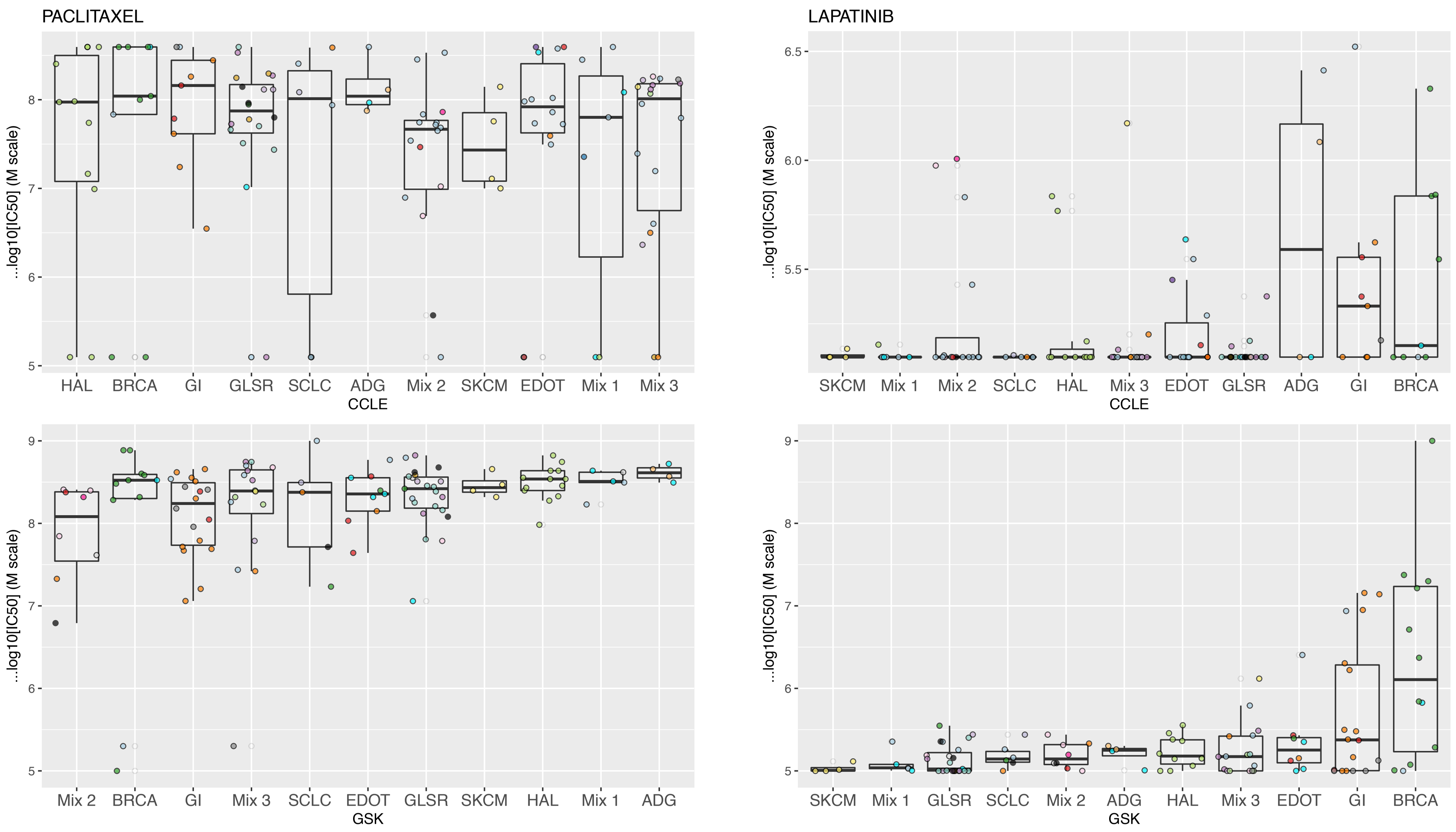

Supplementary Fig.10

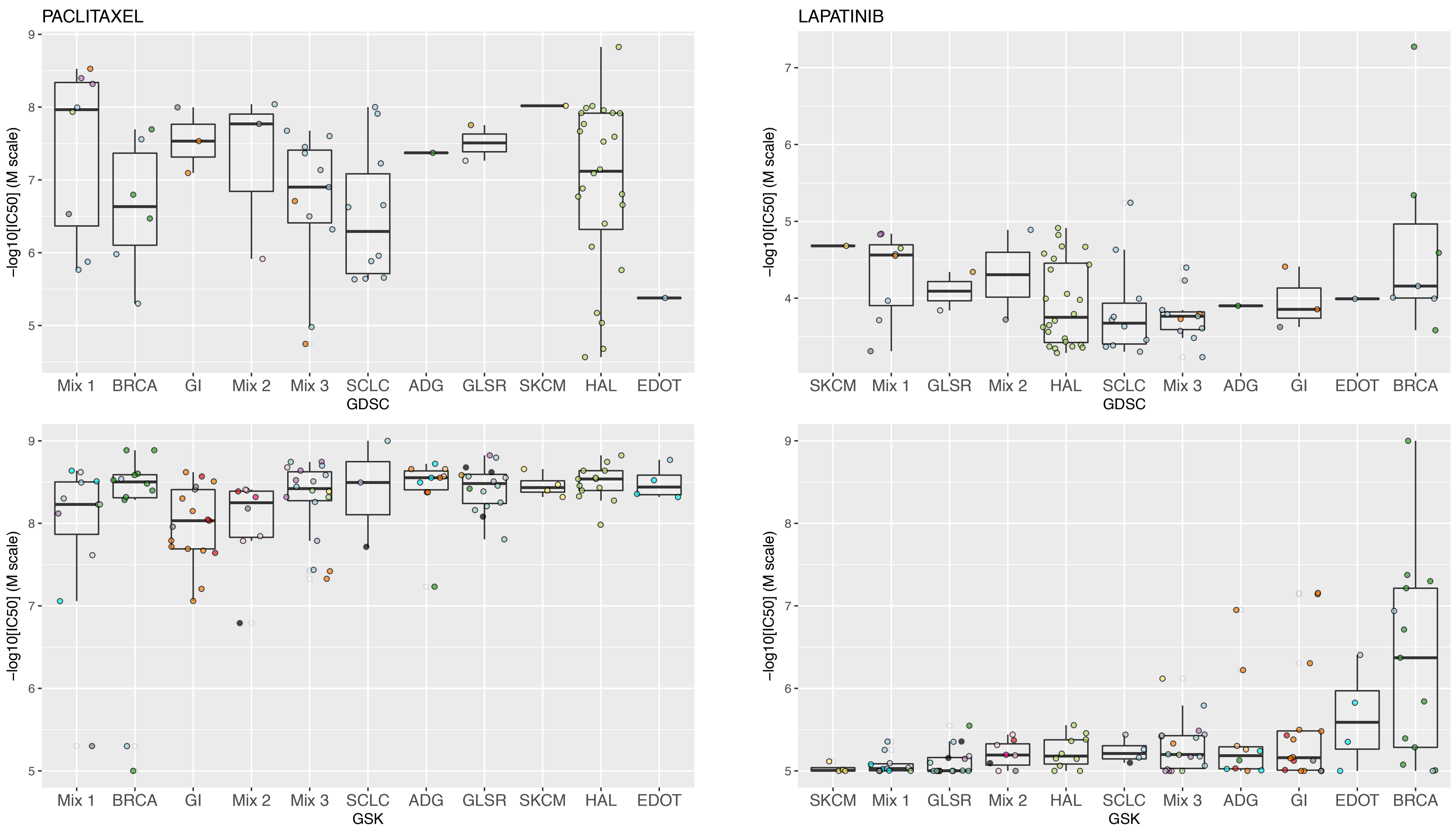

Supplementary Fig.11

ERLOTINIB

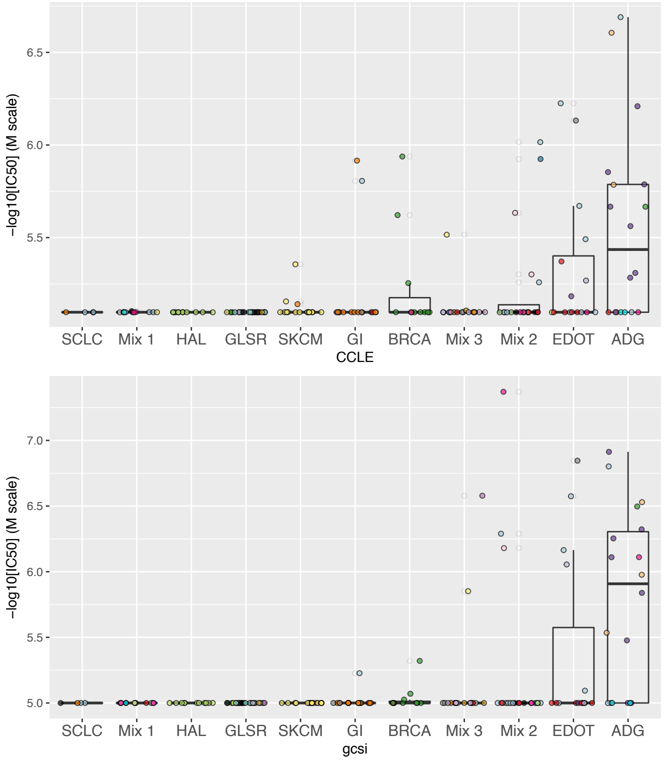

LAPATINIB

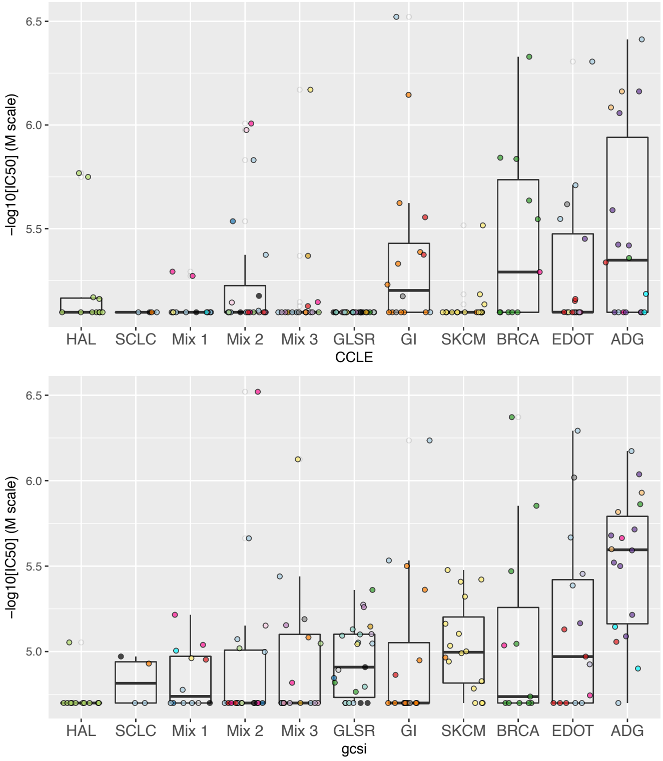

CRIZOTINIB

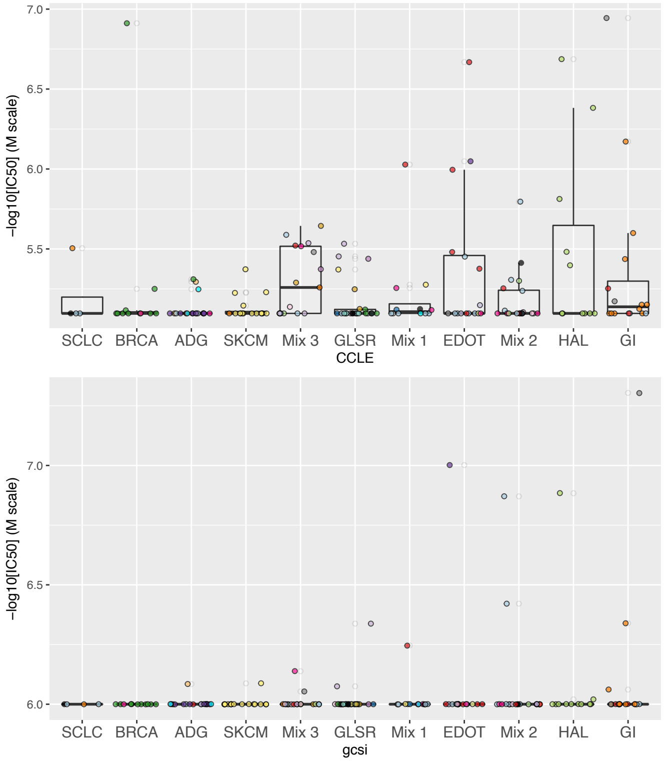

PD0325901

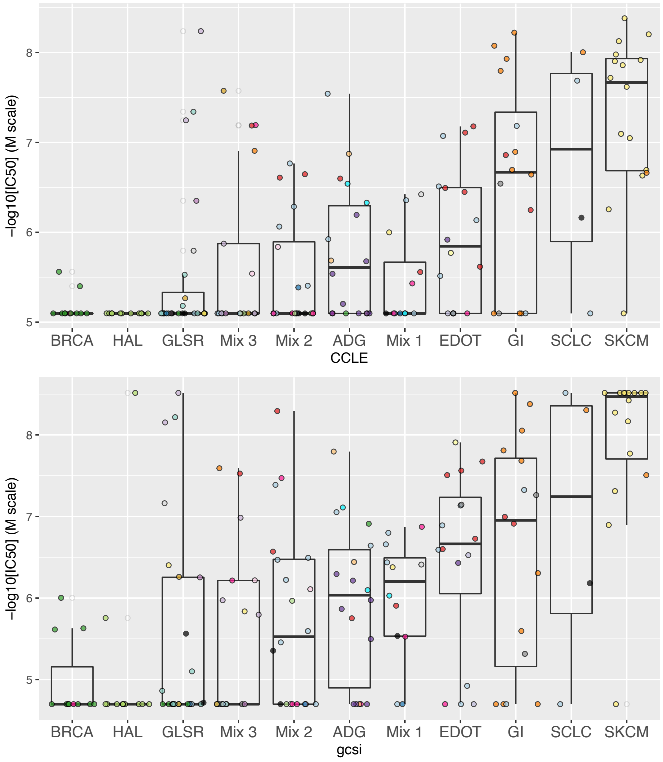

Supplementary Fig.11 (continue)

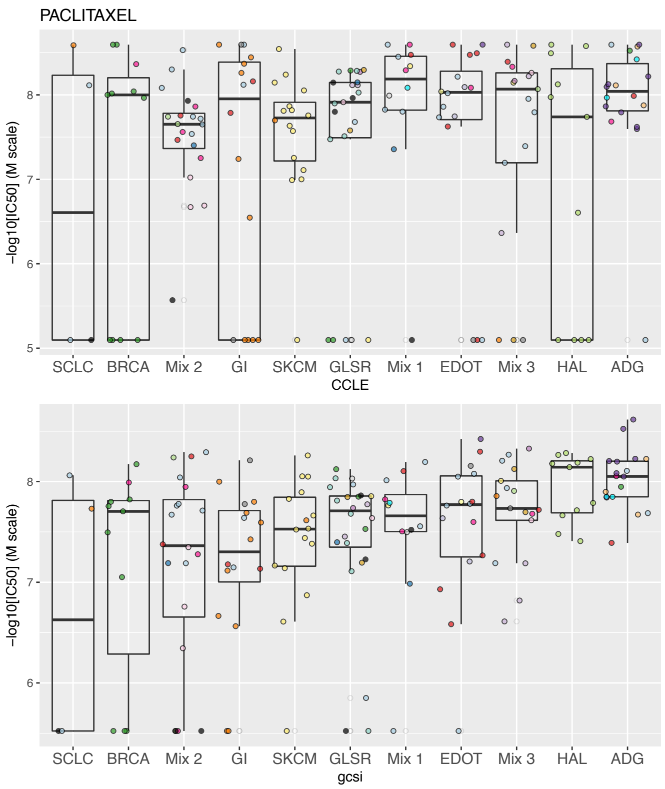

Supplementary Fig.12

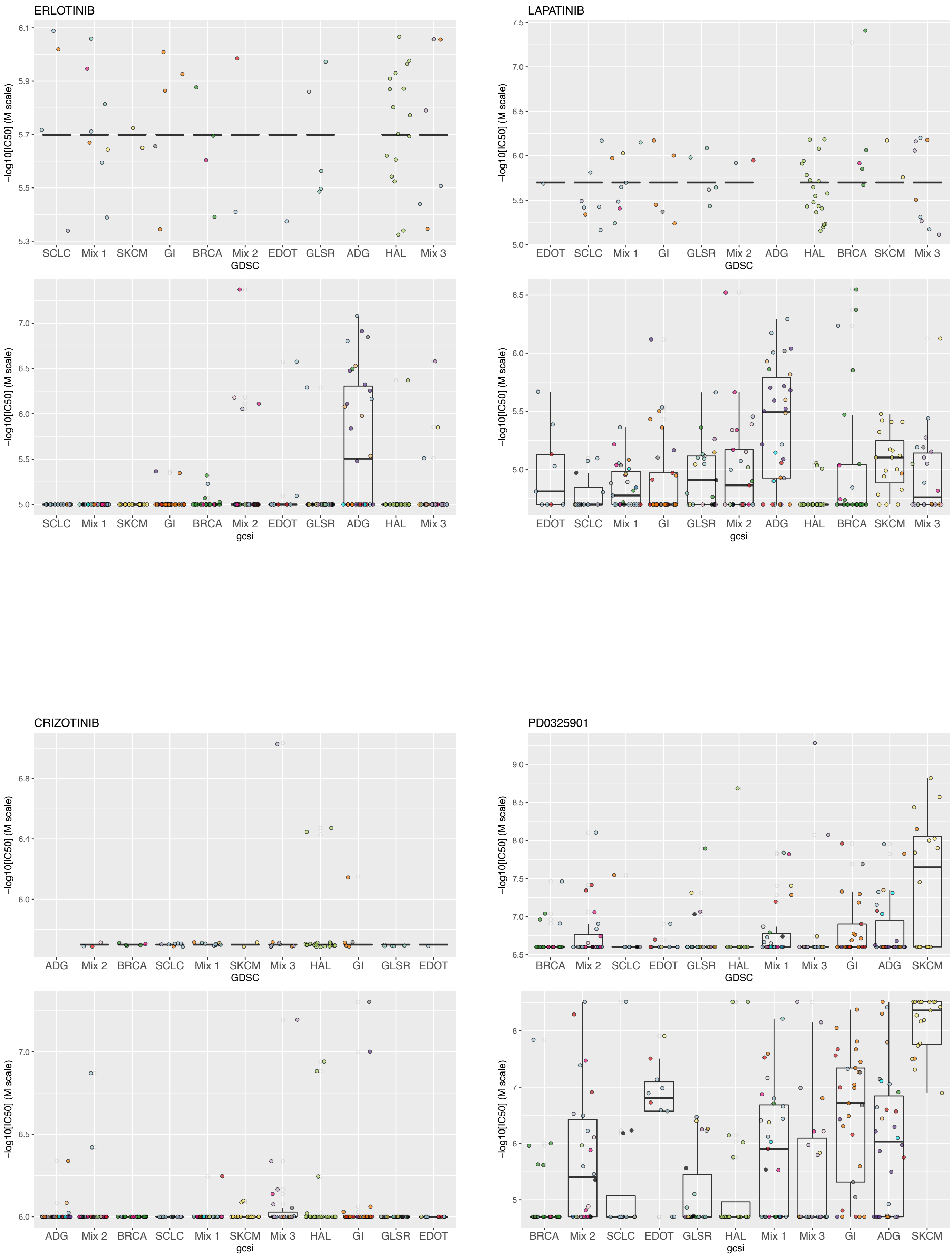

Supplementary Fig.12 (continue)

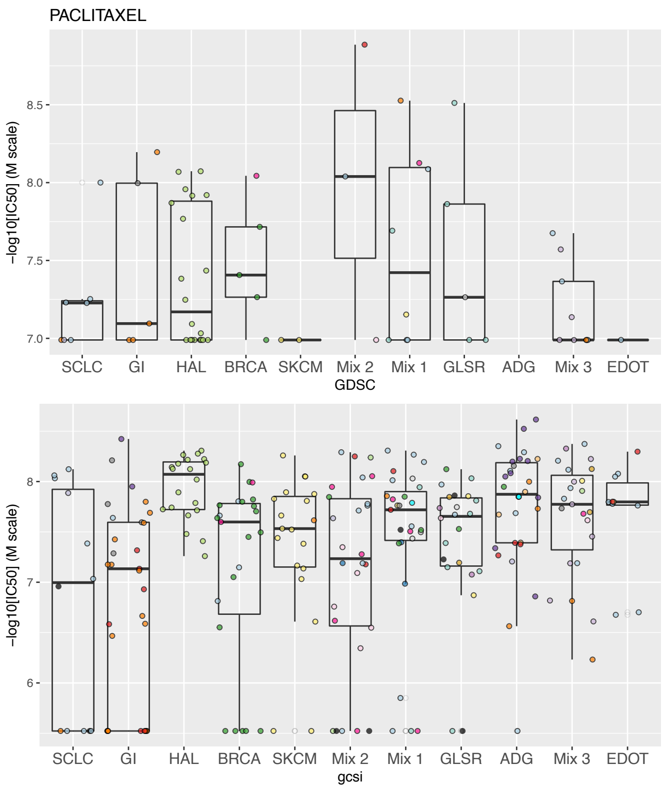

Supplementary Fig.13

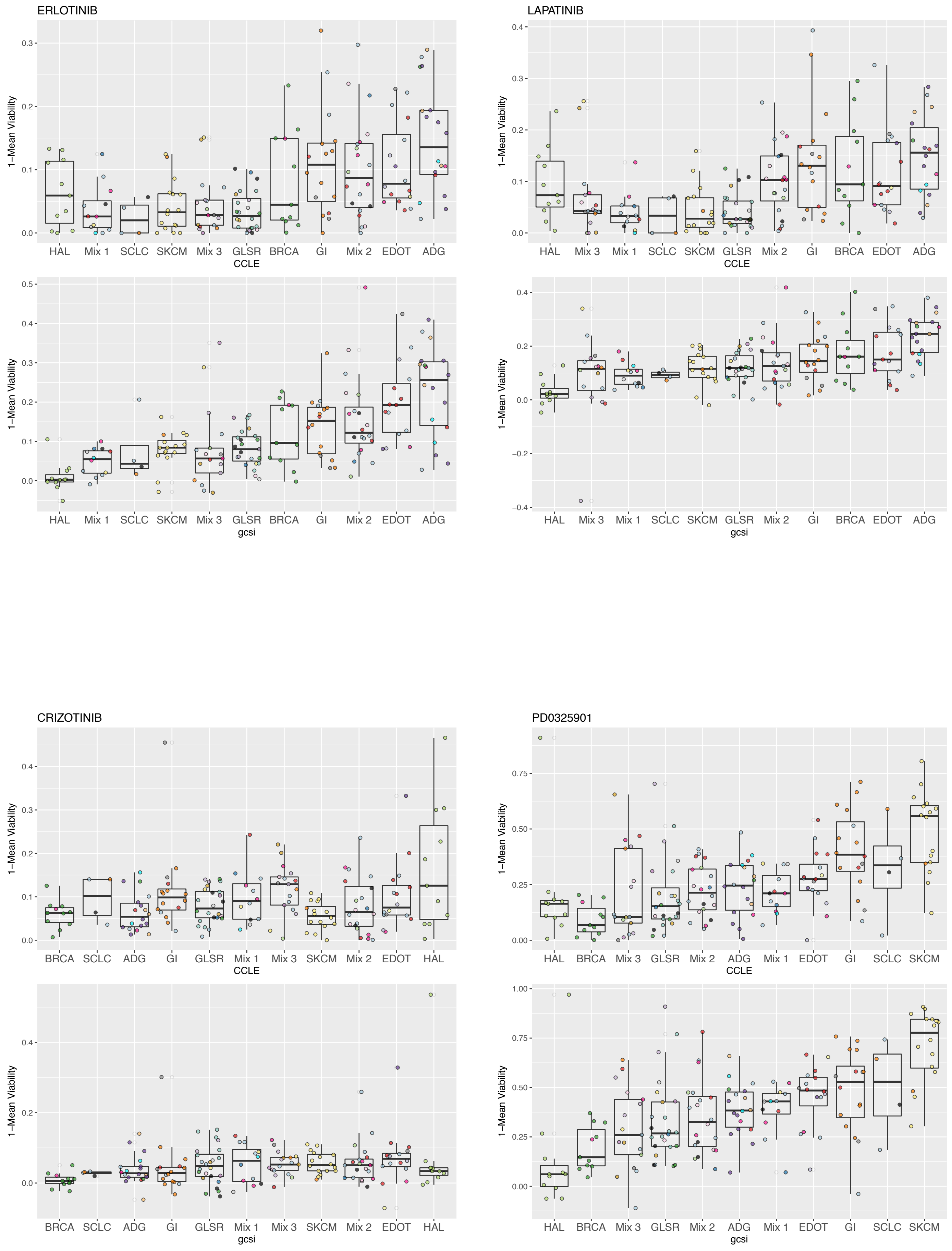

Supplementary Fig.13 (continue)

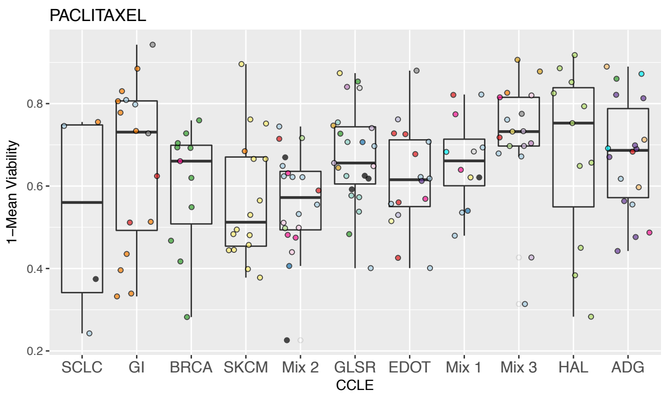

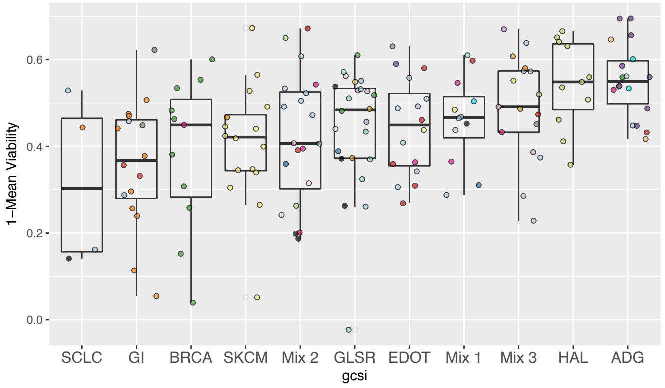

Supplementary Fig.14

ERLOTINIB

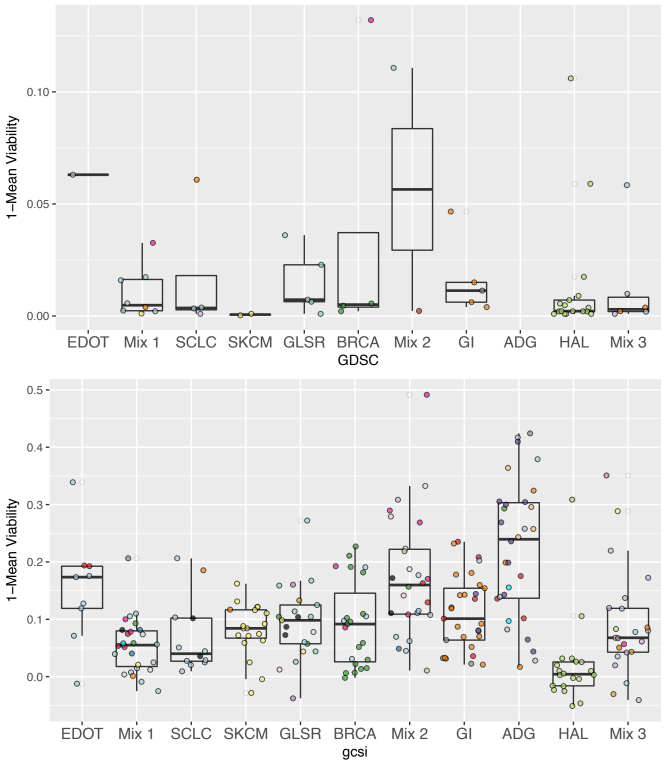

LAPATINIB

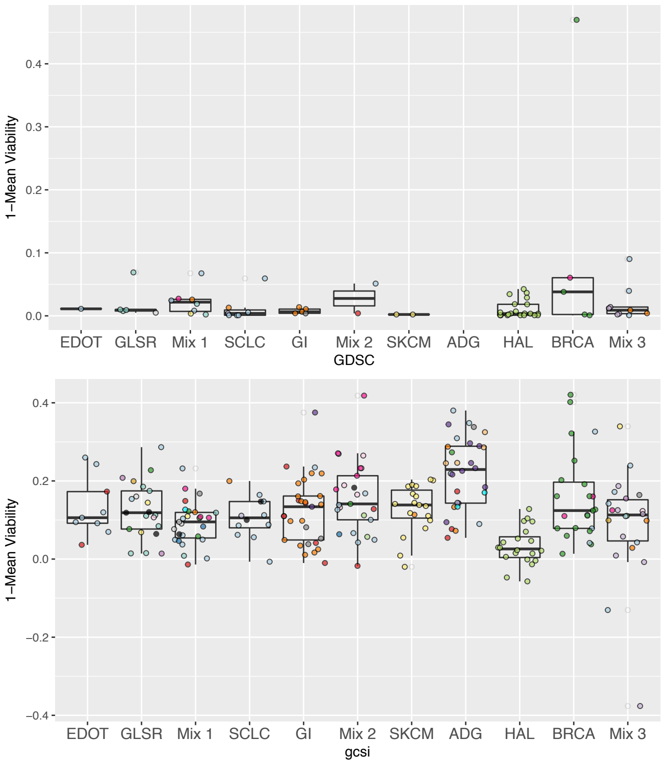

CRIZOTINIB

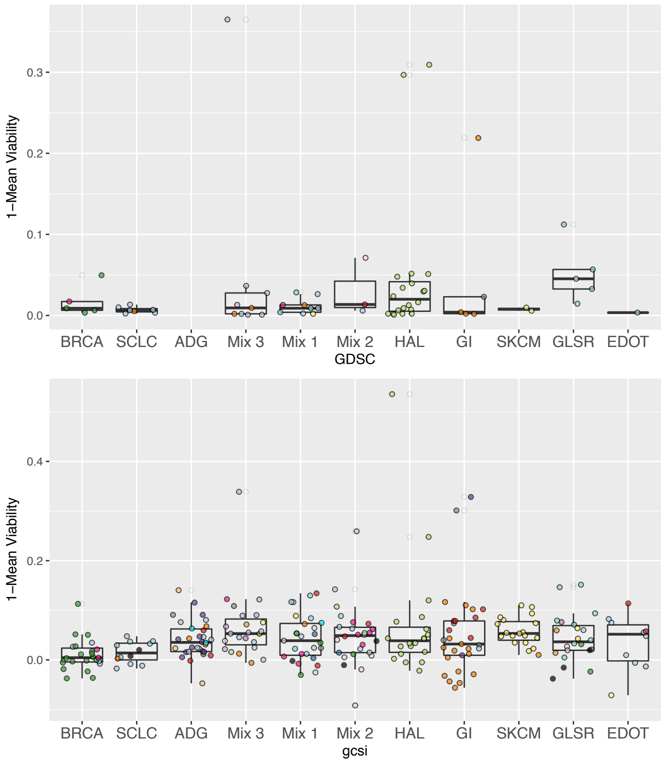

PD0325901

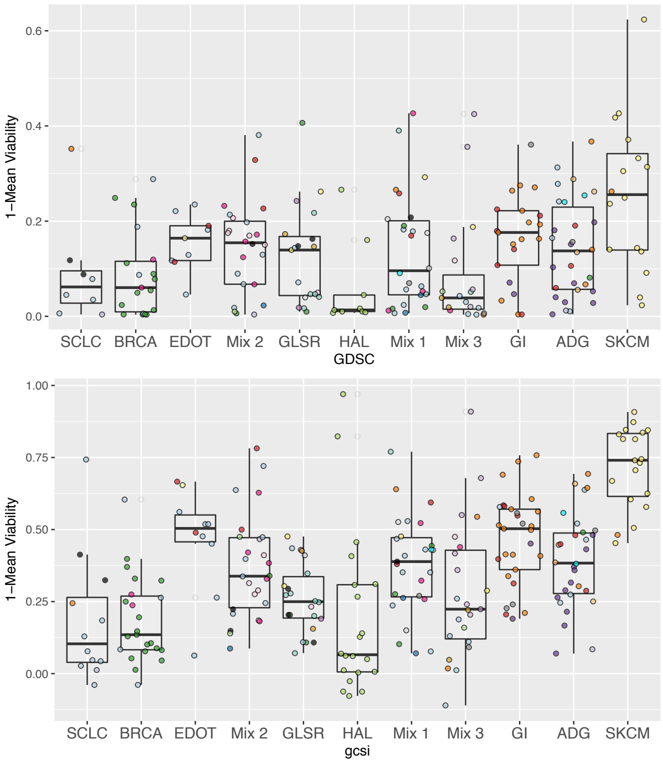

Supplementary Fig.14 (continue)

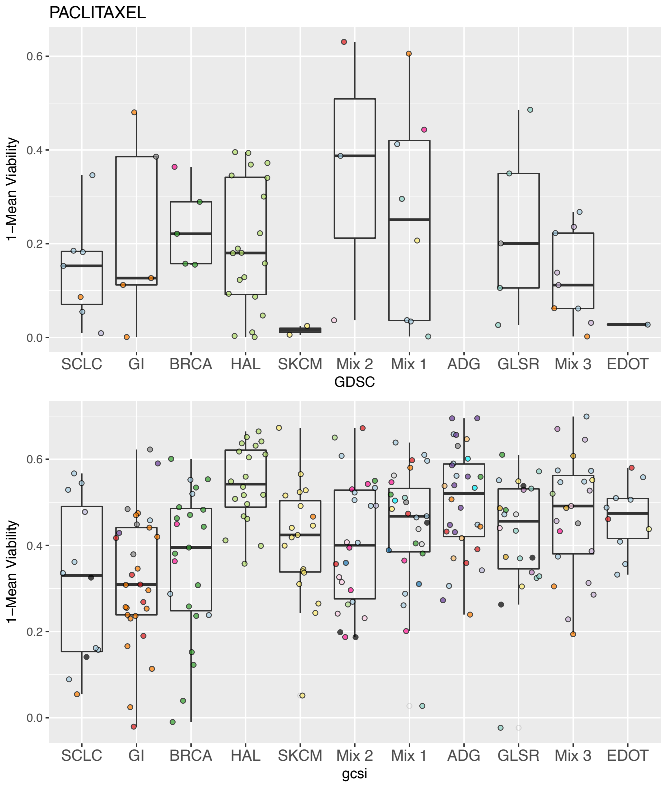

Supplementary Fig.15

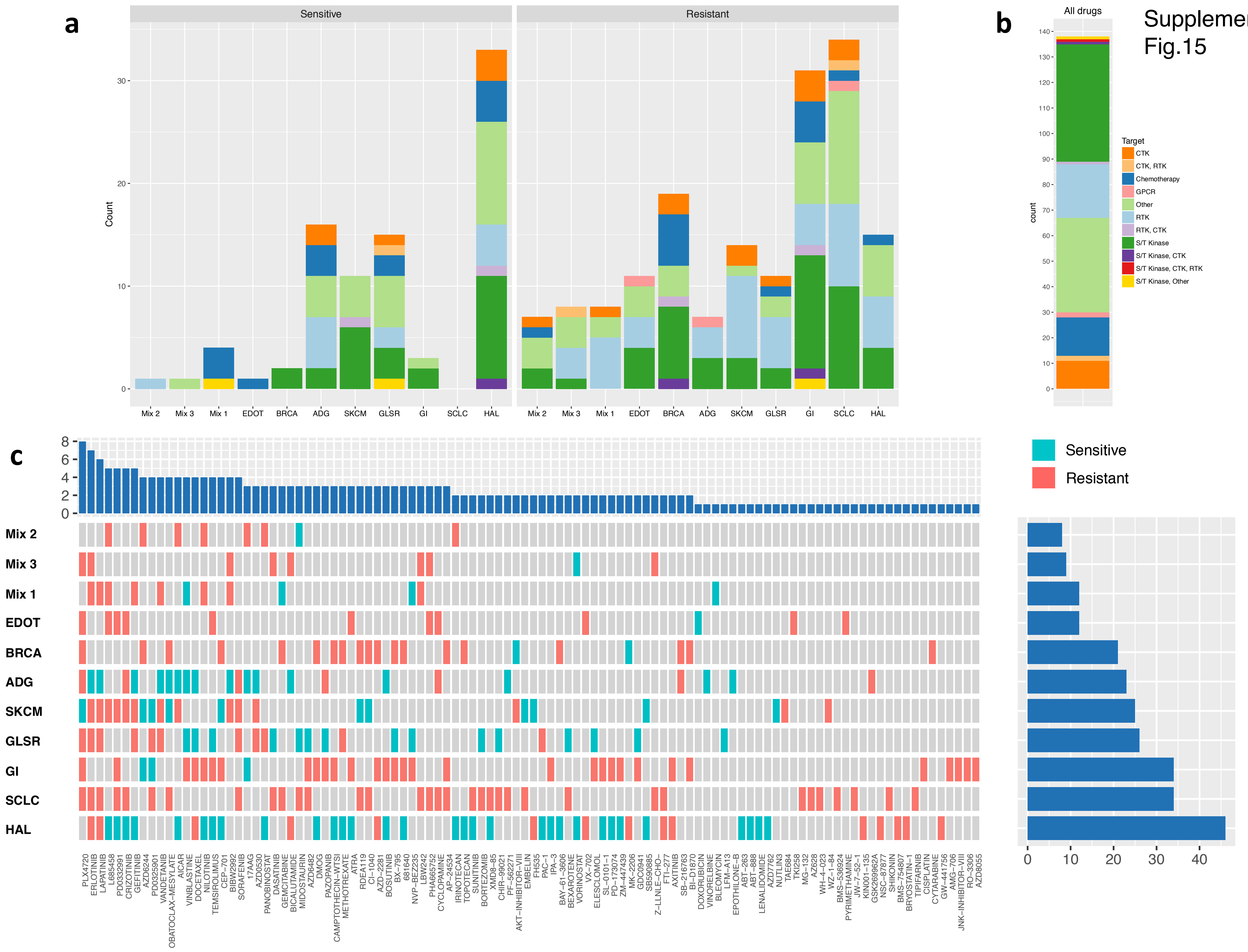

Supplementary Table 1

SCLC	TP53	KRAS	PIK3CA	BRAF	NRAS	PTEN	APC	RB1
Proportion in %	84	8	14	5	8	5	24	41
Fisher p-value	0,00443	0,957	0,662	0,978	0,492	0,864	0,174	0,000307
FDR-adjusted p-value	0,0297	1	1	1	0,983	1	0,959	0,00338

GI	TP53	KRAS	PIK3CA	BRAF	NRAS	PTEN	APC	RB1
Proportion in %	60	57	31	23	0	9	63	0
Fisher p-value	0,736	1,72E-07	0,00289	0,0521	1	0,684	5,62E-12	1
FDR-adjusted p-value	1	1,89E-06	0,0318	0,287	1	1	6,18E-11	1

EDOT	TP53	KRAS	PIK3CA	BRAF	NRAS	PTEN	APC	RB1
Proportion in %	82	44	9	4	7	0	7	16
Fisher p-value	0,0054	0,0000125	0,811	1	0,861	1	0,956	0,538
FDR-adjusted p-value	0,0297	0,0000688	1	1	1	1	0,997	1

Mixed 1	TP53	KRAS	PIK3CA	BRAF	NRAS	PTEN	APC	RB1
Proportion in %	48	48	10	19	19	5	14	5
Fisher p-value	0,96	0,00164	0,786	0,593	0,0762	1	0,609	1
FDR-adjusted p-value	1	0,00601	1	1	0,419	1	0,997	1

HAL	TP53	KRAS	PIK3CA	BRAF	NRAS	PTEN	APC	RB1
Proportion in %	64	8	3	3	23	11	8	5
Fisher p-value	0,584	0,983	1	0,999	0,0000602	0,738	0,997	0,941
FDR-adjusted p-value	1	1	1	1	0,000662	1	0,997	1

Mixed 2	TP53	KRAS	PIK3CA	BRAF	NRAS	PTEN	APC	RB1
Proportion in %	51	29	18	10	0	12	10	4
Fisher p-value	0,951	0,00862	0,157	0,744	1	0,703	0,862	0,949
FDR-adjusted p-value	1	0,0237	0,432	1	1	1	0,997	1

GLSR	TP53	KRAS	PIK3CA	BRAF	NRAS	PTEN	APC	RB1
Proportion in %	58	5	11	9	9	24	12	18
Fisher p-value	0,847	0,998	0,902	0,935	0,29	0,0236	0,865	0,208
FDR-adjusted p-value	1	1	1	1	0,797	0,259	0,997	0,763

Mixed 3	TP53	KRAS	PIK3CA	BRAF	NRAS	PTEN	APC	RB1
Proportion in %	59	11	14	14	0	16	11	19
Fisher p-value	0,801	0,818	0,563	0,766	1	0,505	0,829	0,0432
FDR-adjusted p-value	1	1	1	1	1	1	0,997	0,238

ADG	TP53	KRAS	PIK3CA	BRAF	NRAS	PTEN	APC	RB1
Proportion in %	77	3	23	3	8	8	13	0
Fisher p-value	0,0911	0,994	0,147	0,978	0,536	0,864	0,77	1
FDR-adjusted p-value	0,334	1	0,432	1	0,983	1	0,997	1

SKCM	TP53	KRAS	PIK3CA	BRAF	NRAS	PTEN	APC	RB1
Proportion in %	31	0	0	81	15	15	15	4
Fisher p-value	1	1	1	1,42E-10	0,163	0,375	0,578	1
FDR-adjusted p-value	1	1	1	1,56E-09	0,599	1	0,997	1

BRCA	TP53	KRAS	PIK3CA	BRAF	NRAS	PTEN	APC	RB1
Proportion in %	73	3	24	6	0	21	3	12
Fisher p-value	0,168	0,988	0,0713	0,838	1	0,152	0,992	0,534
FDR-adjusted p-value	0,462	1	0,392	1	1	0,836	0,997	1

Supplementary Table 2

SCLC	TP53	KRAS	PIK3CA	BRAF	NRAS	PTEN	APC	RB1
Proportion in %	72	6	6	0	3	9	6	31
Fisher p-value	0,0389	1	0,805	1	0,864	0,482	0,403	7,72E-06
FDR-adjusted p-value	0,139	1	1	1	1	1	1	0,000232

GI	TP53	KRAS	PIK3CA	BRAF	NRAS	PTEN	APC	RB1
Proportion in %	65	62	16	11	0	3	49	0
Fisher p-value	0,622	1,87E-08	0,124	0,783	1	0,916	5,68E-14	1
FDR-adjusted p-value	0,629	1,09E-06	0,319	1	1	1	6,63E-11	1

EDOT	TP53	KRAS	PIK3CA	BRAF	NRAS	PTEN	APC	RB1
Proportion in %	72	56	6	11	17	0	6	11
Fisher p-value	0,466	0,00104	0,85	0,769	0,0983	1	1	0,548
FDR-adjusted p-value	0,629	0,00014	1	1	1	1	1	1

Mixed 1	TP53	KRAS	PIK3CA	BRAF	NRAS	PTEN	APC	RB1
Proportion in %	58	28	21	15	8	8	2	6
Fisher p-value	0,515	0,0107	0,0141	0,649	0,509	0,71	0,947	0,438
FDR-adjusted p-value	0,782	0,131	0,105	1	1	1	1	1

HAL	TP53	KRAS	PIK3CA	BRAF	NRAS	PTEN	APC	RB1
Proportion in %	69	3	3	3	21	8	0	2
Fisher p-value	0,309	0,995	0,988	0,965	0,000334	0,498	1	0,952
FDR-adjusted p-value	0,629	1	1	1	0,00627	1	1	1

Mixed 2	TP53	KRAS	PIK3CA	BRAF	NRAS	PTEN	APC	RB1
Proportion in %	54	23	15	5	0	8	0	8
Fisher p-value	0,98	0,0776	0,359	0,783	1	0,668	1	0,22
FDR-adjusted p-value	0,999	0,966	0,424	1	1	1	1	1

GLSR	TP53	KRAS	PIK3CA	BRAF	NRAS	PTEN	APC	RB1
Proportion in %	60	0	4	10	4	21	0	6
Fisher p-value	0,83	1	0,94	0,414	0,781	0,185	1	0,878
FDR-adjusted p-value	0,999	1	1	1	1	0,57	1	1

Mixed 3	TP53	KRAS	PIK3CA	BRAF	NRAS	PTEN	APC	RB1
Proportion in %	52	5	2	2	8	8	5	8
Fisher p-value	0,895	0,958	0,99	1	0,526	0,931	0,612	0,48
FDR-adjusted p-value	0,999	1	1	1	1	1	1	1

ADG	TP53	KRAS	PIK3CA	BRAF	NRAS	PTEN	APC	RB1
Proportion in %	76	9	11	4	4	4	4	4
Fisher p-value	0,00281	0,73	0,436	0,936	0,882	0,977	0,743	0,926
FDR-adjusted p-value	0,139	1	1	1	1	1	1	1

SKCM	TP53	KRAS	PIK3CA	BRAF	NRAS	PTEN	APC	RB1
Proportion in %	38	0	0	75	17	12	0	0
Fisher p-value	0,998	1	1	5,01E-09	0,0725	0,674	1	1
FDR-adjusted p-value	0,999	1	1	1,48E-06	0,465	1	1	1

BRCA	TP53	KRAS	PIK3CA	BRAF	NRAS	PTEN	APC	RB1
Proportion in %	69	2	21	2	0	10	0	2
Fisher p-value	0,133	0,997	0,0175	0,981	1	0,573	1	1
FDR-adjusted p-value	0,602	1	0,105	1	1	1	1	1

Supplementary Table 3

Drug	Cluster	Effect (-Log10(IC50(M)))	95% CI (-Log10(IC50(M)))	pvalue	adjusted pvalue	Drug response relative to other cell lines
Erlotinib	ADG	1.07	(0.44 to 1.71)	3.5E-03	3.9E-02	Sensitive
Lapatinib	SCLC	-0.41	(-0.67 to -0.14)	3.6E-03	3.2E-02	Resistant
	ADG	1.12	(0.53 to 1.69)	1.5E-03	1.5E-02	Sensitive
PD0332991	GI	-0.46	(-0.71 to -0.21)	7.2E-04	7.2E-03	Resistant
	HAL	0.74	(0.38 to 1.1)	2.0E-04	2.2E-03	Sensitive
PLX4720	SKCM	1.39	(0.86 to 1.93)	1.9E-05	2.1E-04	Sensitive
PD0325901	GI	0.46	(0.15 to 0.78)	5.4E-03	4.9E-02	Sensitive
	SKCM	1.31	(0.79 to 1.82)	3.3E-05	3.6E-04	Sensitive
AZD6244	SKCM	1.25	(0.69 to 1.81)	2.3E-04	2.6E-03	Sensitive
	BRCA	-0.45	(-0.74 to -0.16)	3.4E-03	3.4E-02	Resistant

Supplementary Table 4

	CCLE vs GDSC		GSK				gCSI			
	IC50/AUC		IC50				IC50/Mean Viability			
			CCLE vs GSK		GDSC vs GSK		CCLE vs gCSI		GDSC vs gCSI	
	CCLE	GDSC	CCLE	GSK	GDSC	GSK	CCLE	gCSI	GDSC	gCSI
PACLITAXEL	276	173	118	116	70	116	164	165	65	241
LAPATINIB	277	169	118	114	70	114	165	161	64	237
ERLOTINIB	276	154					165	163	54	239
CRIZOTINIB	277	175					165	165	66	241
PD0325901	277	355					165	165	200	241
PHA665752	276	175								
TAE684	277	175								
NILOTINIB	220	369								
AZD0530	277	174								
SORAFENIB	276	172								
PD0332991	232	338								
PLX4720	273	366								
AZD6244	276	338								
NUTLIN3	277	358								
17AAG	277	366								

Supplementary Table 5

GI	Drug	Target
	drugid_17AAG	Other
	drugid_AZD6244	S/T Kinase
	drugid_PD0325901	S/T Kinase

EDOT	Drug	Target
	drugid_DOXORUBICIN	Chemotherapy

Mixed 1	Drug	Target
	drugid_GEMCITABINE	Chemotherapy
	drugid_BLEOMYCIN	Chemotherapy
	drugid_VINBLASTINE	Chemotherapy
	drugid_NVP-BEZ235	S/T Kinase, Other

HAL	Drug	Target
	drugid_IRINOTECAN	Chemotherapy
	drugid_L685458	Other
	drugid_PANOBINOSTAT	Other
	drugid_TOPOTECAN	Chemotherapy
	drugid_SUNITINIB	RTK
	drugid_CRIZOTINIB	RTK
	drugid_XMD8-85	S/T Kinase
	drugid_DMOG	Other
	drugid_PAC-1	Other
	drugid_IPA-3	S/T Kinase
	drugid_BAY-61-3606	CTK
	drugid_AICAR	S/T Kinase
	drugid_CAMPTOTHECIN-WTSI	Chemotherapy
	drugid_METHOTREXATE	Chemotherapy
	drugid_ATRA	Other
	drugid_GEFITINIB	RTK
	drugid_ABT-263	Other
	drugid_VORINOSTAT	Other
	drugid_NILOTINIB	CTK
	drugid_TEMSIROLIMUS	S/T Kinase
	drugid_AZD-2281	Other
	drugid_ABT-888	Other
	drugid_BOSUTINIB	CTK
	drugid_LENALIDOMIDE	Other
	drugid_AZD7762	S/T Kinase
	drugid_CEP-701	RTK, CTK
	drugid_VX-702	S/T Kinase
	drugid_SL-0101-1	S/T Kinase
	drugid_681640	S/T Kinase, CTK
	drugid_PD-173074	RTK
	drugid_ZM-447439	S/T Kinase
	drugid_PD0332991	S/T Kinase
	drugid_SB590885	S/T Kinase

Mixed 2	Drug	Target
	drugid_MIDOSTAURIN	RTK

GLSR	Drug	Target
	drugid_DASATINIB	CTK, RTK
	drugid_BORTEZOMIB	Other
	drugid_MIDOSTAURIN	RTK
	drugid_CHIR-99021	S/T Kinase
	drugid_AZD6482	Other
	drugid_BEXAROTENE	Other
	drugid_LFM-A13	CTK
	drugid_PAZOPANIB	RTK
	drugid_VINBLASTINE	Chemotherapy
	drugid_DOCETAXEL	Chemotherapy
	drugid_TEMSIROLIMUS	S/T Kinase
	drugid_ELESCLOMOL	Other
	drugid_BX-795	S/T Kinase
	drugid_NVP-BEZ235	S/T Kinase, Other
	drugid_GDC0941	Other

Mixed 3	Drug	Target
	drugid_VORINOSTAT	Other

ADG	Drug	Target
	drugid_AZD0530	RTK
	drugid_VANDETANIB	RTK
	drugid_ERLOTINIB	RTK
	drugid_LAPATINIB	RTK
	drugid_VINORELBINE	Chemotherapy
	drugid_BICALUTAMIDE	Other
	drugid_PF-562271	S/T Kinase
	drugid_OBATOCLAX-MESYLATE	Other
	drugid_EPOTHILONE-B	Other
	drugid_AICAR	S/T Kinase
	drugid_VINBLASTINE	Chemotherapy
	drugid_DOCETAXEL	Chemotherapy
	drugid_GEFITINIB	RTK
	drugid_BOSUTINIB	CTK
	drugid_17AAG	Other
	drugid_BIBW2992	RTK

SKCM	Drug	Target
	drugid_AZD6244	S/T Kinase
	drugid_EMBELIN	Other
	drugid_FH535	Other
	drugid_OBATOCLAX-MESYLATE	Other
	drugid_RDEA119	S/T Kinase
	drugid_CI-1040	S/T Kinase
	drugid_CEP-701	RTK, CTK
	drugid_PLX4720	S/T Kinase
	drugid_NUTLIN3	Other
	drugid_PD0325901	S/T Kinase
	drugid_SB590885	S/T Kinase
	drugid_AZD6244	S/T Kinase

BRCA	Drug	Target
	drugid_AKT-INHIBITOR-VIII	S/T Kinase
	drugid_MK-2206	S/T Kinase

Supplementary Table 6

SCLC	Drug	Target
	drugid_ERLOTINIB	RTK
	drugid_LBW242	#N/A
	drugid_PD0332991	S/T Kinase
	drugid_PHA665752	RTK
	drugid_PLX4720	S/T Kinase
	drugid_SORAFENIB	RTK
	drugid_SUNITINIB	RTK
	drugid_MG-132	Other
	drugid_CYCLOPAMINE	GPCR
	drugid_AZ628	S/T Kinase
	drugid_CRIZOTINIB	RTK
	drugid_Z-LLNLE-CHO-	Other
	drugid_DASATINIB	CTK, RTK
	drugid_WH-4-023	CTK
	drugid_BMS-536924	RTK
	drugid_JW-7-52-1	S/T Kinase
	drugid_BORTEZOMIB	Other
	drugid_XMD8-85	S/T Kinase
	drugid_LAPATINIB	RTK
	drugid_GEMCITABINE	Chemotherapy
	drugid_MIDOSTAURIN	RTK
	drugid_CHIR-99021	S/T Kinase
	drugid_AP-24534	CTK
	drugid_AZD6482	Other
	drugid_PF-562271	S/T Kinase
	drugid_FTI-277	Other
	drugid_SHIKONIN	Other
	drugid_EMBELIN	Other
	drugid_OBATOCLAX-MESYLATI	Other
	drugid_BEXAROTENE	Other
	drugid_TIPIFARNIB	Other
	drugid_RDEA119	S/T Kinase
	drugid_CI-1040	S/T Kinase
	drugid_PD0325901	S/T Kinase

GI	Drug	Target
	drugid_AP-24534	CTK
	drugid_AZD6482	Other
	drugid_DMOG	Other
	drugid_IPA-3	S/T Kinase
	drugid_PAZOPANIB	RTK
	drugid_CAMPTOTHECIN-WTSI	Chemotherapy
	drugid_VINBLASTINE	Chemotherapy
	drugid_CISPLATIN	Chemotherapy
	drugid_DOCETAXEL	Chemotherapy
	drugid_ATRA	Other
	drugid_NILOTINIB	CTK
	drugid_TEMSIROLIMUS	S/T Kinase
	drugid_AZD-2281	Other
	drugid_BOSUTINIB	CTK
	drugid_AXITINIB	RTK
	drugid_CEP-701	RTK, CTK
	drugid_AMG-706	RTK
	drugid_ELESCLOMOL	Other
	drugid_PLX4720	S/T Kinase
	drugid_BX-795	S/T Kinase
	drugid_SL-0101-1	S/T Kinase
	drugid_BI-D1870	S/T Kinase
	drugid_JNK-INHIBITOR-VIII	S/T Kinase
	drugid_681640	S/T Kinase, CTK
	drugid_PD-173074	RTK
	drugid_ZM-447439	S/T Kinase
	drugid_RO-3306	S/T Kinase
	drugid_PD0332991	S/T Kinase
	drugid_NVP-BEZ235	S/T Kinase, Other
	drugid_GDC0941	Other
	drugid_AZD8055	S/T Kinase

EDOT	Drug	Target
	drugid_L685458	#N/A
	drugid_PD0332991	S/T Kinase
	drugid_PHA665752	RTK
	drugid_PLX4720	S/T Kinase
	drugid_TKI258	RTK
	drugid_CYCLOPAMINE	GPCR
	drugid_CRIZOTINIB	RTK
	drugid_PYRIMETHAMINE	Other
	drugid_ATRA	Other
	drugid_TEMSIROLIMUS	S/T Kinase
	drugid_VX-702	S/T Kinase

Mixed 1	Drug	Target
	drugid_ERLOTINIB	RTK
	drugid_L685458	#N/A
	drugid_LBW242	#N/A
	drugid_NILOTINIB	CTK
	drugid_VANDETANIB	#N/A
	drugid_GEFITINIB	RTK
	drugid_BIBW2992	RTK

HAL	Drug	Target
	drugid_ERLOTINIB	RTK
	drugid_KIN001-135	S/T Kinase
	drugid_LAPATINIB	RTK
	drugid_NSC-87877	Other
	drugid_BICALUTAMIDE	Other
	drugid_FTI-277	Other
	drugid_FH535	Other
	drugid_BMS-754807	RTK
	drugid_BRYOSTATIN-1	S/T Kinase
	drugid_DOCETAXEL	Chemotherapy
	drugid_AZD-2281	Other
	drugid_AXITINIB	RTK
	drugid_GW-441756	RTK
	drugid_VX-702	S/T Kinase
	drugid_MK-2206	S/T Kinase

Mixed 2	Drug	Target
	drugid_AZD6244	S/T Kinase
	drugid_IRINOTECAN	Chemotherapy
	drugid_L685458	#N/A
	drugid_NILOTINIB	CTK
	drugid_PANOBINOSTAT	#N/A
	drugid_AICAR	S/T Kinase
	drugid_17AAG	Other

GLSR	Drug	Target
	drugid_AZD0530	CTK
	drugid_ERLOTINIB	RTK
	drugid_LAPATINIB	RTK
	drugid_PANOBINOSTAT	#N/A
	drugid_PLX4720	S/T Kinase
	drugid_SORAFENIB	RTK
	drugid_VANDETANIB	#N/A
	drugid_PAC-1	Other
	drugid_METHOTREXATE	Chemotherapy
	drugid_GEFITINIB	RTK

Mixed 3	Drug	Target
	drugid_ERLOTINIB	RTK
	drugid_LBW242	#N/A
	drugid_PHA665752	RTK
	drugid_PLX4720	S/T Kinase
	drugid_Z-LLNLE-CHO-	Other
	drugid_DASATINIB	CTK, RTK
	drugid_BICALUTAMIDE	Other
	drugid_BIBW2992	RTK

ADG	Drug	Target
	drugid_CRIZOTINIB	RTK
	drugid_PLX4720	S/T Kinase
	drugid_SORAFENIB	RTK
	drugid_CYCLOPAMINE	GPCR
	drugid_GSK269962A	S/T Kinase
	drugid_PAZOPANIB	RTK
	drugid_SB-216763	S/T Kinase

SKCM	Drug	Target
	drugid_AZD0530	CTK
	drugid_ERLOTINIB	RTK
	drugid_L685458	#N/A
	drugid_LAPATINIB	RTK
	drugid_PD0332991	S/T Kinase
	drugid_SORAFENIB	RTK
	drugid_TAE684	RTK
	drugid_VANDETANIB	#N/A
	drugid_WZ-1-84	CTK
	drugid_AKT-INHIBITOR-VIII	S/T Kinase
	drugid_AICAR	S/T Kinase
	drugid_GEFITINIB	RTK
	drugid_BIBW2992	RTK

BRCA	Drug	Target
	drugid_AZD6244	S/T Kinase
	drugid_PLX4720	S/T Kinase
	drugid_TOPOTECAN	Chemotherapy
	drugid_GEMCITABINE	Chemotherapy
	drugid_AP-24534	CTK
	drugid_DMOG	Other
	drugid_BAY-61-3606	CTK
	drugid_OBATOCLAX-MESYLATI	Other
	drugid_CAMPTOTHECIN-WTSI	Chemotherapy
	drugid_CYTARABINE	Chemotherapy
	drugid_METHOTREXATE	Chemotherapy
	drugid_RDEA119	S/T Kinase
	drugid_CI-1040	S/T Kinase
	drugid_AZD-2281	Other
	drugid_CEP-701	RTK, CTK
	drugid_SB-216763	S/T Kinase
	drugid_BX-795	S/T Kinase
	drugid_BI-D1870	S/T Kinase
	drugid_681640	S/T Kinase, CTK
	drugid_AZD6244	S/T Kinase

Supplementary Table 7

CCLE

Drug	Cluster	Effect (AUC)	95% CI (AUC)	pvalue	adjusted pvalue	Drug response relative to other cell lines
Erlotinib	ADG	0,05	(0,015 to 0,08)	0.0052	0.0360	Sensitive
Lapatinib	HAL	-0,04	(0,06 to -0,02)	1,00E-04	9,00E-04	Resistant
AZD6244	SKCM	0,2	(0,13 to 0,27)	5,00E-06	5,00E-05	Sensitive
	BRCA	-0,079	(-0,12 to -0,04)	2,00E-04	2,00E-03	Resistant
PLX4720	SKCM	0,16	(0,10 to 0,23)	3.1e-05	3.4e-04	Sensitive
Crizotinib	SKCM	-0,04	(-0,05 to -0,02)	3.8e-05	4.2e-04	Resistant
AZD0530	SKCM	-0,08	(-0,09 to -0,06)	2.4e-09	2.7e-08	Resistant

GDSC

Drug	Cluster	Effect (AUC)	95% CI (AUC)	pvalue	adjusted pvalue	Drug response relative to other cell lines
Erlotinib	ADG	0,09	(0,04 to 0,14)	0.003	0.0360	Sensitive
Lapatinib	HAL	-0,04	(-0,04 to -0,02)	2,39E-05	3,00E-04	Resistant
AZD6244	SKCM	0,17	(0,09 to 0,25)	3,00E-03	5,00E-05	Sensitive
	BRCA	-0,05	(-0,08 to -0,021)	0.001	0.01	Resistant
PLX4720	SKCM	0,16	(0,10 to 0,23)	1,00E-04	1,00E-03	Sensitive
Crizotinib	SKCM	-0,03	(-0,04 to -0,01)	7.9e-06	6.3e-05	Resistant
AZD0530	SKCM	-0,03	(-0,05 to -0,01)	0.002	0.020	Resistant

Supplementary Table 8

CCLE

Drug	Cluster	Effect (-Log10(IC50(M)))	95% CI (-Log10(IC50(M)))	pvalue	adjusted pvalue	Drug response relative to other cell lines
Lapatinib	Mix 1	-0,14	(-0.2 to -0.075)	0,000024	0,00024	Resistant
	SKCM	-0,13	(-0.19 to -0.071)	0,000044	0,00039	Resistant

GSK

Drug	Cluster	Effect (-Log10(IC50(M)))	95% CI (-Log10(IC50(M)))	pvalue	adjusted pvalue	Drug response relative to other cell lines
Lapatinib	Mix 1	-0,31	(-0.5 to -0.12)	0,004	0,036	Resistant
	SKCM	-0,38	(-0.52 to -0.24)	8,5E-07	0,0000093	Resistant

Supplementary Table 9

CCLE

Drug	Cluster	Effect (-Log10(IC50(M)))	95% CI (-Log10(IC50(M)))	pvalue	adjusted pvalue	Drug response relative to other cell lines
Erlotinib	Mix 1	-0,12	(-0.17 to -0.073)	0,0000016	0,000015	Resistant
	GLSR	-0,13	(-0.18 to -0.08)	0,0000014	0,000015	Resistant
	ADG	0,4	(0.13 to 0.66)	0,0053	0,032	Sensitive
Lapatinib	SKCM	-0,099	(-0.16 to -0.04)	0,0011	0,0079	Resistant
	Mix 1	-0,14	(-0.2 to -0.069)	0,00015	0,0014	Resistant
	ADG	0,27	(0.033 to 0.5)	0,027	0,19	Sensitive
PD0325901	SKCM	1,6	(1.1 to 2.1)	0,0000013	0,000012	Sensitive
	BRCA	-0,73	(-0.92 to -0.54)	9,2E-12	9,2E-11	Resistant

gCSI

Drug	Cluster	Effect (-Log10(IC50(M)))	95% CI (-Log10(IC50(M)))	pvalue	adjusted pvalue	Drug response relative to other cell
Erlotinib	Mix 1	-0,19	(-0.26 to -0.11)	0,0000049	0,000051	Resistant
	GLSR	-0,2	(-0.28 to -0.12)	0,0000046	0,000051	Resistant
	ADG	0,7	(0.36 to 1)	0,00044	0,0022	Sensitive
Lapatinib	SKCM	-0,19	(-0.27 to -0.11)	0,0000048	0,000051	Resistant
	Mix 1	-0,19	(-0.32 to -0.06)	0,0061	0,055	Resistant
	ADG	0,55	(0.34 to 0.76)	0,000023	0,00023	Sensitive
PD0325901	SKCM	2,1	(1.6 to 2.7)	1,7E-07	0,0000019	Sensitive
	BRCA	-1,1	(-1.5 to -0.74)	0,0000038	0,000038	Resistant

Supplementary Table 10

Drug	Cluster	Effect (1-Mean viability)	95% CI (1-Mean viability)	pvalue	adjusted pvalue	Drug response relative to other cell lines
Erlotinib	SCLC	-0,054	(-0.096 to -0.011)	0,025	0,15	Resistant
	Mix 1	-0,042	(-0.069 to -0.016)	0,0038	0,031	Resistant
	HAL	-0,014	(-0.051 to 0.022)	0,41	0,83	Resistant
	GLSR	-0,049	(-0.067 to -0.032)	0,0000005	0,0000055	Resistant
	ADG	0,081	(0.037 to 0.12)	0,0011	0,011	Sensitive
	SKCM	-0,039	(-0.063 to -0.014)	0,0031	0,028	Resistant
PD0325901	SKCM	0,25	(0.15 to 0.35)	0,000051	0,00051	Sensitive
	BRCA	-0,18	(-0.23 to -0.13)	8,5E-07	0,0000094	Resistant

Drug	Cluster	Effect (1-Mean viability)	95% CI 1-Mean viability)	pvalue	adjusted pvalue	Drug response relative to other cell lines
Erlotinib	SCLC	-0,042	(-0.18 to 0.094)	0,41	0,89	Resistant
	Mix 1	-0,076	(-0.1 to -0.049)	0,0000028	0,000028	Resistant
	HAL	-0,12	(-0.15 to -0.088)	3,2E-08	3,6E-07	Resistant
	GLSR	-0,041	(-0.068 to -0.015)	0,0028	0,022	Resistant
	ADG	0,12	(0.064 to 0.18)	0,00035	0,0032	Sensitive
	SKCM	-0,044	(-0.073 to -0.014)	0,0046	0,029	Resistant
PD0325901	SKCM	0,36	(0.26 to 0.46)	3,7E-07	0,000004	Sensitive
	BRCA	-0,2	(-0.29 to -0.12)	0,000064	0,00064	Resistant

<u>Supplementary data – 1 and 2</u>

Summary of each cell line cluster with information regarding tissue composition, molecular profile and drug profile in respectively CCLE and GDSC.

The first block shows the cell lines belonging to the given cluster.

The second block shows tissue composition.

<u>First window</u>: proportion of lines for the cluster originating from a given tissue.

<u>Second window</u>: proportion of the lines from a given tissue belonging to the cluster.

<u>Third window:</u> Molecular profile of the cluster.

Mutation type of each cell line. Genes and cell lines are sorted according to the number of events. The expression profile of the cluster relative to all the other cell lines for the defined gene clusters is shown. The proportion of epithelial or mesenchymal cells, according to the signature defined by Taub *et al*.

<u>The last window</u> illustrates the drug profile associated with the cluster. A volcano plot representation of *t*-test results showing the magnitude (effect; *x*-axis) and significance (-log10 (*p*-value); *y*-axis) of all drug-cluster associations in the dataset. Each circle represents a single drug-cluster interaction and its size is proportional to the number of cell lines screened. The horizontal dashed line indicates the threshold of statistical significance (-log10(0.05)). Barplot representation of the *t*-test results showing the proportion of drug types significantly associated with the cluster. List of drugs associated with the cluster (FDR < 0.05) and their phenotype.

SCLC Cluster - CCLE

Cell lines in the cluster

22RV1	DU–4475	NCI–H1618	NCI–H526
BEN	HUH–6–clone5	NCI–H1930	NCI–H727
C3A	LK–2	NCI–H2029	NCI–H810
COLO–668	LNCaP–Clone–FGC	NCI–H2081	NCI–H889
COR–L279	MFE–280	NCI–H209	SCLC–21H
COR–L51	NCI–H1048	NCI–H2141	SW620
COR–L88	NCI–H1092	NCI–H2171	TT
DMS–153	NCI–H1105	NCI–H2196	
DMS–53	NCI–H1436	NCI–H510A	
DMS–79	NCI–H146	NCI–H520	

Tissue composition

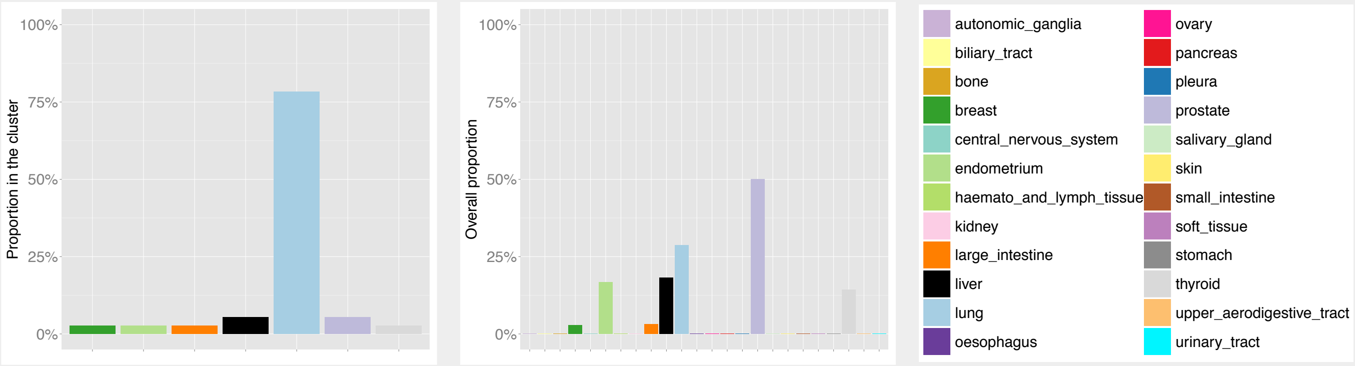

Molecular profile

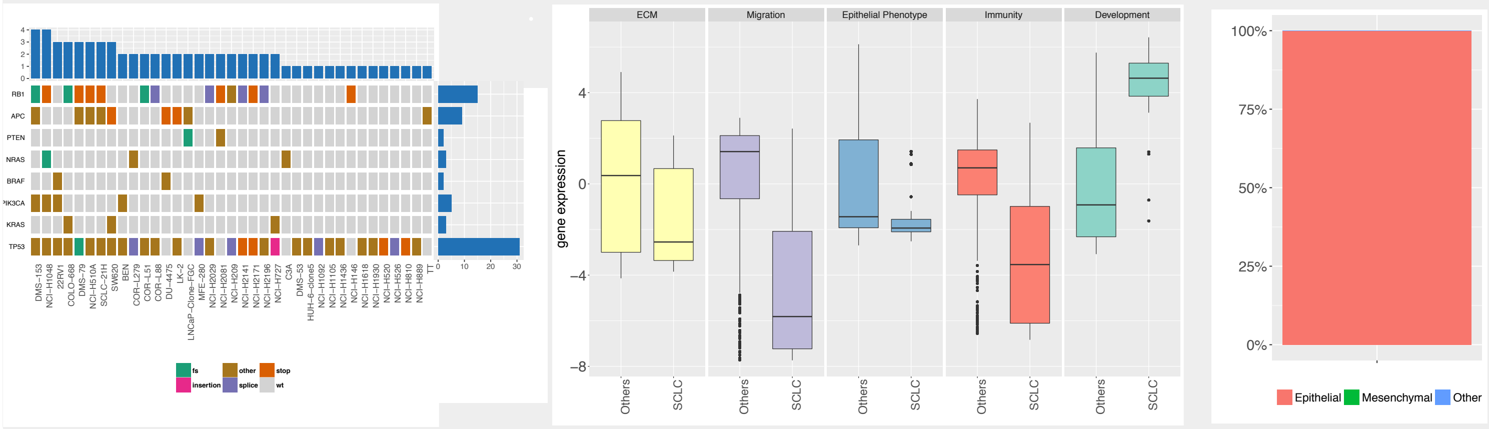

Drug profile

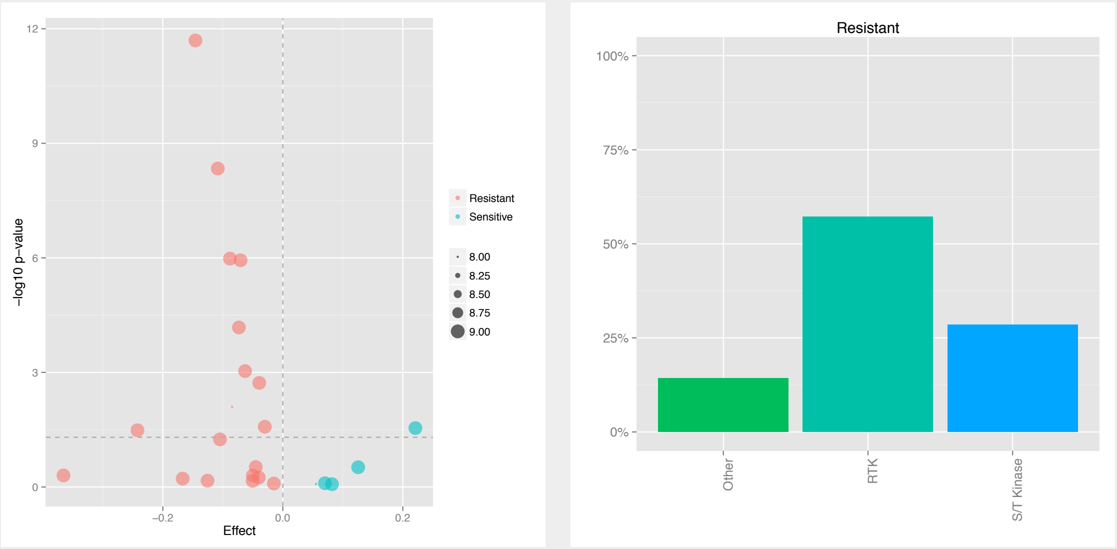

Drugs associated with the cluster (FDR$<$ **0.05**)

ERLOTINIB	Resistant
LAPATINIB	Resistant
LBW242	Resistant
PD0332991	Resistant
PHA665752	Resistant
PLX4720	Resistant
SORAFENIB	Resistant

Gastrointestinal tract Cluster - CCLE

Cell lines in the cluster

23132–87	HPAF–II	NCI–H2170	SW1417
AGS	HT–29	NCI–H508	SW1463
AsPC–1	HT55	NCI–N87	SW403
C2BBe1	KATOIII	OE19	SW48
COLO–205	KM12	RCM–1	SW837
COLO–678	LS–1034	SK–CO–1	SW948
CW–2	LS–411N	NCI–SNU–16	T84
DV–90	LS–513	NCI–SNU–5	TGBC11TKB
HCT–116	LoVo	SNU–C1	
HCT–15	MKN45	SW1116	

Tissue composition

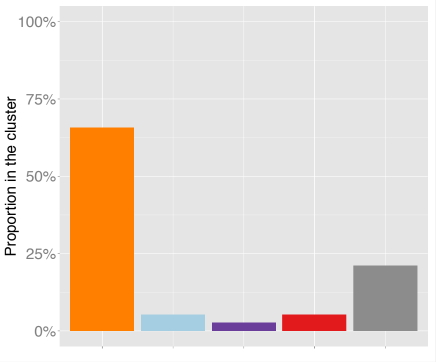

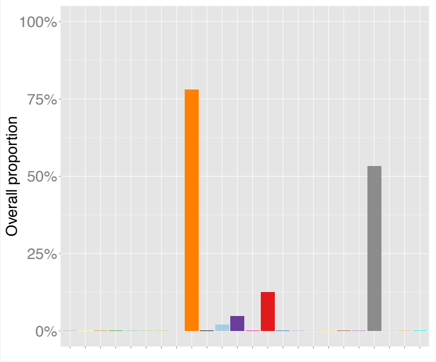

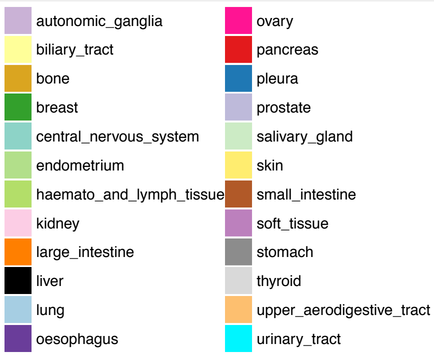

Molecular profile

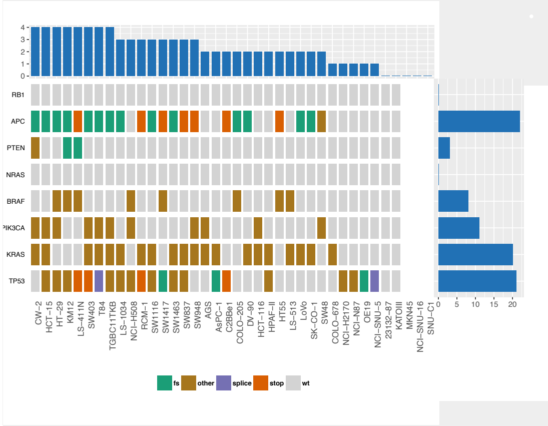

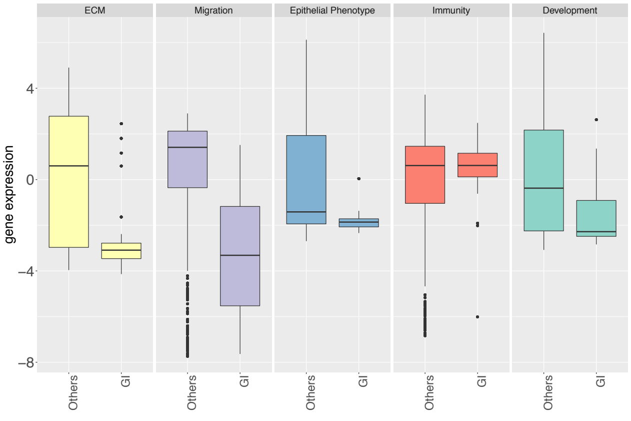

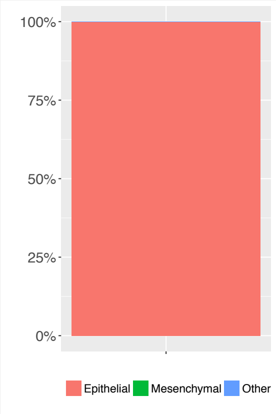

Drug profile

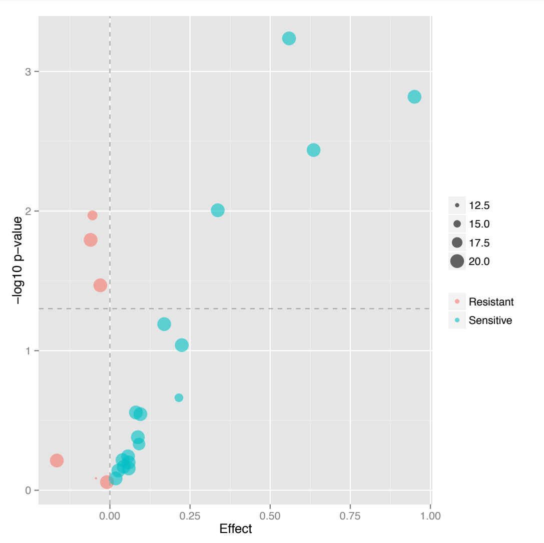

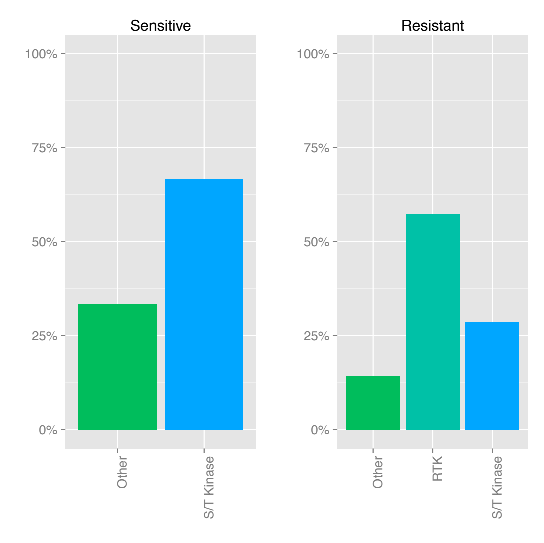

Drugs associated with the cluster (FDR$<$ **0.05**)

17AAG	Sensible
AZD6244	Sensible
PD0325901	Sensible

Endodermal origin Cluster - CCLE

Cell lines in the cluster

5637	Capan–2	LCLC–97TM1	NCI–H2347	PANC–08–13
647–V	DU–145	LS–123	NCI–H358	PANC–10–05
CAL–12T	EPLC–272H	MKN7	NCI–H441	SW900
CAL–85–1	HCC1143	NCI–H1650	NCI–H596	TE–1
CFPAC–1	HCC38	NCI–H1693	NCI–H747	YAPC
COLO–680N	HSC–3	NCI–H1838	NUGC–3	
COR–L23	HT–1197	NCI–H1975	OE33	
Calu–3	HuP–T3	NCI–H2009	OVCAR–4	
Caov–3	HuP–T4	NCI–H2087	PC–3	
CAPAN–1	HuCCT1	NCI–H2291	PANC–03–27	

Tissue composition

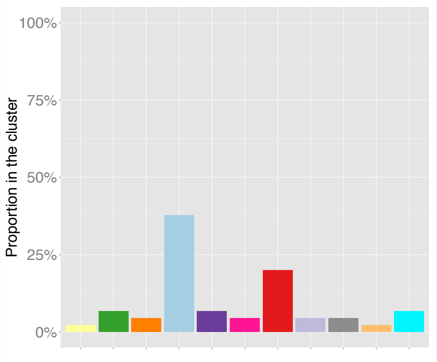

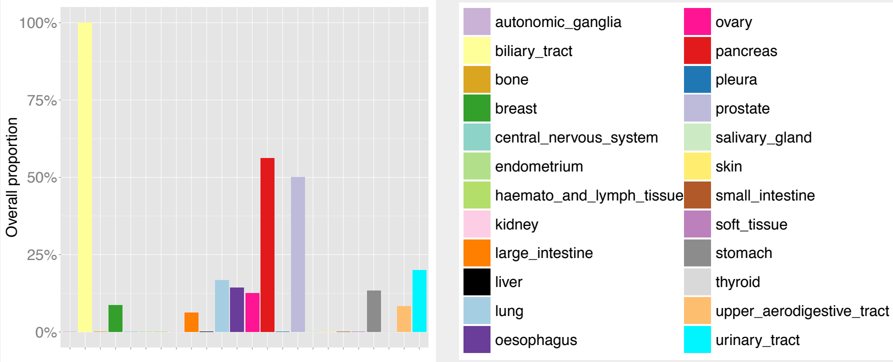

Molecular profile

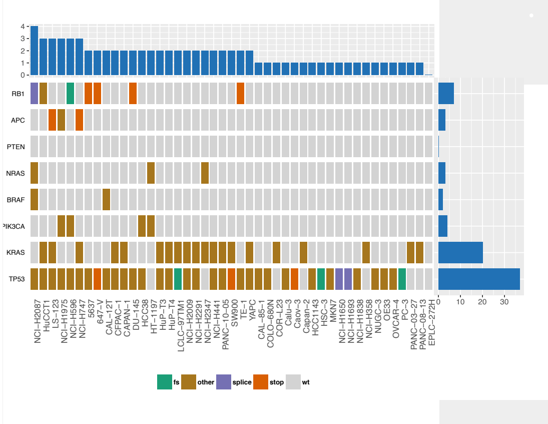

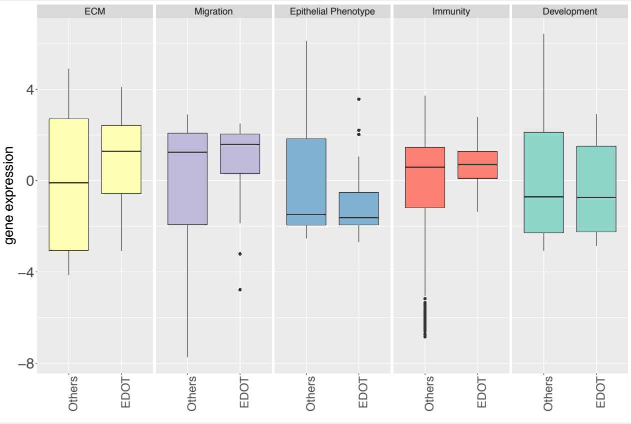

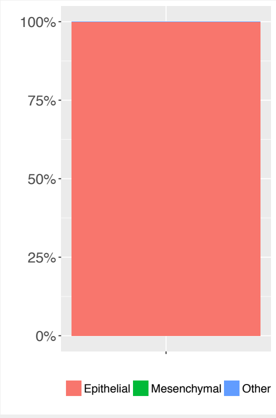

Drug profile

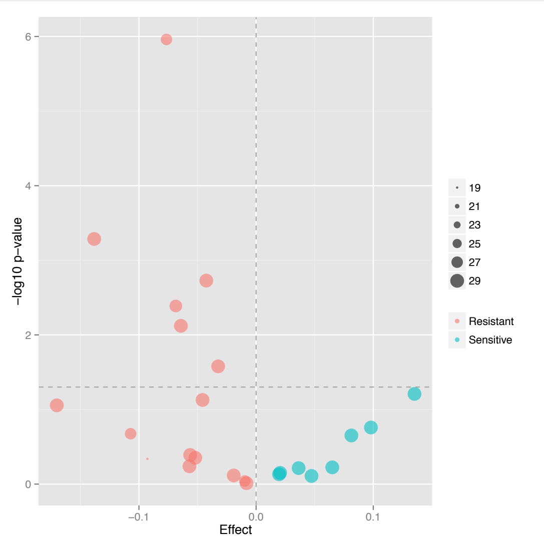

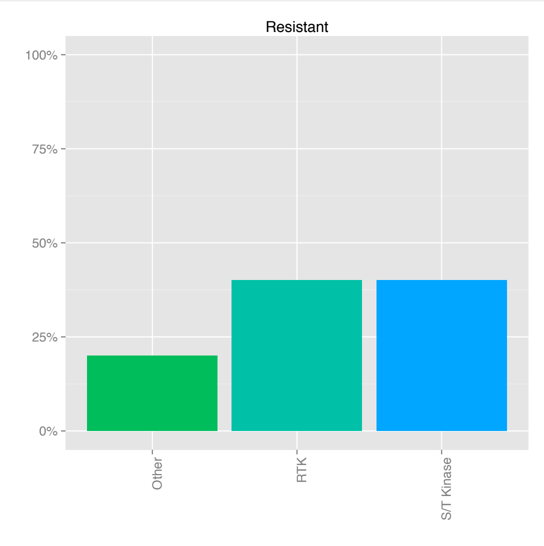

Drugs associated with the cluster (FDR< 0.05)

L685458	Resistant
PD0332991	Resistant
PHA665752	Resistant
PLX4720	Resistant
TKI258	Resistant

Mixed 1 Cluster - CCLE

Cell lines in the cluster

639–V	LU–99A	TYK–nu
8505C	MSTO–211H	UM–UC–3
AM–38	NCI–H1299	
CAL–120	NCI–H2052	
CAL–62	NCI–H23	
HD–MY–Z	NCI–H650	
HLE	OVCAR–8	
IA–LM	SK–HEP–1	
KP–4	SW1573	
LOXIMVI	T–24	

Tissue composition

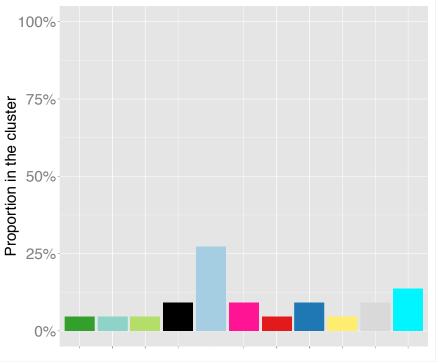

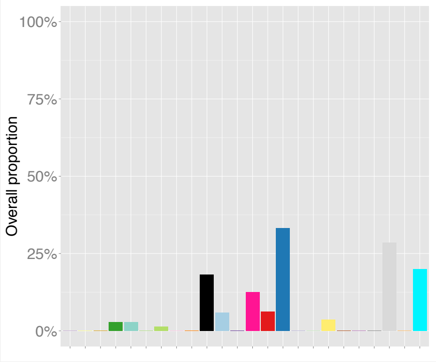

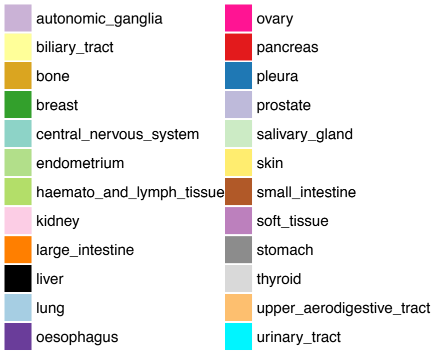

Molecular profile

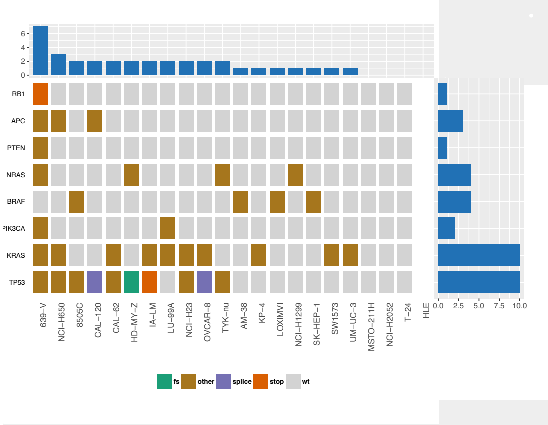

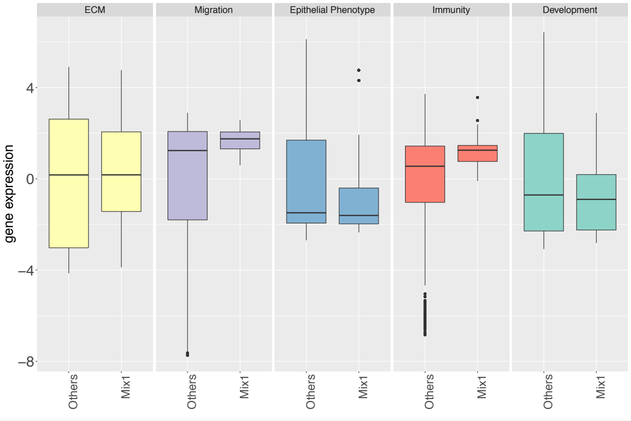

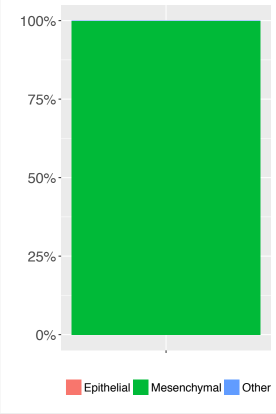

Drug profile

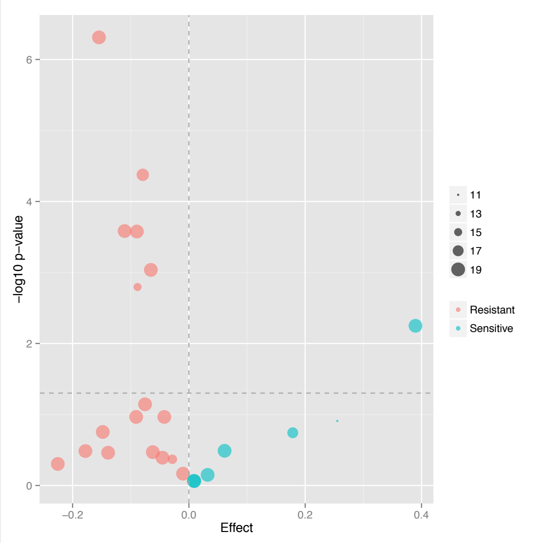

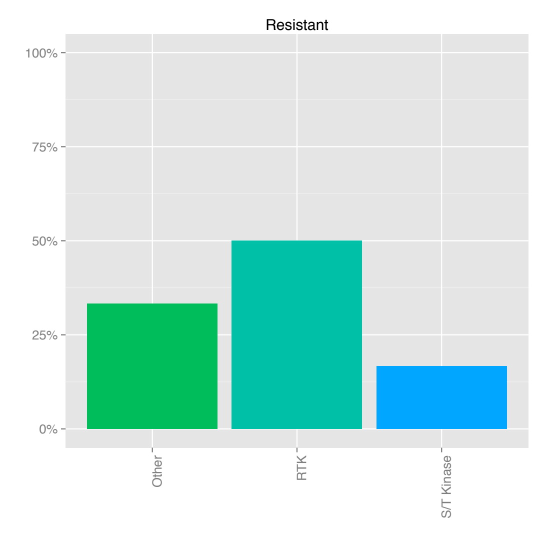

Drugs associated with the cluster (FDR$<$ $\mathbf{0.05}$)

ERLOTINIB	Resistant
L685458	Resistant
LAPATINIB	Resistant
LBW242	Resistant
NILOTINIB	Resistant
VANDETANIB	Resistant

Haematopoietic and Lymphoid tissue Cluster - CCLE

Cell lines in the cluster

697	DEL	HL–60	KMS–12–BM	MHH–CALL–4	OCI–AML2	SK–MM–2
A3–KAW	DOHH–2	HT	K052	MJ	OPM–2	SKM–1
AML–193	Daudi	HuNS1	L–363	MOLT–13	P12–ICHIKAWA	SR
BL–41	EB2	JVM–2	L–428	MOLT–16	P31–FUJ	ST486
BL–70	EHEB	JVM–3	L–540	MOLT–4	PF–382	SU–DHL–1
BV–173	EM–2	KARPAS–299	LAMA–84	MONO–MAC–6	RL	SUP–T1
CA46	GDM–1	KARPAS–422	LP–1	NALM–1	RPMI–8226	TALL–1
CMK	HDLM–2	KASUMI–1	LOUCY	NALM–6	RPMI–8402	THP–1
CML–T1	HEL	KE–37	MEG–01	NB4	RS4–11	U–266
DB	HH	KM–H2	MHH–CALL–2	NOMO–1	Raji	

Tissue composition

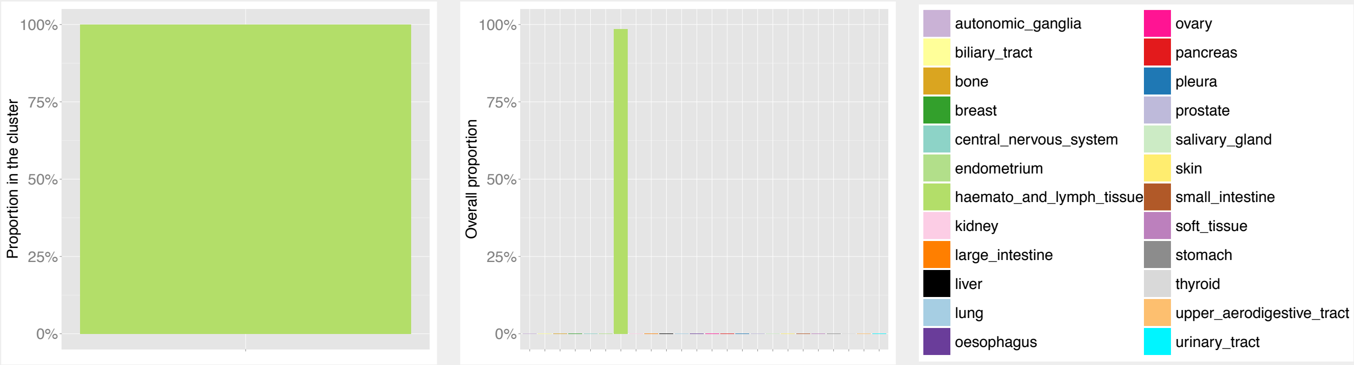

Molecular profile

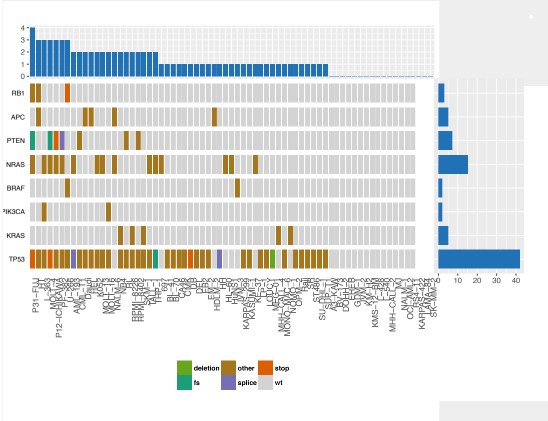

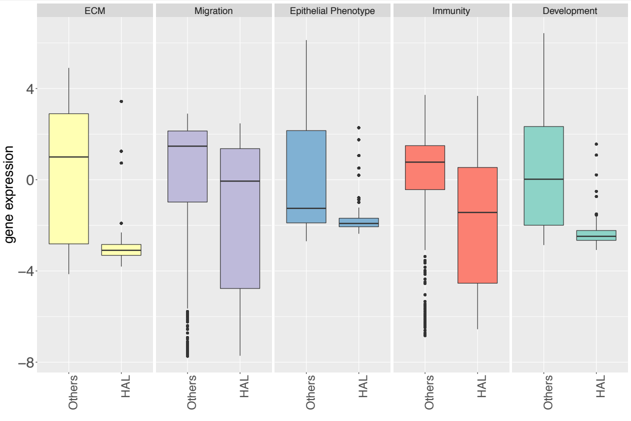

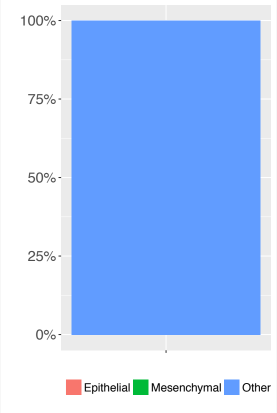

Drug profile

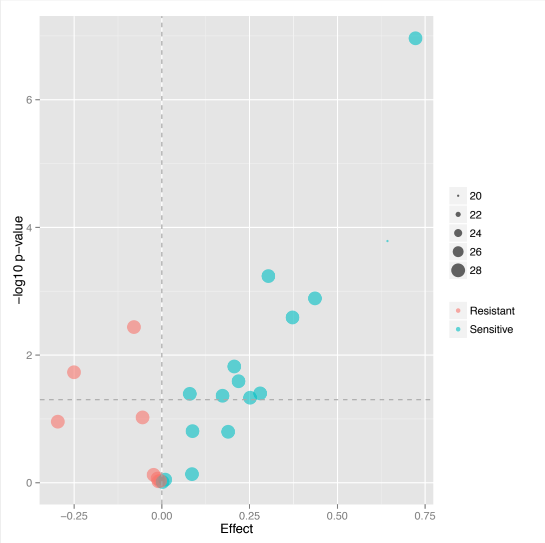

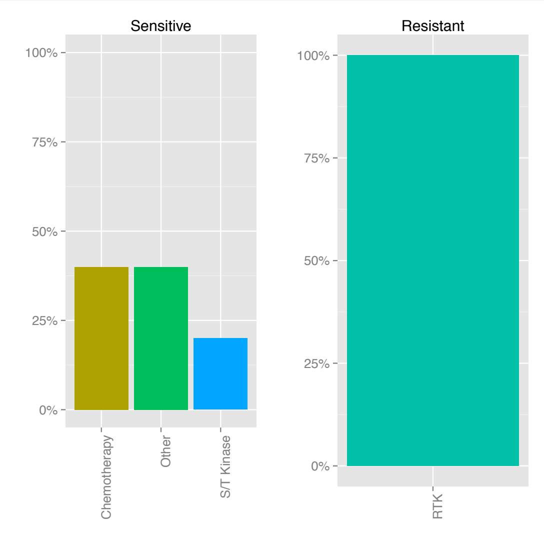

Drugs associated with the cluster (FDR$<$ **0.05**)

IRINOTECAN	Sensible
L685458	Sensible
PANOBINOSTAT	Sensible
PD0332991	Sensible
TOPOTECAN	Sensible
ERLOTINIB	Resistant

Mixed 2 Cluster - CCLE

Cell lines in the cluster

769–P	Calu–6	MKN1	NCI–H28	RERF–LC–MS
786–0	EFO–21	NCI–H1355	NCI–H460	RMG–I
A704	GCIY	NCI–H1563	NCI–H716	SK–MES–1
A549	HEC–1	NCI–H1651	NCI–H838	SK–OV–3
ACHN	HuH–7	NCI–H1755	OAW–42	SNG–M
BHT–101	IGROV–1	NCI–H1792	OC–314	SNU–449
CAL–51	KURAMOCHI	NCI–H1793	OS–RC–2	SW1990
CAL–54	LU–65	NCI–H2030	PLC–PRF–5	SW1710
COR–L105	LXF–289	NCI–H2228	PSN1	TCCSUP
Calu–1	MDA–MB–231	NCI–H2405	RCC10RGB	VMRC–RCZ

Tissue composition

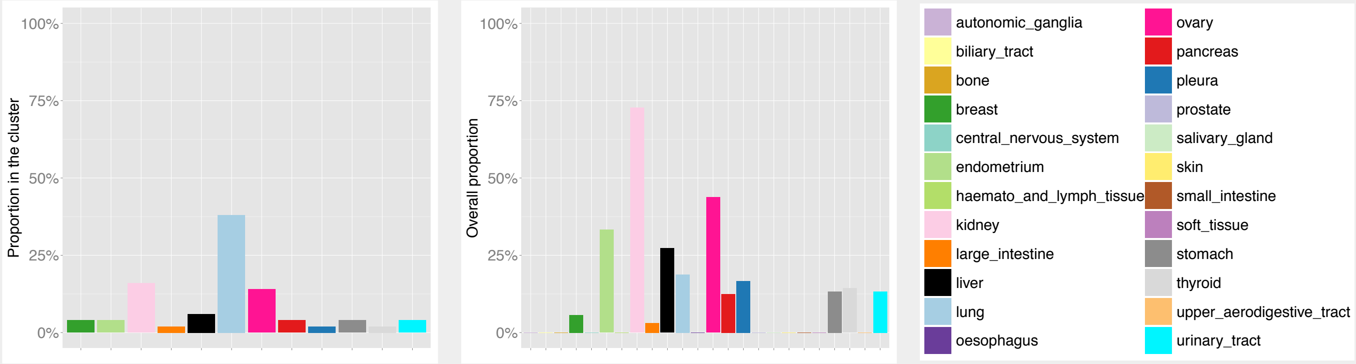

Molecular profile

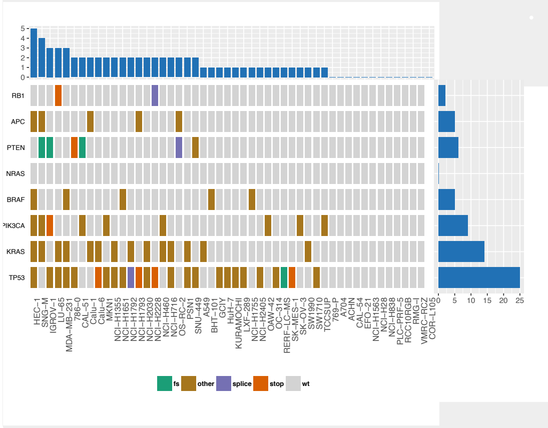

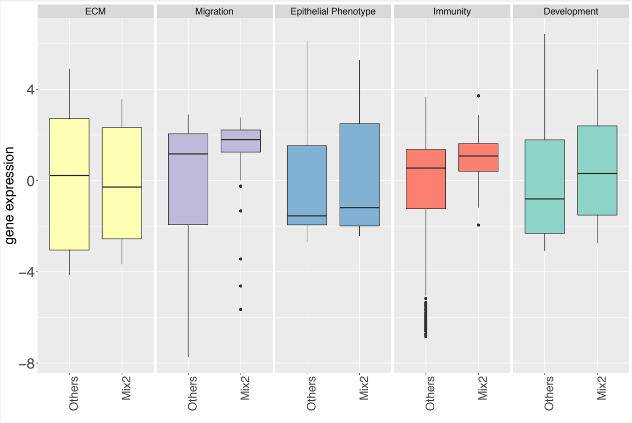

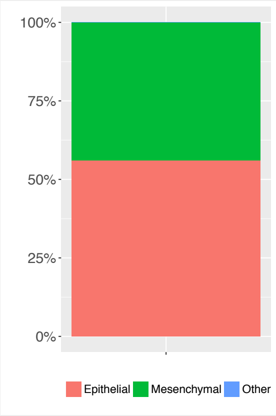

Drug profile

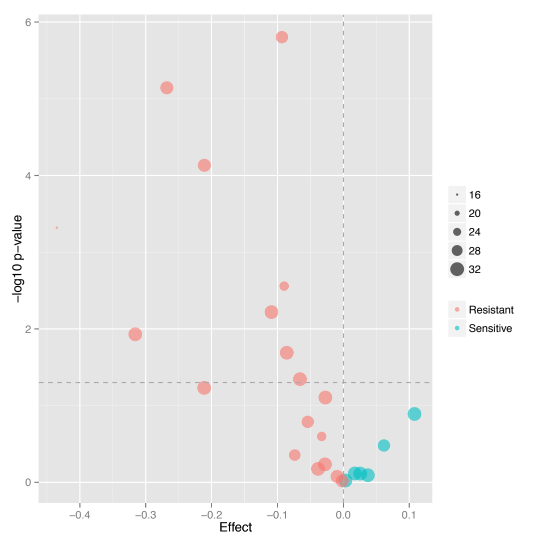

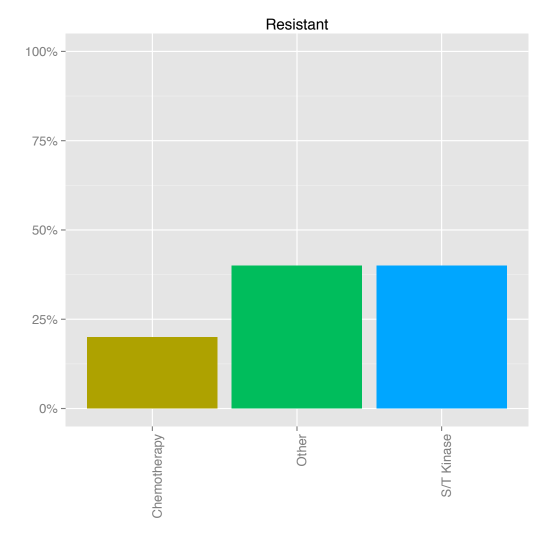

Drugs associated with the cluster (FDR< 0.05)

AZD6244	Resistant
IRINOTECAN	Resistant
L685458	Resistant
NILOTINIB	Resistant
PANOBINOSTAT	Resistant

Glioma and Sarcoma Cluster - CCLE

Cell lines in the cluster

8–MG–BA	CHP–212	H4	KNS–81–FD	NCI–H2452	SJSA–1	Saos–2
A498	DBTRG–05MG	HCC1395	KP–N–S19s	NH–6	SK–LMS–1	TI–73
A172	DK–MG	HOS	KS–1	NMC–G1	SK–LU–1	T98G
BCPAP	Daoy	HT–1080	LCLC–103H	ONS–76	SK–N–AS	TE–8
BFTC–909	ESS–1	Hs–578–T	MDA–MB–157	RD	SNB19	U–118–MG
BT–549	FTC–133	HuH–28	MG–63	RH–18	SNU–387	U–2–OS
Becker	GAMG	IST–MES1	MOG–G–CCM	RPMI–7951	SNU–423	U251
CAS–1	GCT	J82	MOG–G–UVW	S–117	SNU–475	U–87–MG
CCF–STTG1	GI–1	KALS–1	MPP–89	SF295	SW1088	YH–13
CGTH–W–1	GMS–10	KNS–42	NCI–H226	SF126	SW1783	YKG–1

Tissue composition

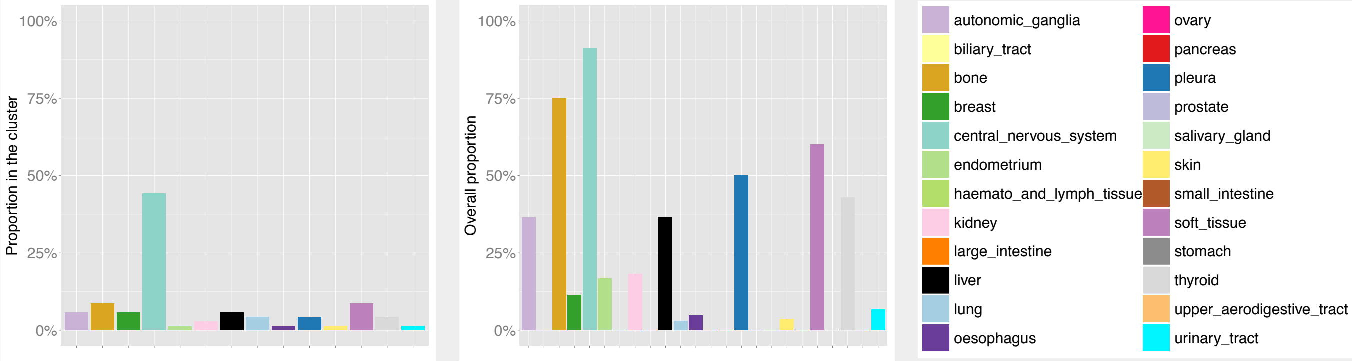

Molecular profile

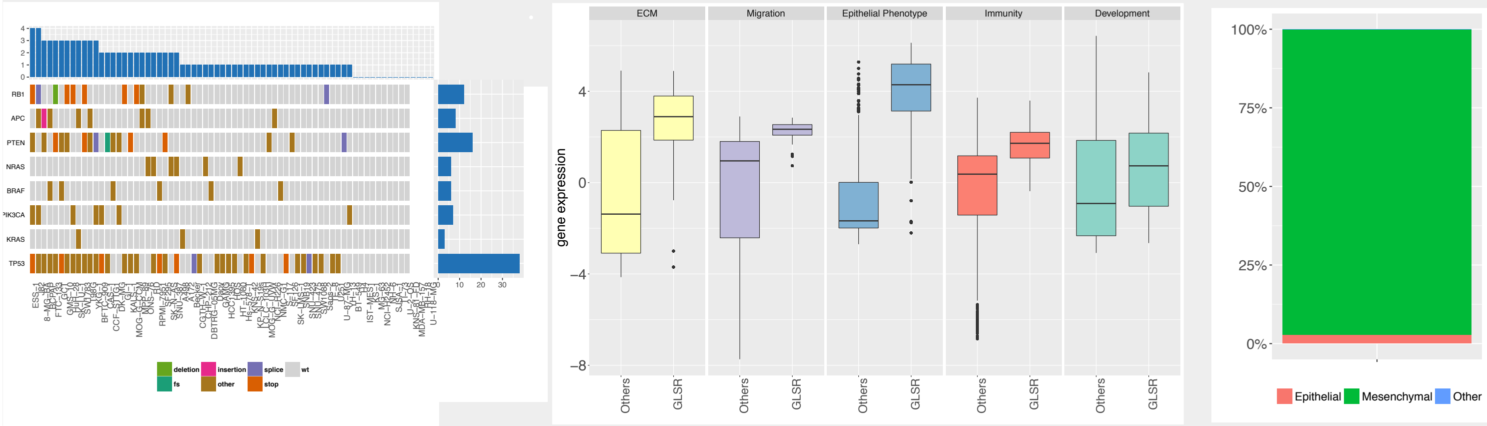

Drug profile

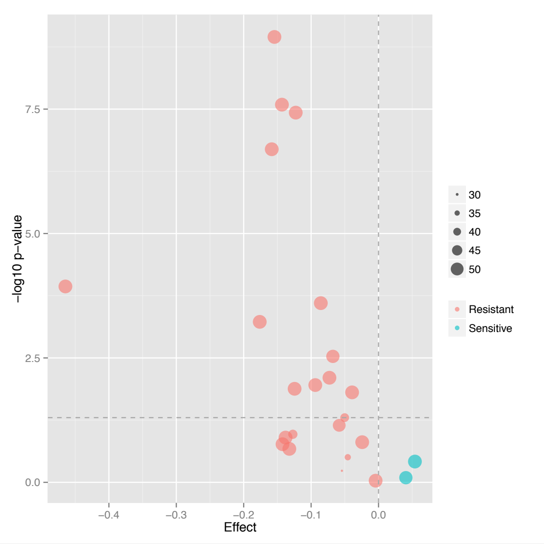

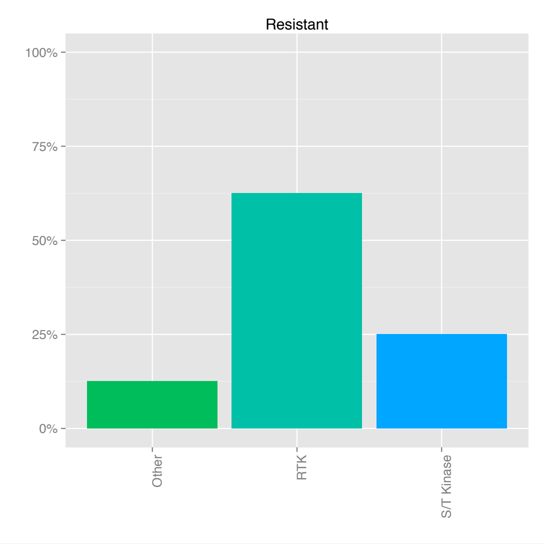

Drugs associated with the cluster (FDR< 0.05)

AZD0530	Resistant
ERLOTINIB	Resistant
LAPATINIB	Resistant
PANOBINOSTAT	Resistant
PD0325901	Resistant
PLX4720	Resistant
SORAFENIB	Resistant
VANDETANIB	Resistant

Mixed 3 Cluster - CCLE

Cell lines in the cluster

A204	ECC12	MIA–PaCa–2	RKO
A673	G–401	NB1	SBC–5
A2780	HGC–27	NCI–H1155	SHP–77
AN3–CA	HUTU–80	NCI–H1703	SIMA
CHL–1	KP–N–RT–BM–1	NCI–H2227	SJRH30
COLO–320–HSR	KP–N–YN	NCI–H446	SK–N–DZ
CPC–N	MES–SA	NCI–H522	SK–N–FI
D–283MED	MFE–296	NCI–H524	SK–UT–1
DMS–114	MHH–ES–1	NCI–H661	NCI–SNU–1
DMS–273	MHH–NB–11	NCI–H82	

Tissue composition

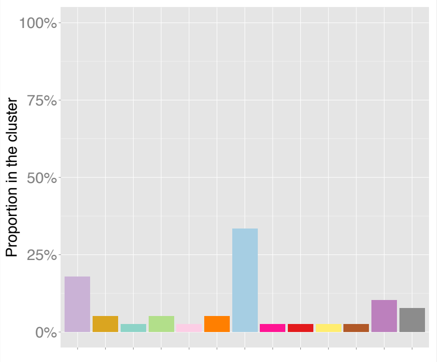

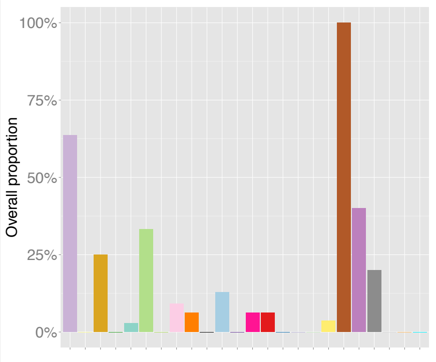

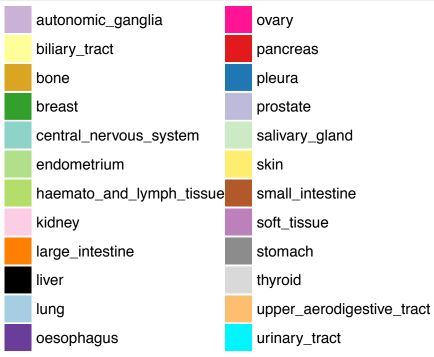

Molecular profile

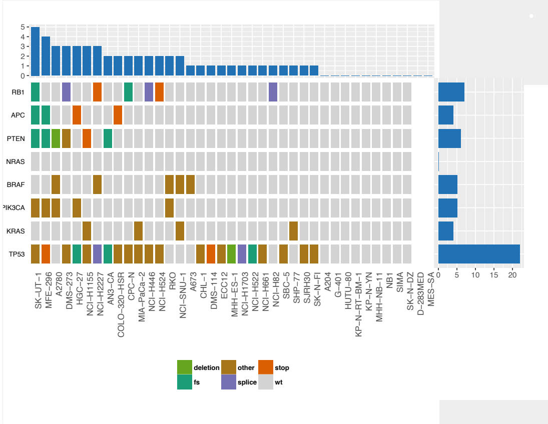

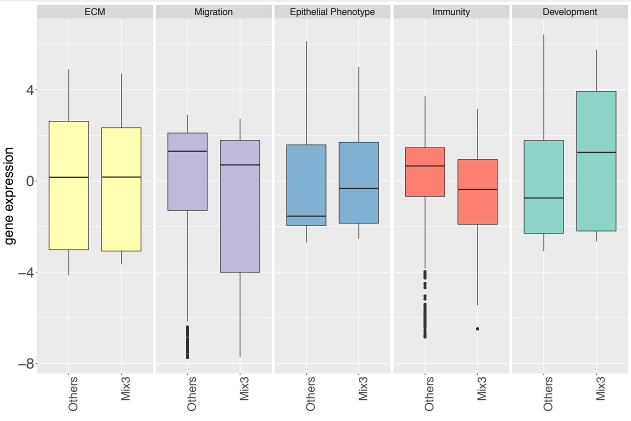

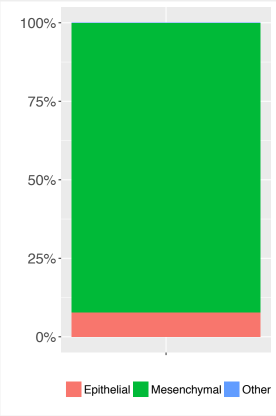

Drug profile

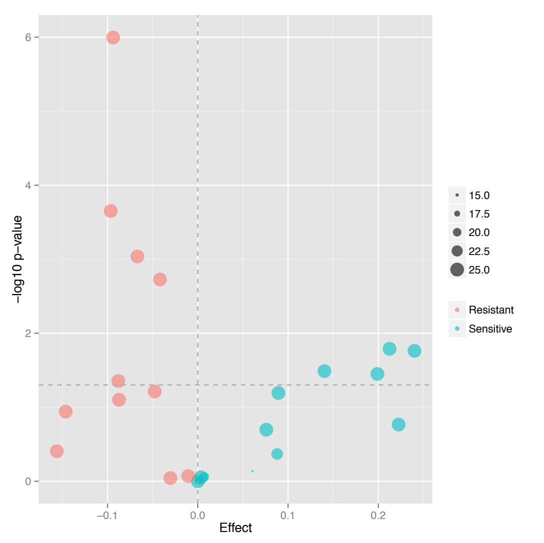

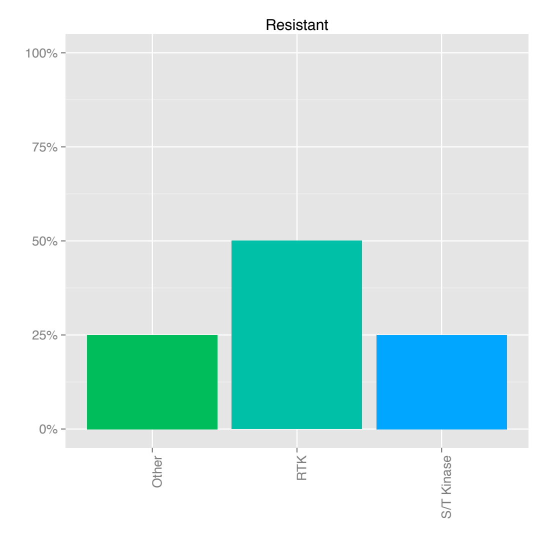

Drugs associated with the cluster (FDR< 0.05)

ERLOTINIB	Resistant
LBW242	Resistant
PHA665752	Resistant
PLX4720	Resistant

Aerodigestive tract Cluster - CCLE

Cell lines in the cluster

A253	HCC1806	KYSE–450	SCC–25	VM–CUB–1
BFTC–905	HSC–2	KYSE–510	SCC–4	
BHY	HSC–4	KYSE–520	SCC–9	
BxPC–3	KNS–62	KYSE–70	SW780	
CAL–27	KU–19–19	LC–1F	TE–10	
CAL–33	KYSE–140	NCI–H1666	TE–11	
Detroit562	KYSE–150	NCI–H292	TE–15	
EC–GI–10	KYSE–180	PC–14	TE–5	
EFO–27	KYSE–270	RT–112	TE–6	
FADU	KYSE–410	SCC–15	TE–9	

Tissue composition

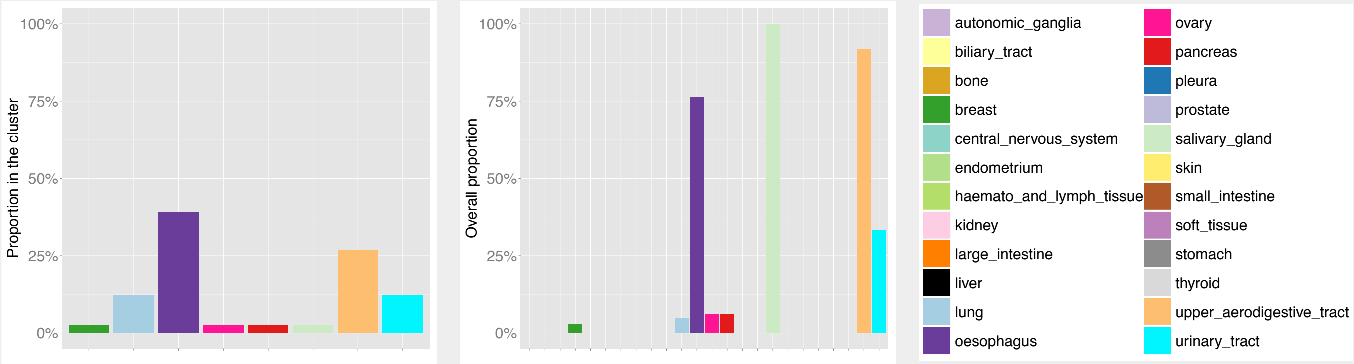

Molecular profile

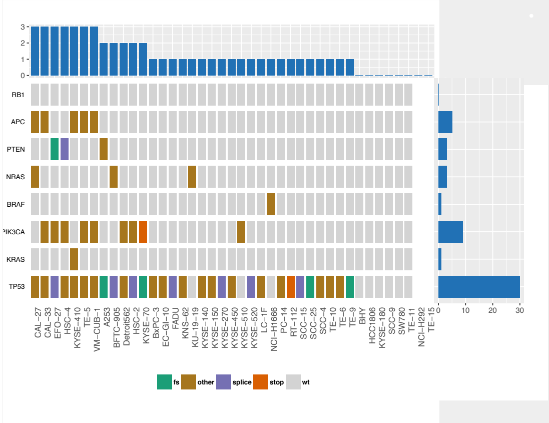

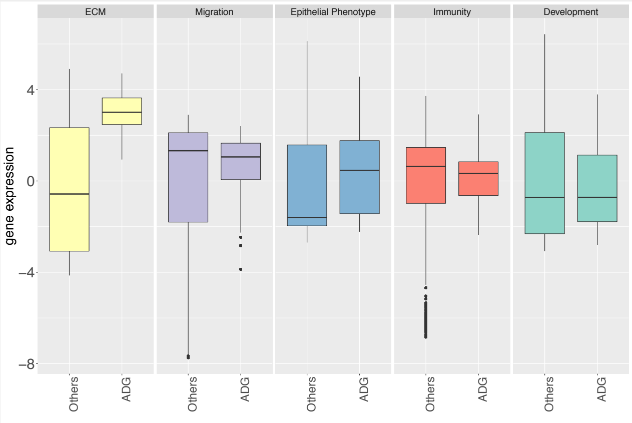

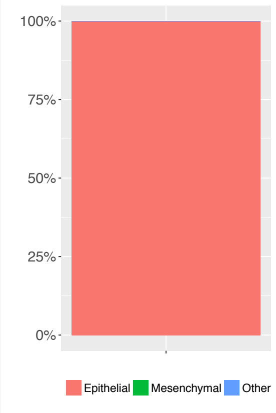

Drug profile

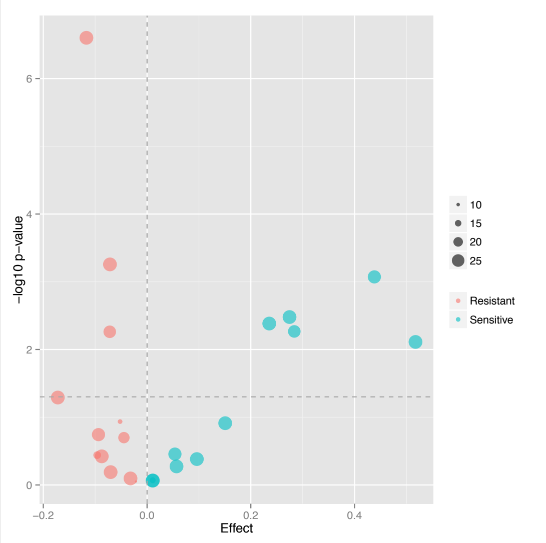

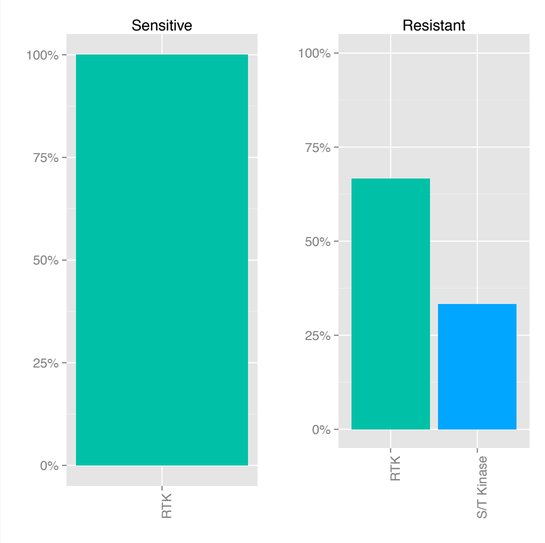

Drugs associated with the cluster (FDR$<$ **0.05**)

AZD0530	Sensible
ERLOTINIB	Sensible
LAPATINIB	Sensible
VANDETANIB	Sensible
CRIZOTINIB	Resistant
PLX4720	Resistant
SORAFENIB	Resistant

Skin Cluster - CCLE

Cell lines in the cluster

A375	IGR–1	SK–MEL–3
A101D	IPC–298	SK–MEL–30
A2058	MEL–HO	SK–MEL–5
C32	MEL–JUSO	UACC–257
COLO–741	Malme–3M	UACC–62
COLO–792	Mewo	WM–115
COLO–679	RVH–421	
COLO–800	SK–MEL–2	
G–361	SK–MEL–24	
HT–144	SK–MEL–28	

Tissue composition

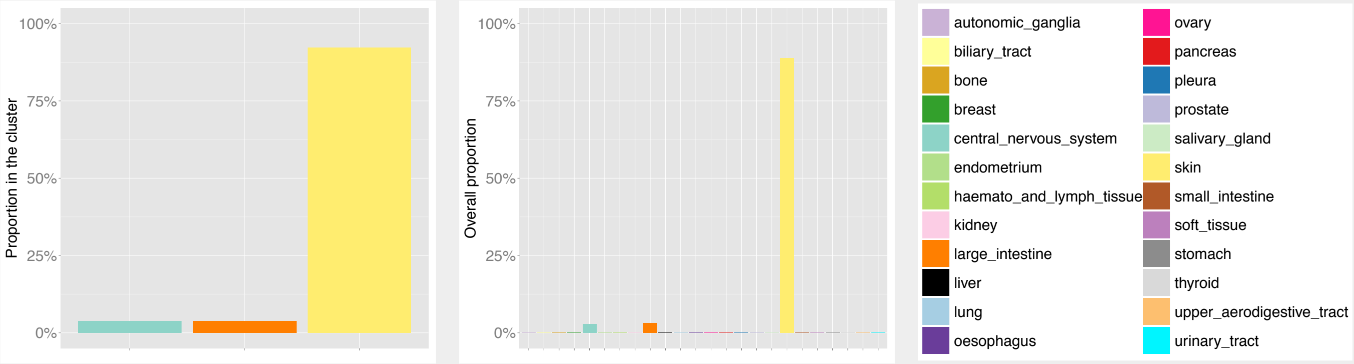

Molecular profile

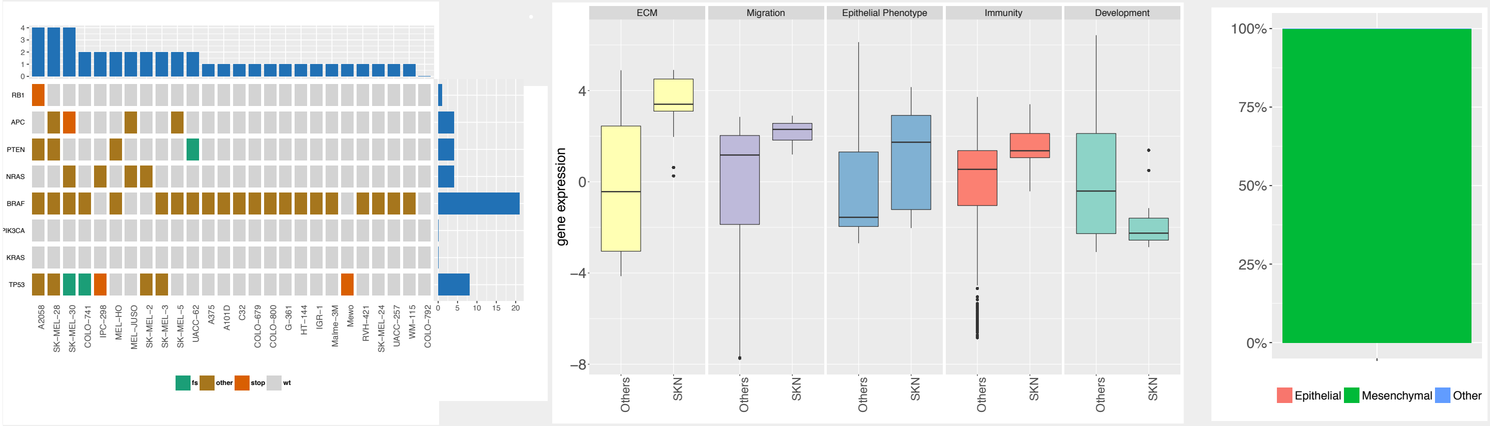

Drug profile

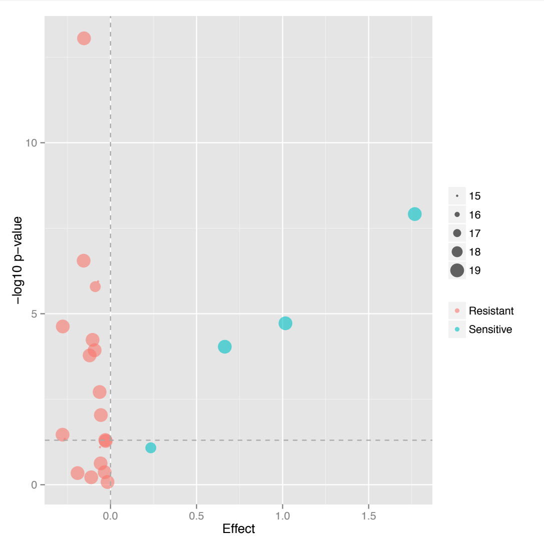

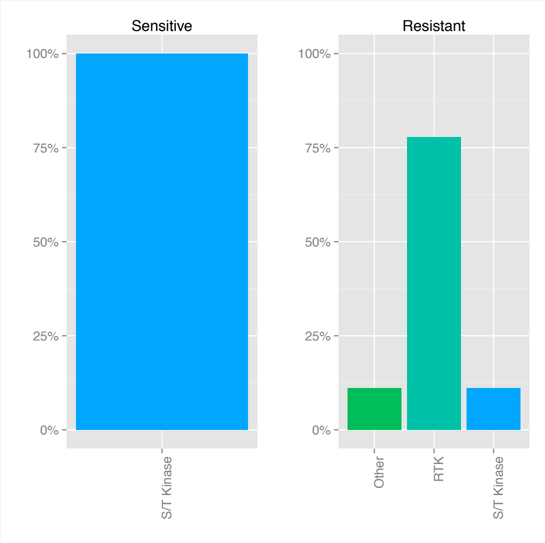

Drugs associated with the cluster (FDR< 0.05)

AZD6244	Sensible
PD0325901	Sensible
PLX4720	Sensible
AZD0530	Resistant
CRIZOTINIB	Resistant
ERLOTINIB	Resistant
L685458	Resistant
LAPATINIB	Resistant
PD0332991	Resistant
SORAFENIB	Resistant
TAE684	Resistant
VANDETANIB	Resistant

Breast Cluster - CCLE

Cell lines in the cluster

ABC–1	HCC1599	MDA–MB–361	OAW–28
BT–20	HCC1937	MDA–MB–415	T47D
CAL–148	HCC1954	MDA–MB–453	UACC–812
CAMA–1	HCC2157	MDA–MB–468	ZR–75–30
Caov–4	HCC2218	NCI–H1395	
ChaGo–K–1	HCC70	NCI–H1437	
EFM–19	HT–1376	NCI–H1573	
EVSA–T	MCF7	NCI–H2126	
HCC1187	MDA–MB–134–VI	NCI–H2342	
HCC1419	MDA–MB–175–VII	OVCAR–3	

Tissue composition

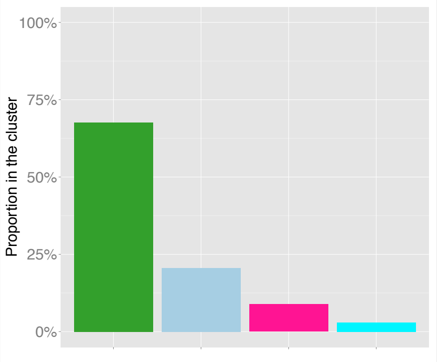

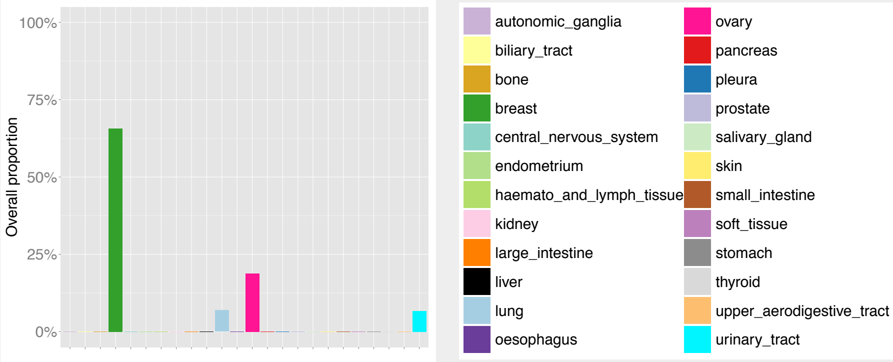

Molecular profile

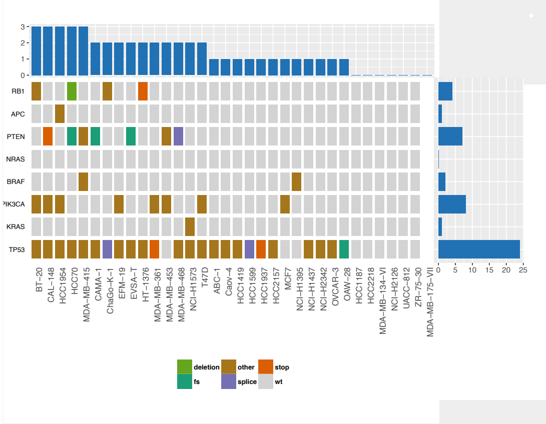

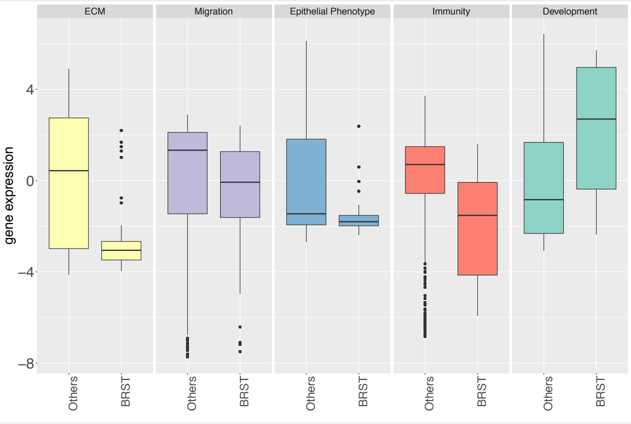

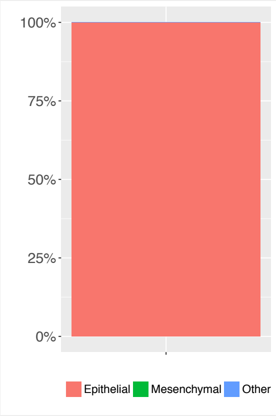

Drug profile

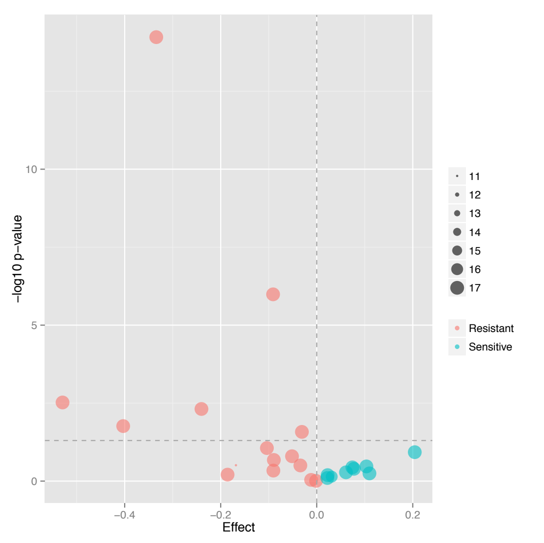

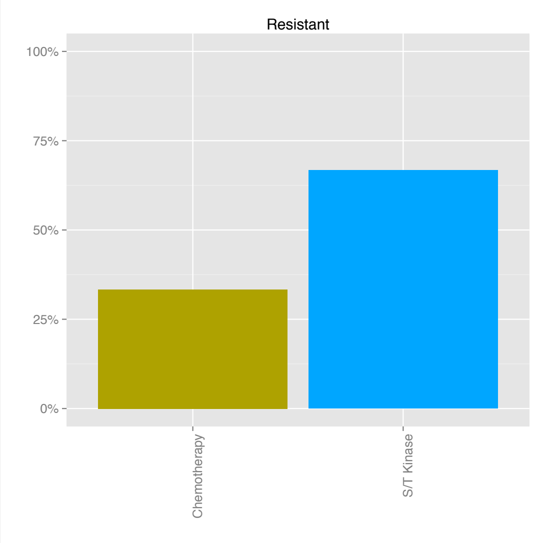

Drugs associated with the cluster (FDR< 0.05)

AZD6244	Resistant
PLX4720	Resistant
TOPOTECAN	Resistant

SCLC Cluster - GDSC

Cell lines in the cluster

22RV1	DMS–79	NCI–H146	NCI–H520
BEN	HUH–6–clone5	NCI–H1618	NCI–H526
C2BBe1	HuH–7	NCI–H1930	NCI–H727
C3A	LK–2	NCI–H2029	NCI–H810
COLO–668	LNCaP–Clone–FGC	NCI–H2081	NCI–H889
COR–L279	MDA–MB–134–VI	NCI–H209	SCLC–21H
COR–L51	NCI–H1048	NCI–H2141	TT
COR–L88	NCI–H1092	NCI–H2171	
DMS–153	NCI–H1105	NCI–H2196	
DMS–53	NCI–H1436	NCI–H510A	

Tissue composition

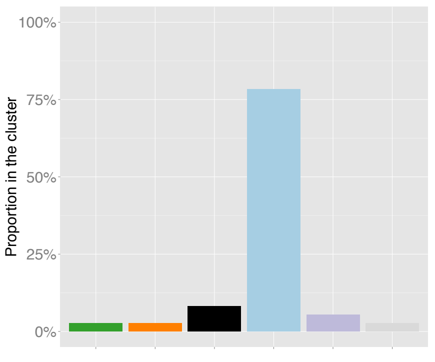

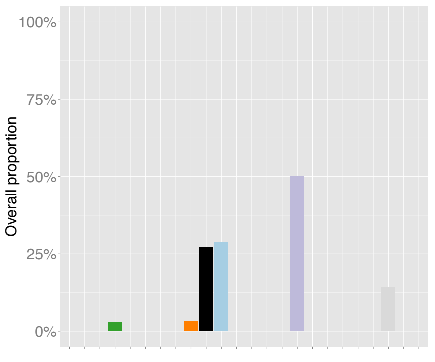

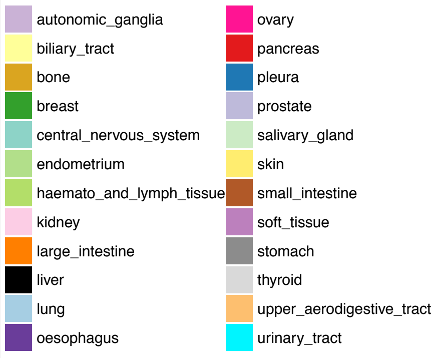

Molecular profile

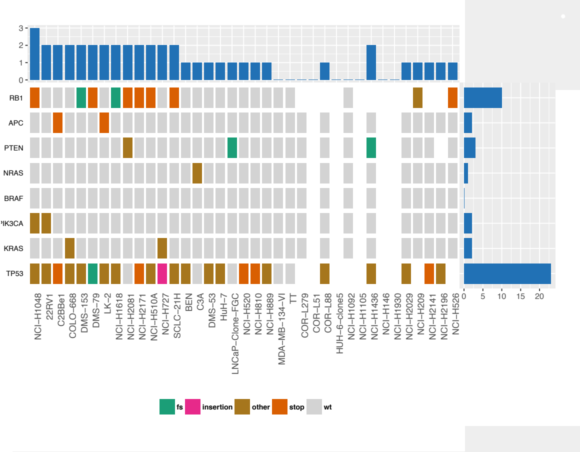

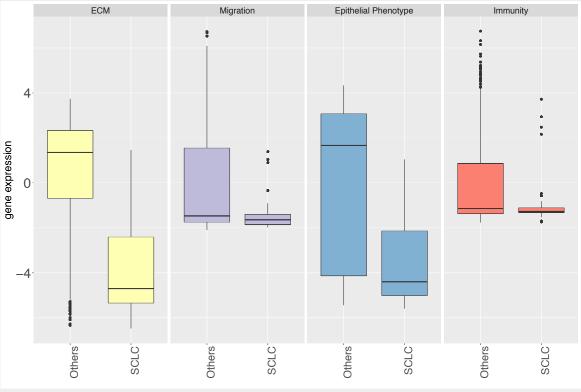

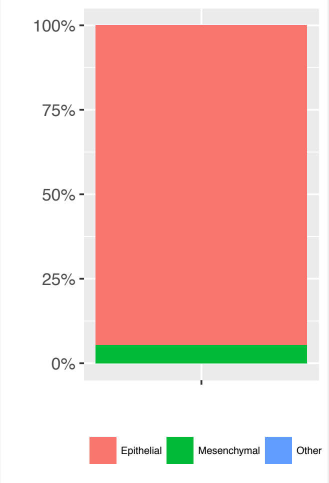

Drug profile

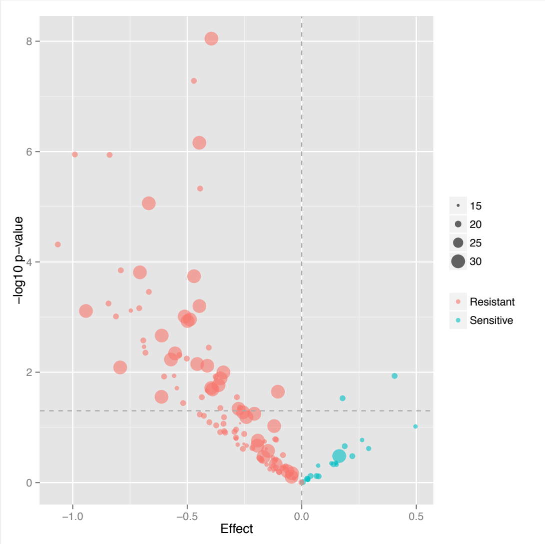

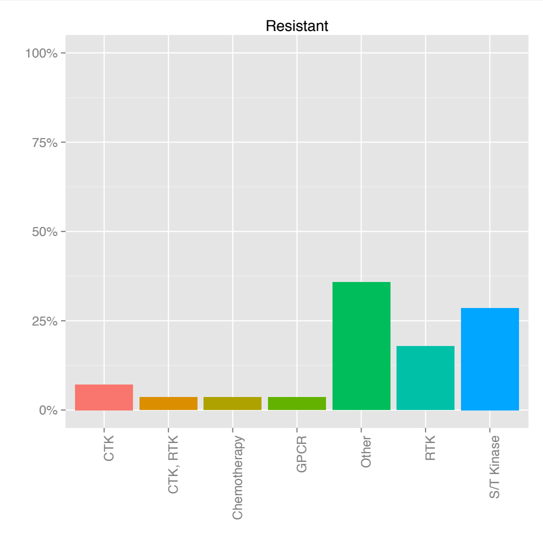

Drugs associated with the cluster (FDR< 0.05)

SUNITINIB	Resistant
MG–132	Resistant
CYCLOPAMINE	Resistant
AZ628	Resistant
CRIZOTINIB	Resistant
Z–LLNLE–CHO–	Resistant
DASATINIB	Resistant
WH–4–023	Resistant
BMS–536924	Resistant
JW–7–52–1	Resistant
BORTEZOMIB	Resistant
XMD8–85	Resistant
LAPATINIB	Resistant
GEMCITABINE	Resistant
MIDOSTAURIN	Resistant
CHIR–99021	Resistant
AP–24534	Resistant
AZD6482	Resistant
PF–562271	Resistant
FTI–277	Resistant
SHIKONIN	Resistant
EMBELIN	Resistant
OBATOCLAX–MESYLATE	Resistant
BEXAROTENE	Resistant
TIPIFARNIB	Resistant
RDEA119	Resistant
CI–1040	Resistant
PD0325901	Resistant

Gastrointestinal tract Cluster - GDSC

Cell lines in the cluster

23132–87	HPAF–II	MKN45	SNU–C1
AGS	HT–29	NCI–H508	SW1116
CFPAC–1	HT55	NCI–H747	SW1417
COLO–205	HuP–T4	NCI–N87	SW1463
COLO–678	KATOIII	OE19	SW837
CW–2	KM12	OE33	SW948
CAPAN–1	LS–1034	PANC–08–13	T84
Capan–2	LS–411N	RCM–1	TGBC11TKB
DV–90	LS–513	SK–CO–1	YAPC
HCT–15	LoVo	NCI–SNU–16	

Tissue composition

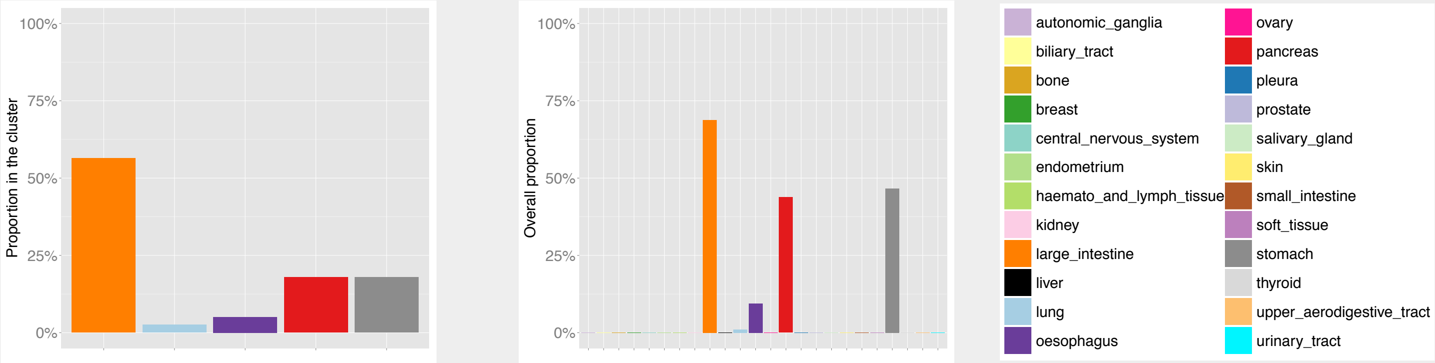

Molecular profile

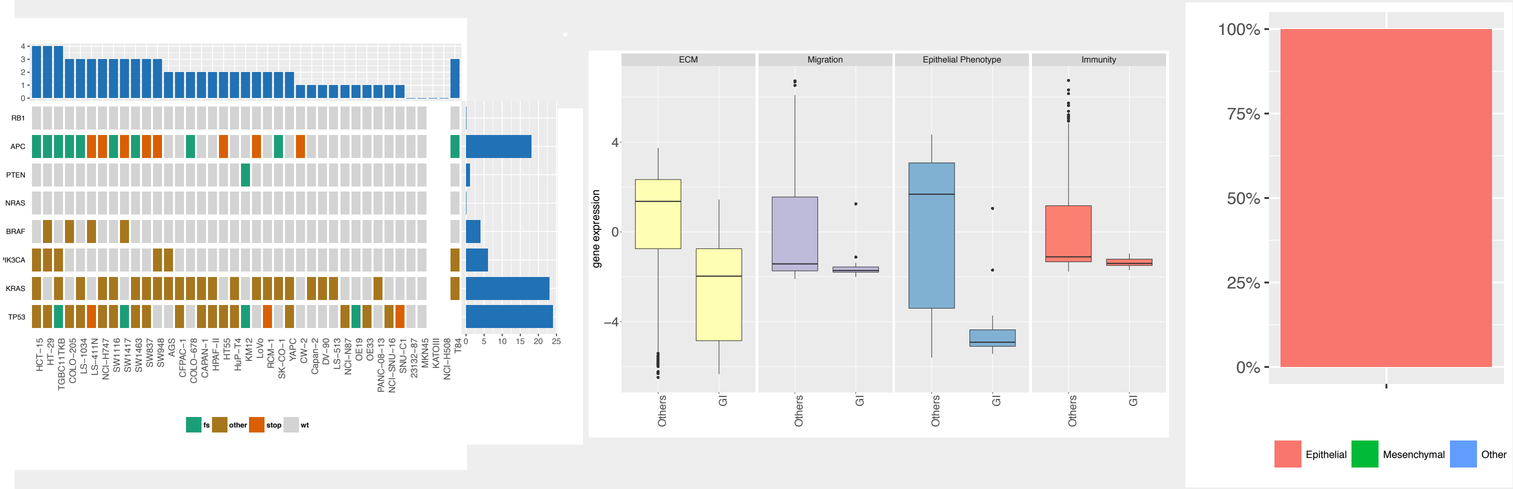

Drug profile

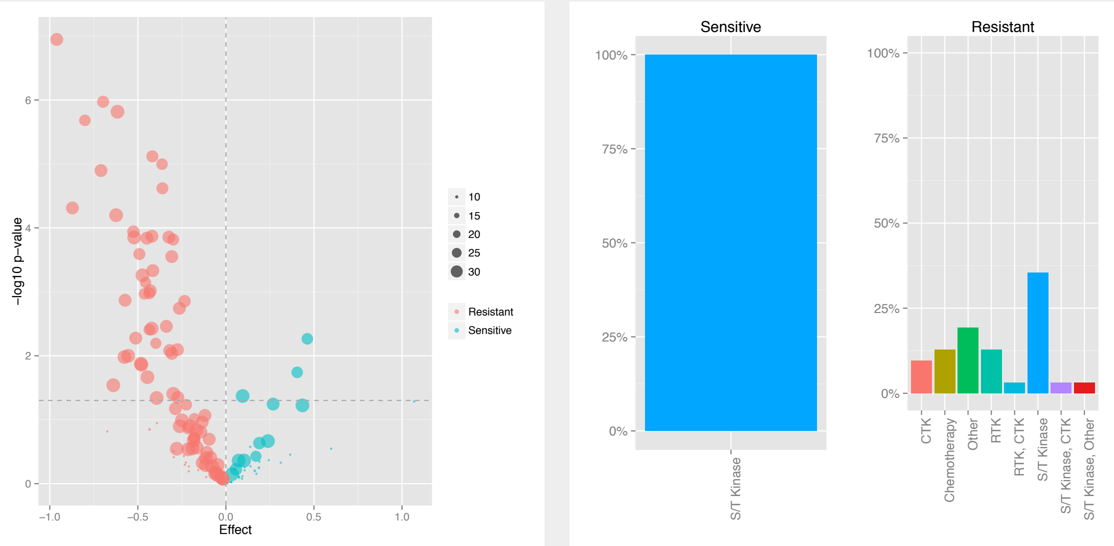

Drugs associated with the cluster (FDR$<$ **0.05**)

PD0325901	Sensible
AP–24534	Resistant
AZD6482	Resistant
DMOG	Resistant
IPA–3	Resistant
PAZOPANIB	Resistant
CAMPTOTHECIN–WTSI	Resistant
VINBLASTINE	Resistant
CISPLATIN	Resistant
DOCETAXEL	Resistant
ATRA	Resistant
NILOTINIB	Resistant
TEMSIROLIMUS	Resistant
AZD–2281	Resistant
BOSUTINIB	Resistant
AXITINIB	Resistant
CEP–701	Resistant
AMG–706	Resistant
ELESCLOMOL	Resistant
PLX4720	Resistant
BX–795	Resistant
SL–0101–1	Resistant
BI–D1870	Resistant
JNK–INHIBITOR–VIII	Resistant
681640	Resistant
PD–173074	Resistant
ZM–447439	Resistant
RO–3306	Resistant
PD0332991	Resistant
NVP–BEZ235	Resistant
GDC0941	Resistant
AZD8055	Resistant

Endodermal origin Cluster - GDSC

Cell lines in the cluster

647–V	NCI–H2009
CAL–12T	NCI–H2087
COR–L23	NCI–H2291
HT–1197	NCI–H2347
HT–1376	NCI–H358
HuCCT1	NCI–H441
LCLC–97TM1	PANC–03–27
LS–123	PANC–10–05
MKN7	
NCI–H1838	

Tissue composition

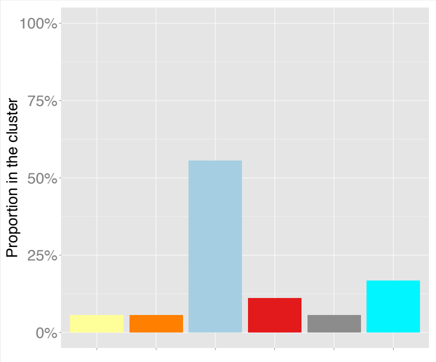

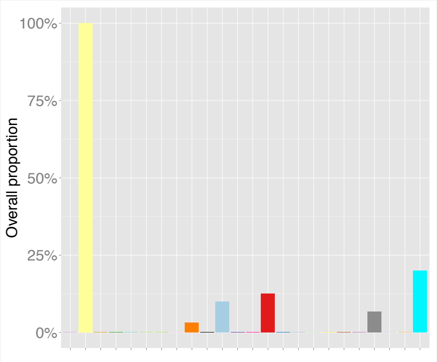

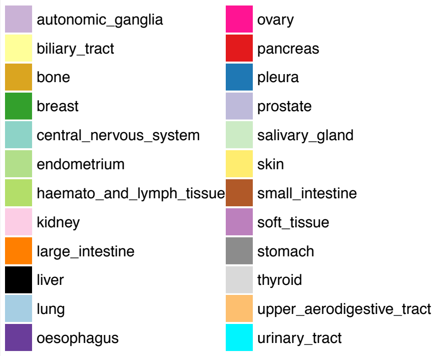

Molecular profile

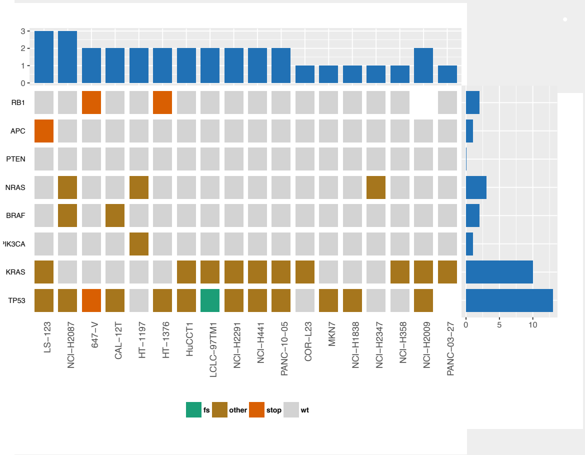

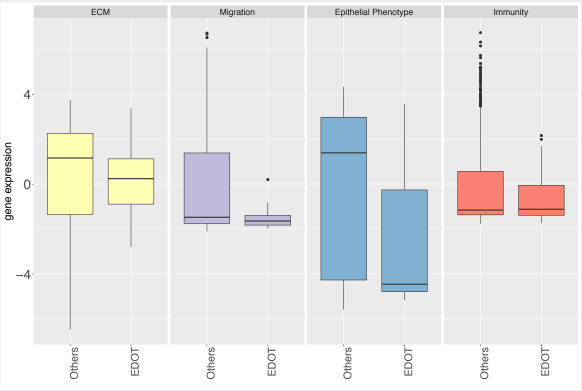

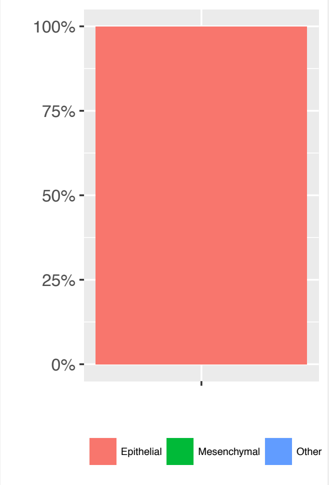

Drug profile

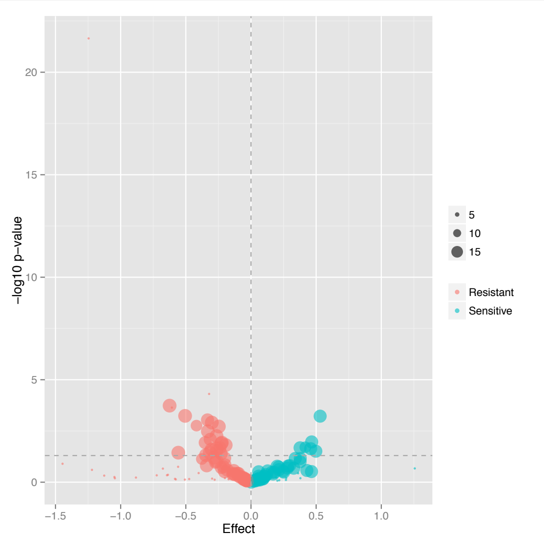

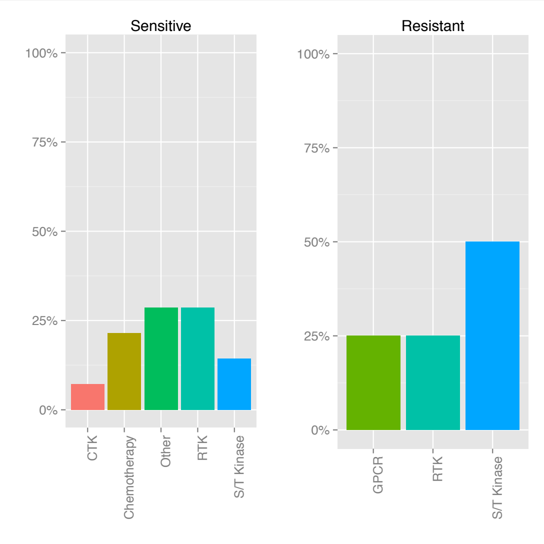

Drugs associated with the cluster (FDR< 0.05)

SUNITINIB	Sensible
CRIZOTINIB	Sensible
XMD8–85	Sensible
DMOG	Sensible
PAC–1	Sensible
IPA–3	Sensible
BAY–61–3606	Sensible
AICAR	Sensible
CAMPTOTHECIN–WTSI	Sensible
METHOTREXATE	Sensible
ATRA	Sensible
GEFITINIB	Sensible
ABT–263	Sensible
VORINOSTAT	Sensible
NILOTINIB	Sensible
TEMSIROLIMUS	Sensible
AZD–2281	Sensible
ABT–888	Sensible
BOSUTINIB	Sensible
LENALIDOMIDE	Sensible
AZD7762	Sensible
CEP–701	Sensible
VX–702	Sensible
SL–0101–1	Sensible
681640	Sensible
PD–173074	Sensible
ZM–447439	Sensible
PD0332991	Sensible
SB590885	Sensible
KIN001–135	Resistant
LAPATINIB	Resistant
NSC–87877	Resistant
BICALUTAMIDE	Resistant
FTI–277	Resistant
FH535	Resistant
BMS–754807	Resistant
BRYOSTATIN–1	Resistant
DOCETAXEL	Resistant
AZD–2281	Resistant
AXITINIB	Resistant
GW–441756	Resistant
VX–702	Resistant
MK–2206	Resistant

Mixed 1 Cluster - GDSC

Cell lines in the cluster

639–V	EFO–21	LU–99A	NCI–H1975	RKO	TE–8
8505C	ESS–1	LXF–289	NCI–H2052	SK–HEP–1	TYK–nu
AM–38	FTC–133	MDA–MB–231	NCI–H226	SK–LMS–1	UM–UC–3
BCPAP	HD–MY–Z	MIA–PaCa–2	NCI–H23	SK–LU–1	
BHT–101	HLE	MKN1	NCI–H2452	SK–UT–1	
BT–549	IA–LM	MPP–89	NCI–H596	NCI–SNU–1	
CAL–120	J82	MSTO–211H	NCI–H650	SW1573	
CAL–62	KP–4	NCI–H1299	OC–314	SW900	
Calu–1	KURAMOCHI	NCI–H1703	ONS–76	T–24	
Calu–6	LOXIMVI	NCI–H1792	OVCAR–8	TCCSUP	

Tissue composition

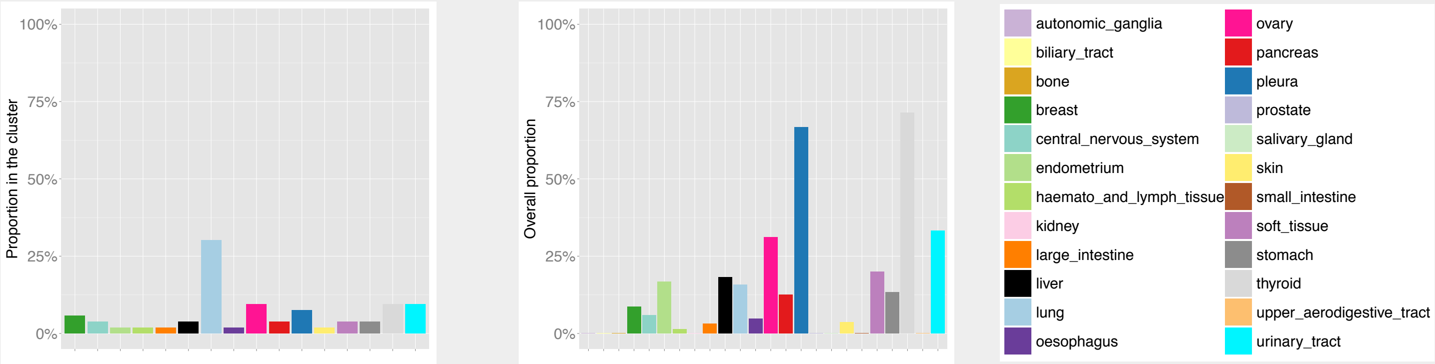

Molecular profile

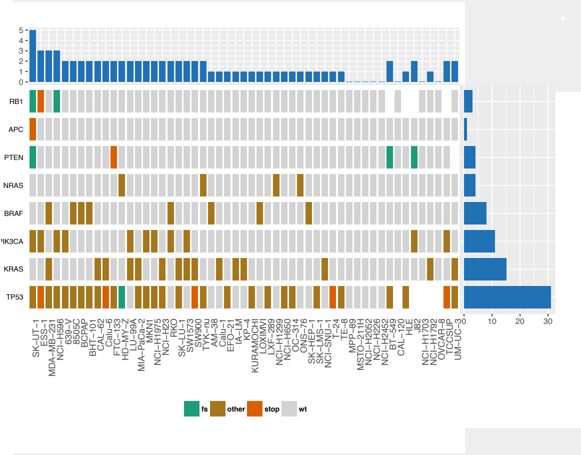

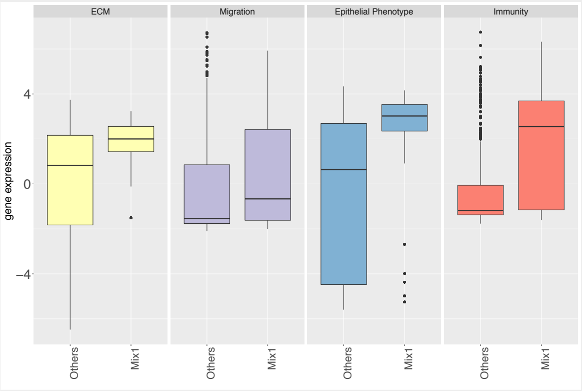

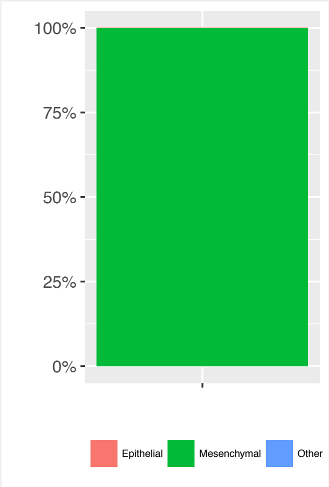

Drug profile

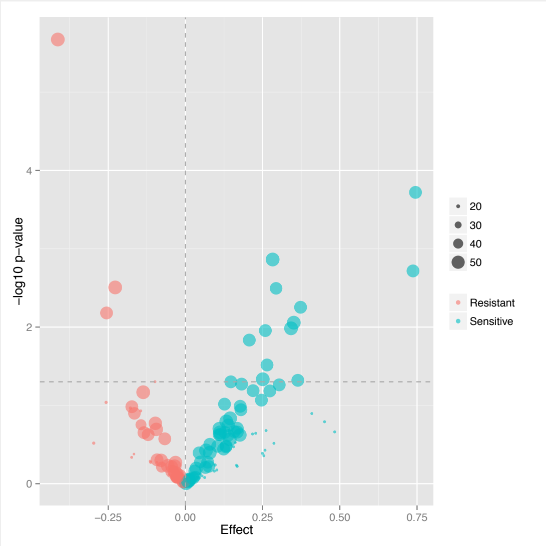

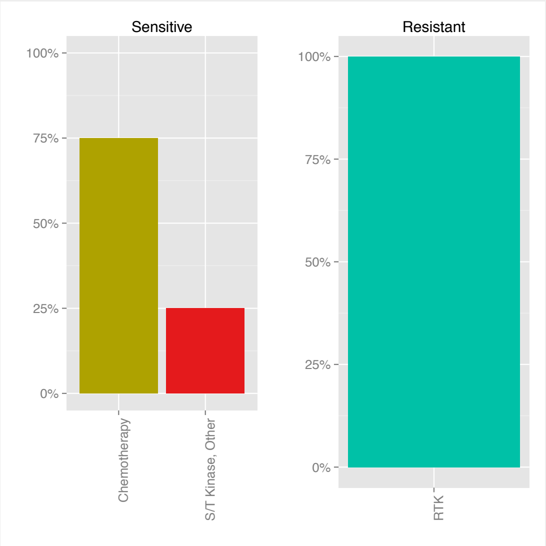

Drugs associated with the cluster (FDR$<$ **0.05**)

GEMCITABINE	Sensible
BLEOMYCIN	Sensible
VINBLASTINE	Sensible
NVP–BEZ235	Sensible
GEFITINIB	Resistant
BIBW2992	Resistant

Haematopoietic and Lymphoid tissue Cluster - GDSC

Cell lines in the cluster

697	DEL	HL–60	KMS–12–BM	MHH–CALL–4	OCI–AML2	SK–MM–2
A3–KAW	DOHH–2	HT	K052	MJ	OPM–2	SKM–1
AML–193	Daudi	HuNS1	L–363	MOLT–13	P12–ICHIKAWA	SR
BL–41	EB2	JVM–2	L–428	MOLT–16	P31–FUJ	ST486
BL–70	EHEB	JVM–3	L–540	MOLT–4	PF–382	SU–DHL–1
BV–173	EM–2	KARPAS–299	LAMA–84	MONO–MAC–6	RL	SUP–T1
CA46	GDM–1	KARPAS–422	LP–1	NALM–1	RPMI–8226	TALL–1
CMK	HDLM–2	KASUMI–1	LOUCY	NALM–6	RPMI–8402	THP–1
CML–T1	HEL	KE–37	MEG–01	NB4	RS4–11	U–266
DB	HH	KM–H2	MHH–CALL–2	NOMO–1	Raji	

Tissue composition

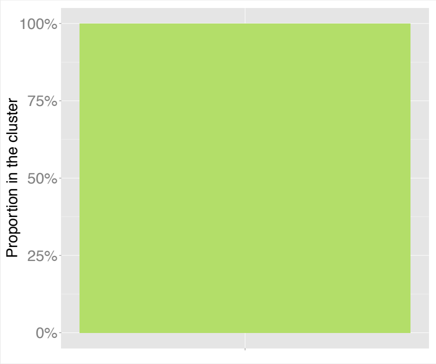

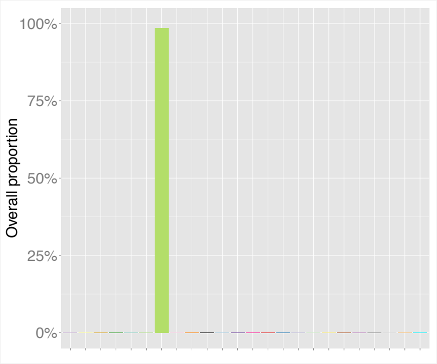

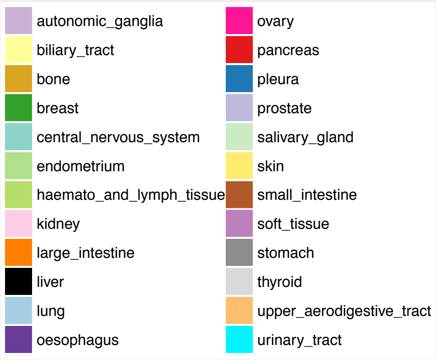

Molecular profile

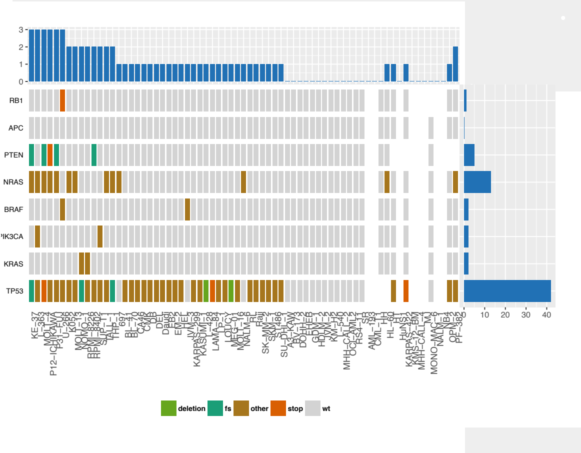

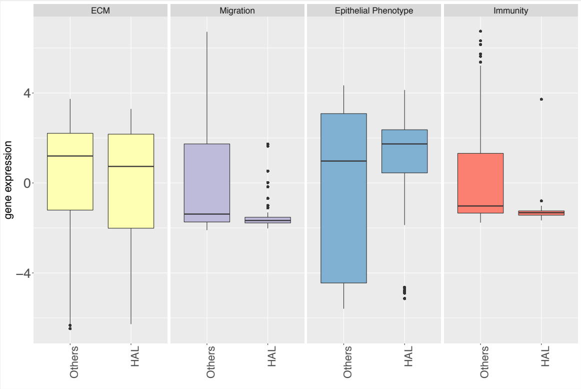

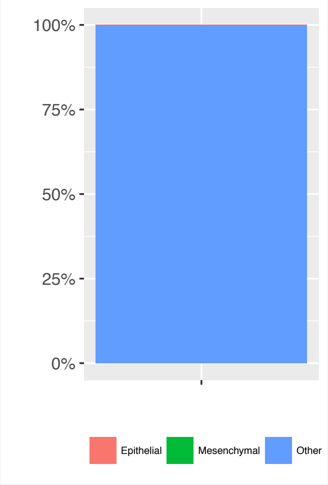

Drug profile

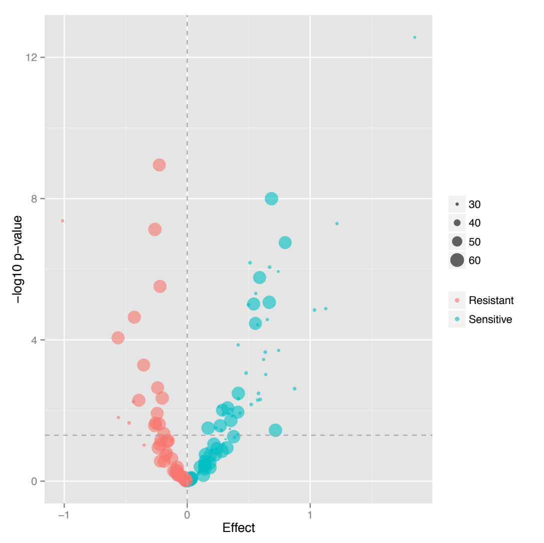

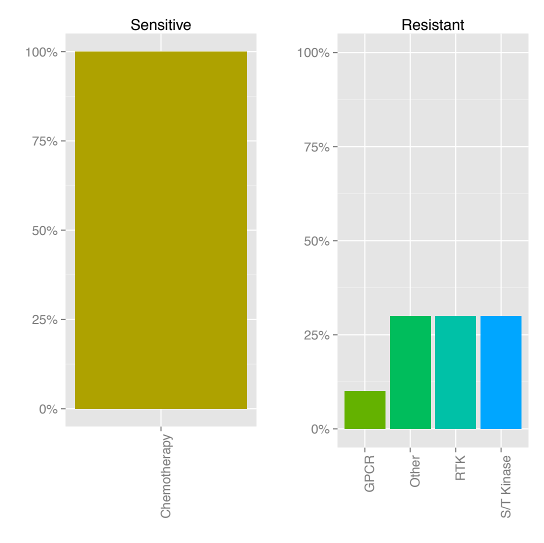

Drugs associated with the cluster (FDR< 0.05)

MIDOSTAURIN	Sensible
AICAR	Resistant
17AAG	Resistant

Mixed 2 Cluster - GDSC

Cell lines in the cluster

769–P	DU–145	NCI–H2030	RCC10RGB
786–0	EFO–27	NCI–H2228	RERF–LC–MS
A498	HEC–1	NCI–H2405	RMG–I
A704	IGROV–1	NCI–H28	SK–OV–3
A549	LU–65	NCI–H460	SNG–M
ACHN	NCI–H1355	NCI–H838	SNU–449
AsPC–1	NCI–H1563	OAW–42	NCI–SNU–5
CAL–51	NCI–H1651	OS–RC–2	SW1710
CAL–54	NCI–H1755	PLC–PRF–5	VMRC–RCZ
COR–L105	NCI–H1793	PSN1	

Tissue composition

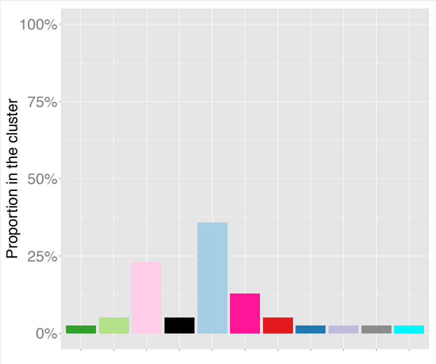

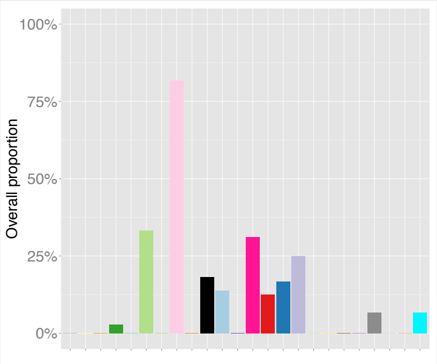

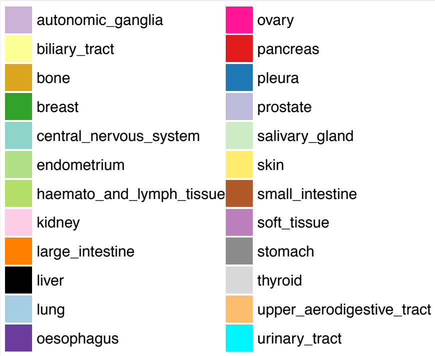

Molecular profile

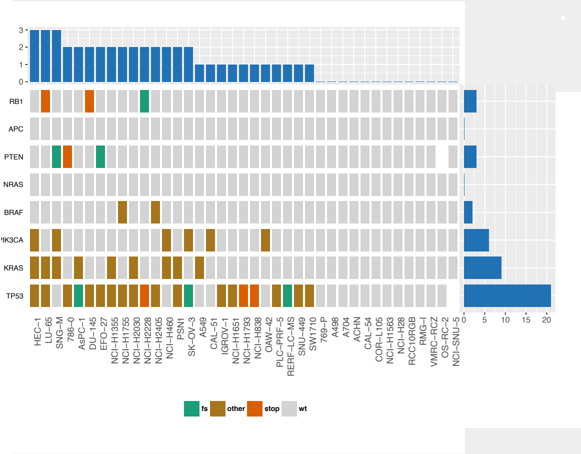

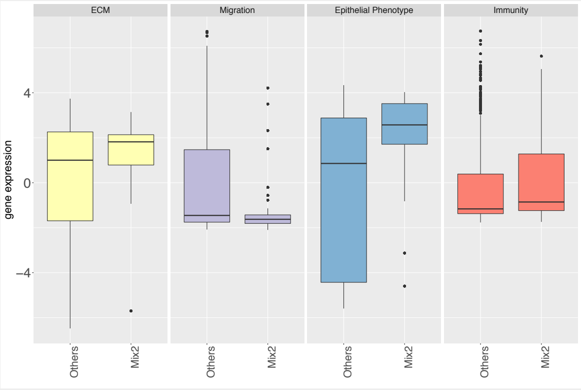

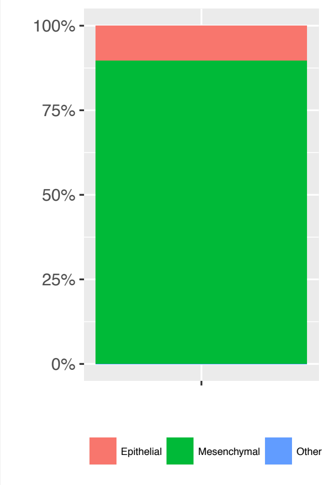

Drug profile

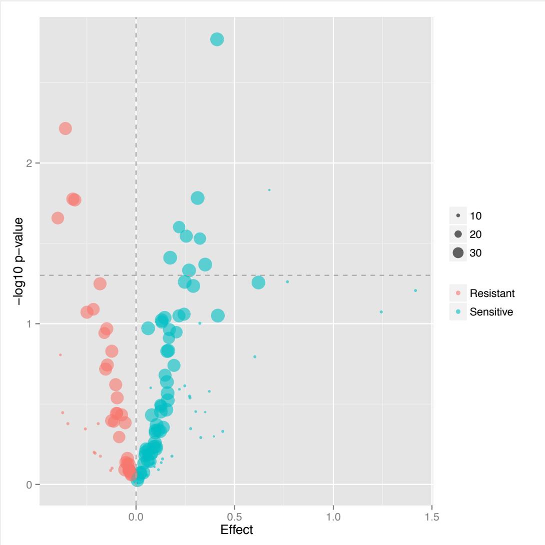

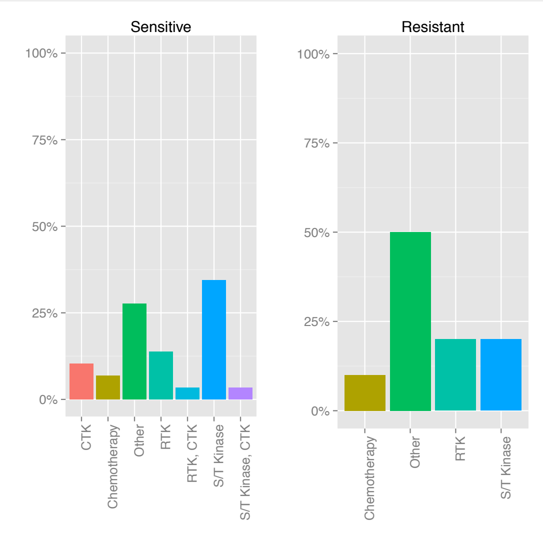

Drugs associated with the cluster (FDR< 0.05)

DASATINIB	Sensible
BORTEZOMIB	Sensible
MIDOSTAURIN	Sensible
CHIR–99021	Sensible
AZD6482	Sensible
BEXAROTENE	Sensible
LFM–A13	Sensible
PAZOPANIB	Sensible
VINBLASTINE	Sensible
DOCETAXEL	Sensible
TEMSIROLIMUS	Sensible
ELESCLOMOL	Sensible
BX–795	Sensible
NVP–BEZ235	Sensible
GDC0941	Sensible
PAC–1	Resistant
METHOTREXATE	Resistant
GEFITINIB	Resistant

Glioma and Sarcoma Cluster - GDSC

Cell lines in the cluster

8–MG–BA, A172, BFTC–909, Becker, CAS–1, CCF–STTG1, CGTH–W–1, DBTRG–05MG, DK–MG, Daoy,
GAMG, GCT, GI–1, GMS–10, H4, HCC1395, HOS, HT–1080, Hs–578–T, HuH–28,
IST–MES1, KALS–1, KNS–42, KNS–81–FD, KS–1, LCLC–103H, MDA–MB–157, MG–63, MOG–G–CCM, MOG–G–UVW,
NMC–G1, RPMI–7951, S–117, SF295, SF126, SJSA–1, SK–MES–1, SNB19, SNU–387, SNU–423,
SNU–475, SW1088, SW1783, TI–73, T98G, U–118–MG, U–2–OS, U251, U–87–MG, WM–115,
YH–13, YKG–1

Tissue composition

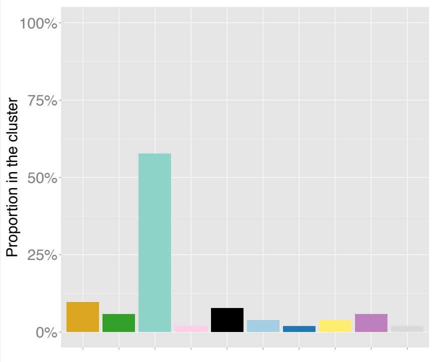

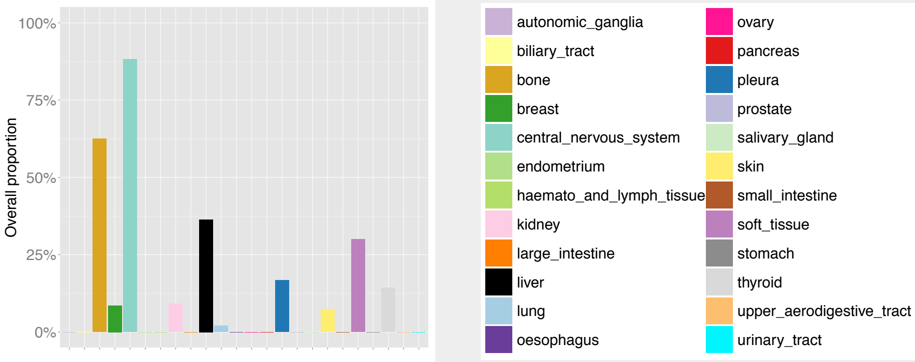

Molecular profile

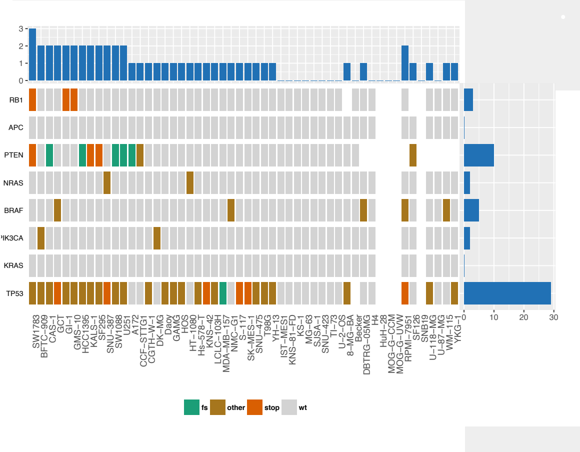

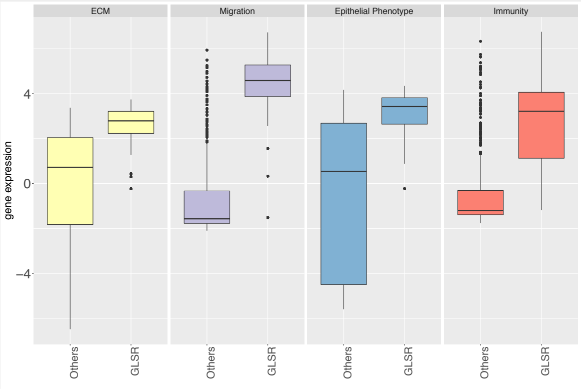

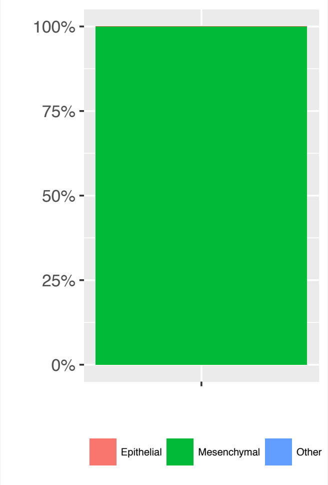

Drug profile

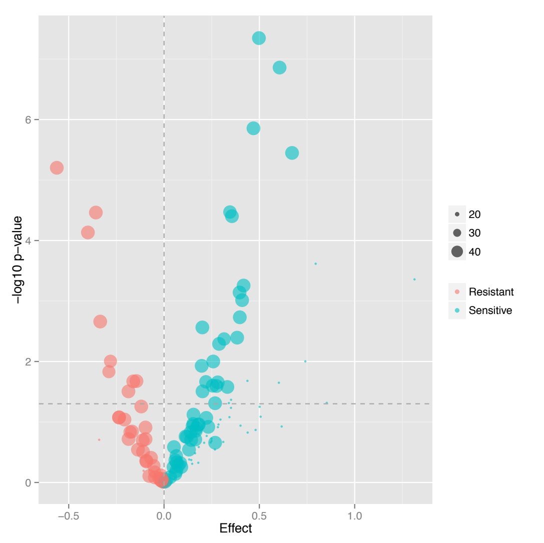

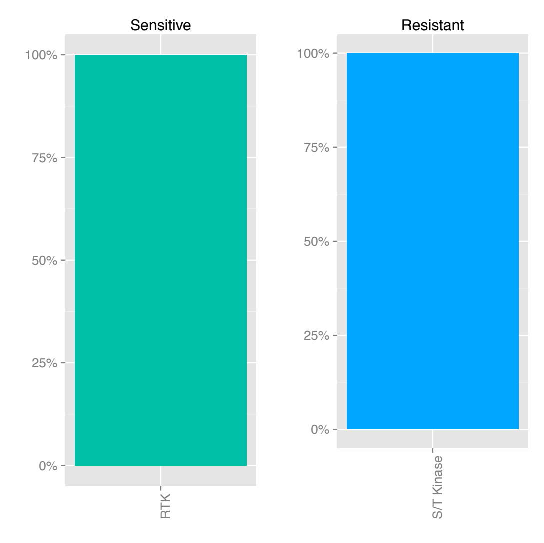

Drugs associated with the cluster (FDR< 0.05)

VORINOSTAT	Sensible
Z–LLNLE–CHO–	Resistant
DASATINIB	Resistant
BICALUTAMIDE	Resistant
BIBW2992	Resistant

Mixed 3 Cluster - GDSC

Cell lines in the cluster

A204	DMS–273	MHH–ES–1	NCI–H82	SK–N–FI
A673	ECC12	MHH–NB–11	NH–6	Saos–2
A2780	G–401	NB1	RD	
AN3–CA	HGC–27	NCI–H1155	RH–18	
CHL–1	HUTU–80	NCI–H2227	SBC–5	
CHP–212	KP–N–RT–BM–1	NCI–H446	SHP–77	
COLO–320–HSR	KP–N–S19s	NCI–H522	SIMA	
CPC–N	KP–N–YN	NCI–H524	SJRH30	
D–283MED	MES–SA	NCI–H661	SK–N–AS	
DMS–114	MFE–296	NCI–H716	SK–N–DZ	

Tissue composition

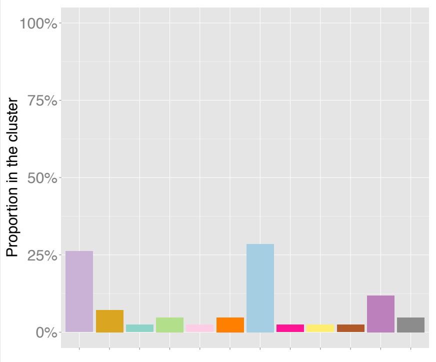

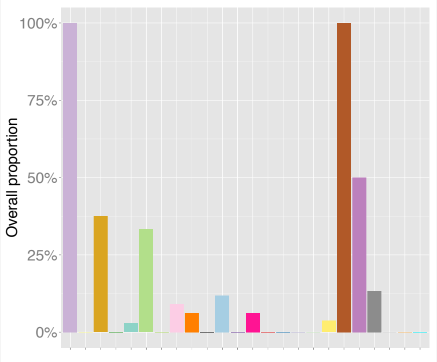

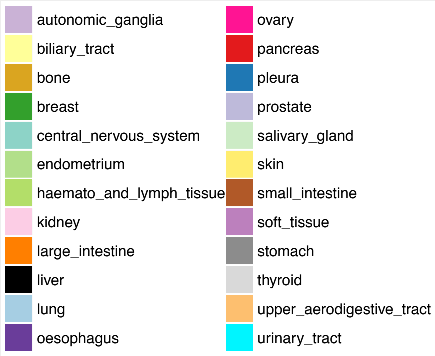

Molecular profile

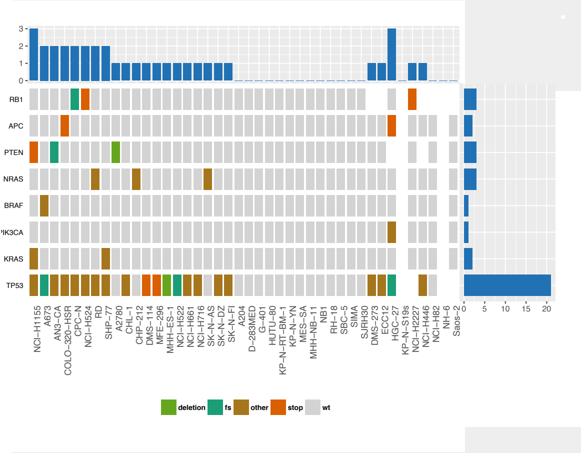

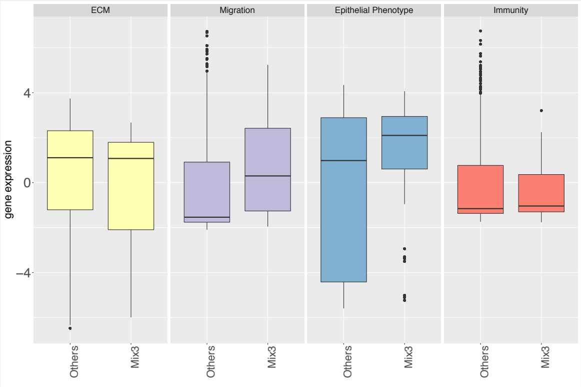

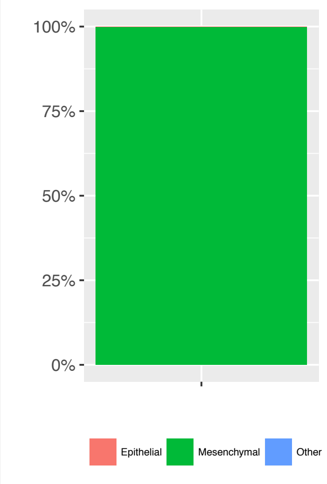

Drug profile

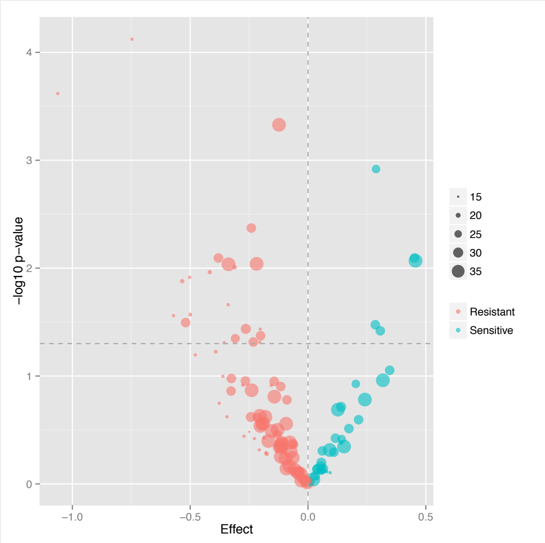

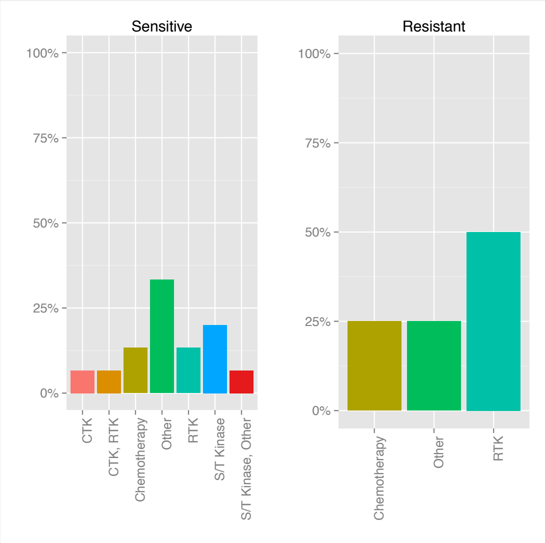

Drugs associated with the cluster (FDR< 0.05)

ERLOTINIB	Sensible
LAPATINIB	Sensible
VINORELBINE	Sensible
BICALUTAMIDE	Sensible
PF–562271	Sensible
OBATOCLAX–MESYLATE	Sensible
EPOTHILONE–B	Sensible
AICAR	Sensible
VINBLASTINE	Sensible
DOCETAXEL	Sensible
GEFITINIB	Sensible
BOSUTINIB	Sensible
17AAG	Sensible
BIBW2992	Sensible
CYCLOPAMINE	Resistant
GSK269962A	Resistant
PAZOPANIB	Resistant
SB–216763	Resistant

Aerodigestive tract Cluster - GDSC

Cell lines in the cluster

5637	Detroit562	KNS–62	KYSE–70	SCC–4	TE–15
A253	EC–GI–10	KU–19–19	LC–1F	SCC–9	TE–5
BFTC–905	EPLC–272H	KYSE–140	NCI–H1666	SW1990	TE–6
BHY	FADU	KYSE–150	NCI–H292	SW780	TE–9
BxPC–3	HCC1806	KYSE–180	NUGC–3	SW403	VM–CUB–1
CAL–27	HCT–116	KYSE–270	PC–14	SW48	
CAL–33	HSC–2	KYSE–410	PC–3	SW620	
COLO–680N	HSC–3	KYSE–450	RT–112	TE–1	
Calu–3	HSC–4	KYSE–510	SCC–15	TE–10	
DU–4475	HuP–T3	KYSE–520	SCC–25	TE–11	

Tissue composition

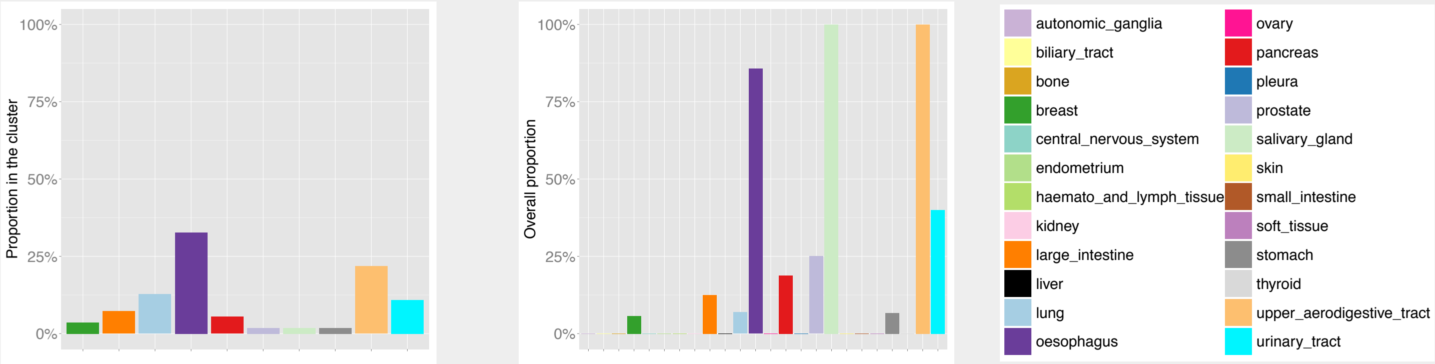

Molecular profile

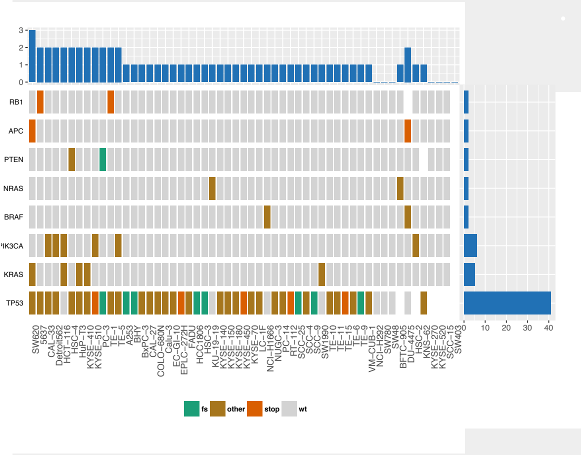

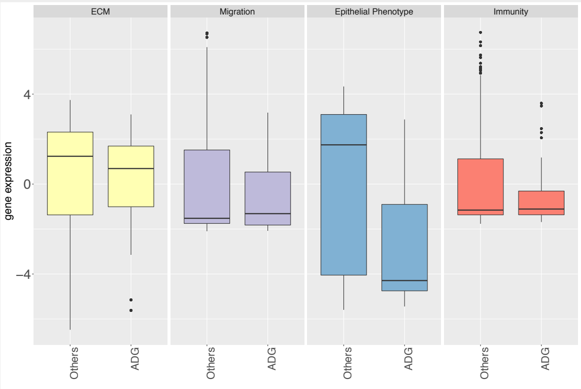

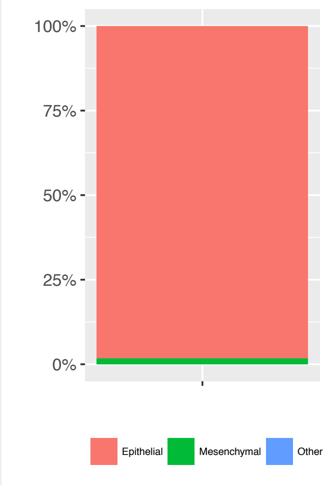

Drug profile

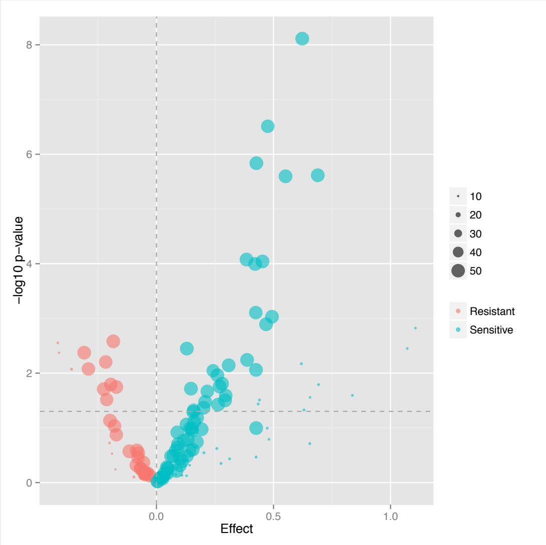

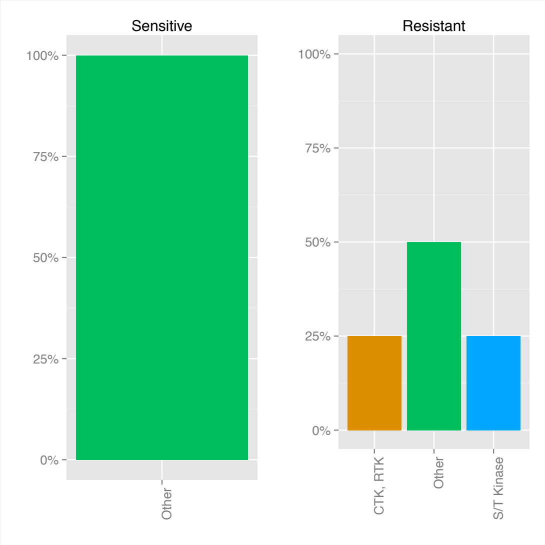

Drugs associated with the cluster (FDR< 0.05)

DOXORUBICIN	Sensible
CYCLOPAMINE	Resistant
CRIZOTINIB	Resistant
PYRIMETHAMINE	Resistant
ATRA	Resistant
TEMSIROLIMUS	Resistant
VX–702	Resistant

Skin Cluster - GDSC

Cell lines in the cluster

A375	IGR−1	SK−MEL−3
A101D	IPC−298	SK−MEL−30
A2058	MEL−HO	SK−MEL−5
C32	MEL−JUSO	UACC−257
COLO−741	Malme−3M	UACC−62
COLO−792	Mewo	
COLO−679	RVH−421	
COLO−800	SK−MEL−2	
G−361	SK−MEL−24	
HT−144	SK−MEL−28	

Tissue composition

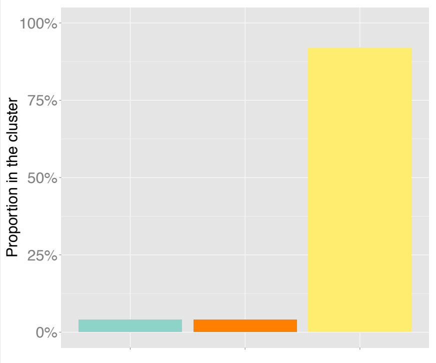

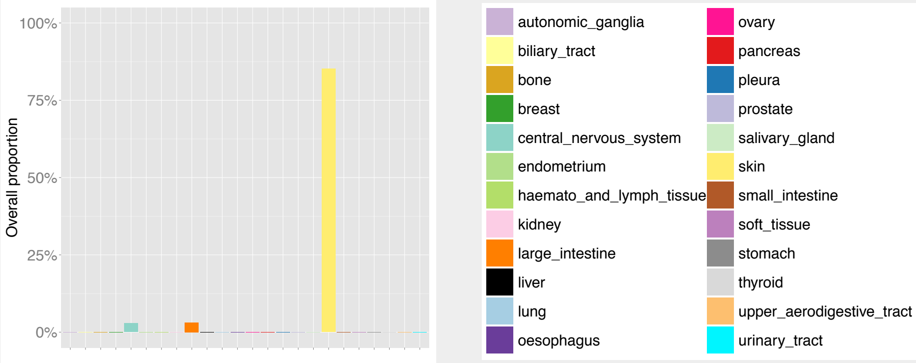

Molecular profile

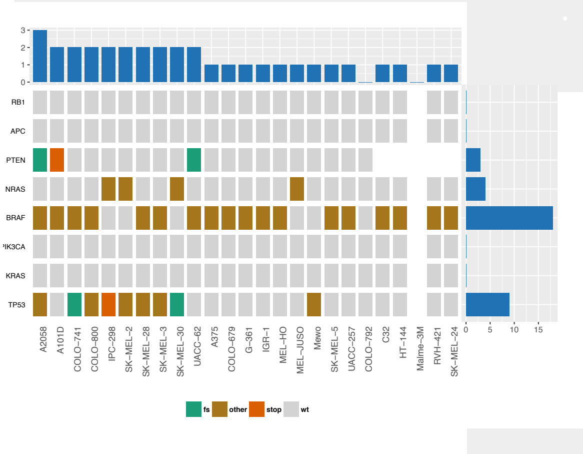

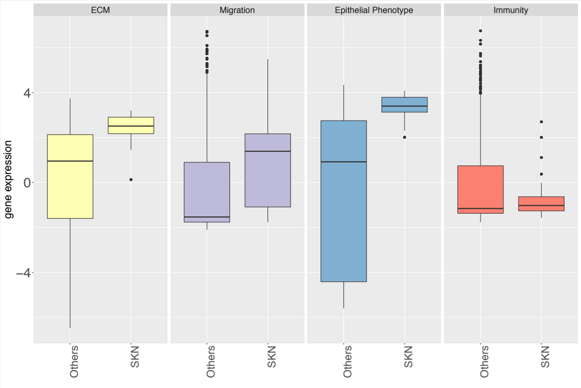

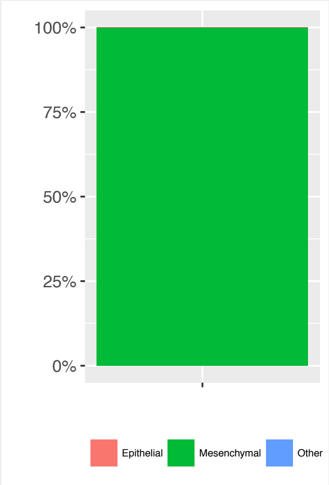

Drug profile

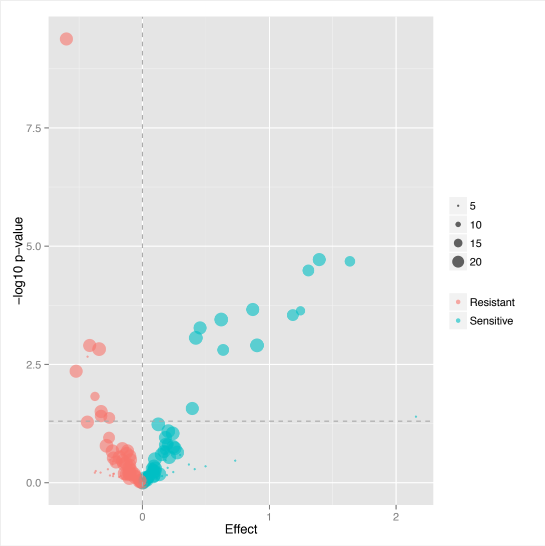

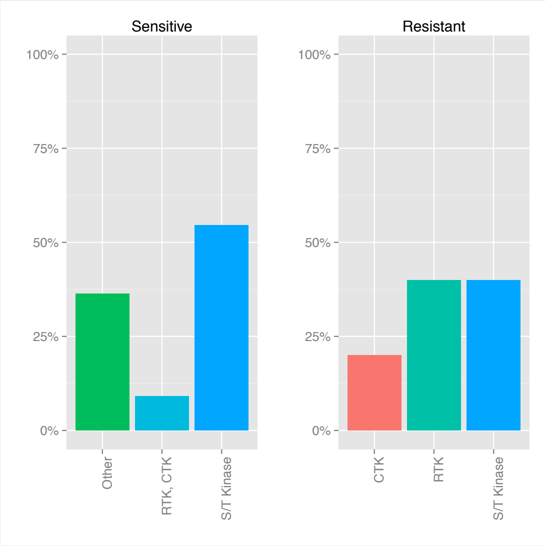

Drugs associated with the cluster (FDR< 0.05)

EMBELIN	Sensible
FH535	Sensible
OBATOCLAX−MESYLATE	Sensible
RDEA119	Sensible
CI−1040	Sensible
CEP−701	Sensible
PLX4720	Sensible
NUTLIN3	Sensible
PD0325901	Sensible
SB590885	Sensible
AZD6244	Sensible
WZ−1−84	Resistant
AKT−INHIBITOR−VIII	Resistant
AICAR	Resistant
GEFITINIB	Resistant
BIBW2992	Resistant

Breast Cluster - GDSC

Cell lines in the cluster

ABC–1	GCIY	HCC70	NCI–H1573	UACC–812
BT–20	HCC1143	MCF7	NCI–H1650	ZR–75–30
CAL–148	HCC1187	MDA–MB–175–VII	NCI–H1693	
CAL–85–1	HCC1419	MDA–MB–361	NCI–H2126	
CAMA–1	HCC1599	MDA–MB–415	NCI–H2170	
Caov–3	HCC1937	MDA–MB–453	NCI–H2342	
Caov–4	HCC1954	MDA–MB–468	OVCAR–3	
ChaGo–K–1	HCC2157	MFE–280	OAW–28	
EFM–19	HCC2218	NCI–H1395	OVCAR–4	
EVSA–T	HCC38	NCI–H1437	T47D	

Tissue composition

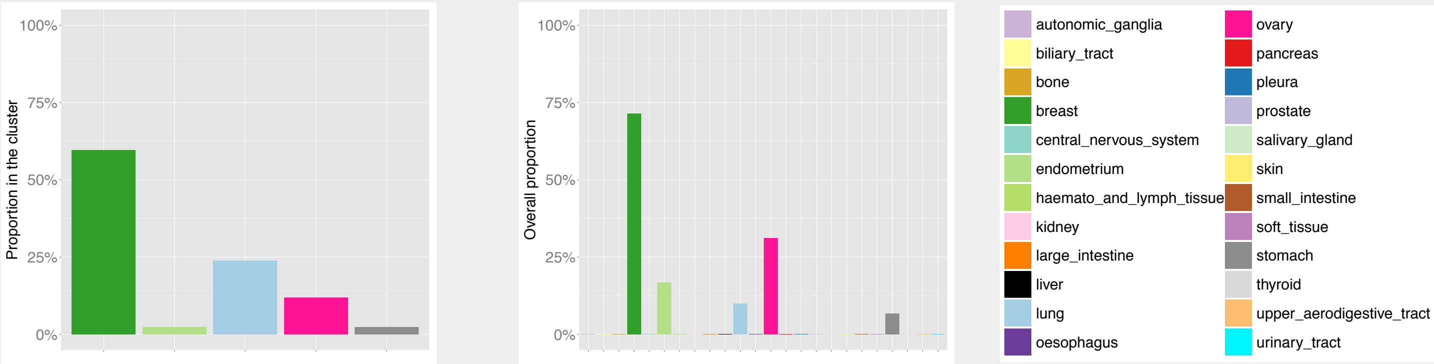

Molecular profile

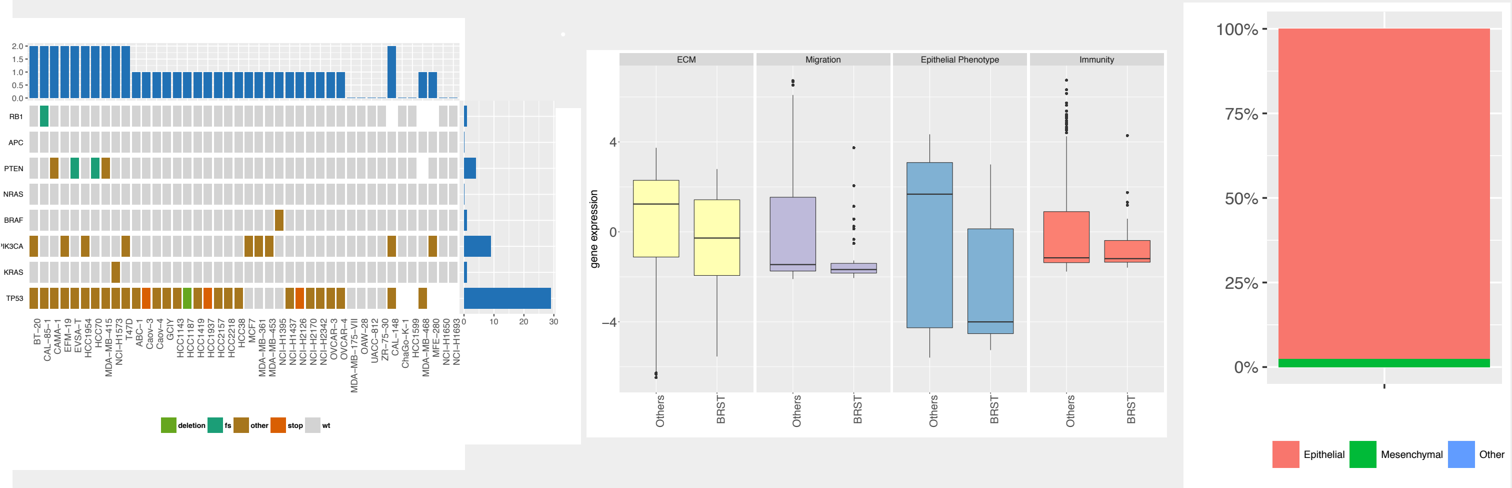

Drug profile

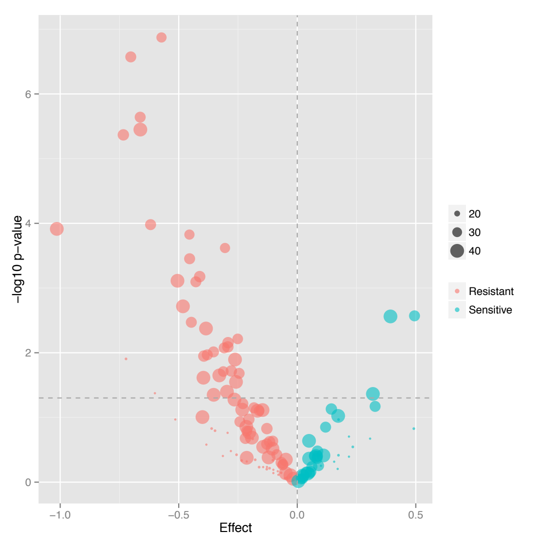

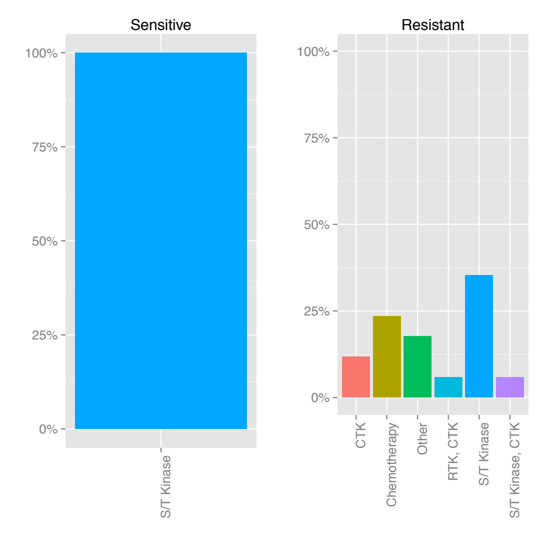

Drugs associated with the cluster (FDR< 0.05)

AKT–INHIBITOR–VIII	Sensible
MK–2206	Sensible
GEMCITABINE	Resistant
AP–24534	Resistant
DMOG	Resistant
BAY–61–3606	Resistant
OBATOCLAX–MESYLATE	Resistant
CAMPTOTHECIN–WTSI	Resistant
CYTARABINE	Resistant
METHOTREXATE	Resistant
RDEA119	Resistant
CI–1040	Resistant
AZD–2281	Resistant
CEP–701	Resistant
SB–216763	Resistant
BX–795	Resistant
BI–D1870	Resistant
681640	Resistant
AZD6244	Resistant
